# Epidermal Tissue Adapts to Restrain Progenitors Carrying Clonal *p53* Mutations

**DOI:** 10.1016/j.stem.2018.08.017

**Published:** 2018-11-01

**Authors:** Kasumi Murai, Greta Skrupskelyte, Gabriel Piedrafita, Michael Hall, Vasiliki Kostiou, Swee Hoe Ong, Tibor Nagy, Alex Cagan, David Goulding, Allon M. Klein, Benjamin A. Hall, Philip H. Jones

**Affiliations:** 1 Wellcome Sanger Institute, Hinxton CB10 1SA, UK; 2 MRC Cancer Unit, University of Cambridge, Hutchison-MRC Research Centre, Cambridge Biomedical Campus, Cambridge CB2 0XZ, UK; 3 Department of Systems Biology, Harvard Medical School, Harvard Medical School, Boston, MA 02115, USA

**Keywords:** cell competition, ultraviolet light, carcinogenesis, Trp53, desmosome, Cdh1, cell adhesion, differentiation, stem cell, transgenic mice

## Abstract

Aging human tissues, such as sun-exposed epidermis, accumulate a high burden of progenitor cells that carry oncogenic mutations. However, most progenitors carrying such mutations colonize and persist in normal tissue without forming tumors. Here, we investigated tissue-level constraints on clonal progenitor behavior by inducing a single-allele *p53* mutation (*Trp53*^*R245W*^*; p53*^*∗/wt*^), prevalent in normal human epidermis and squamous cell carcinoma, in transgenic mouse epidermis. *p53*^*∗/wt*^ progenitors initially outcompeted wild-type cells due to enhanced proliferation, but subsequently reverted toward normal dynamics and homeostasis. Physiological doses of UV light accelerated short-term expansion of *p53*^*∗/wt*^ clones, but their frequency decreased with protracted irradiation, possibly due to displacement by UV-induced mutant clones with higher competitive fitness. These results suggest multiple mechanisms restrain the proliferation of *p53*^*∗/wt*^ progenitors, thereby maintaining epidermal integrity.

## Highlights

• *p53* mutant progenitors outcompete wild-type cells to colonize normal epidermis
• The epidermis adapts to the mutant clones, slowly returning toward normal
• Low-dose UV light drives *p53* mutant clone expansion in the short term
• Prolonged UV exposure generates mutant clones that may outcompete *p53* mutant cells

## Graphical Abstract

**Figure:**
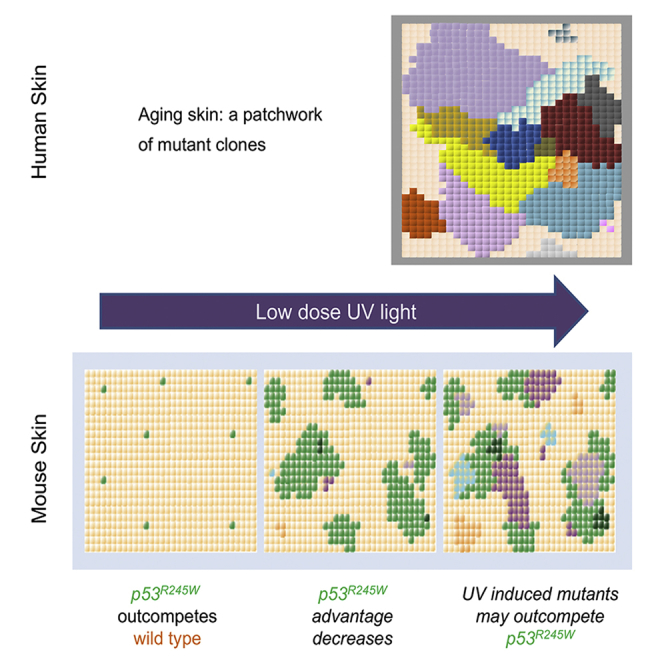

## Teaser

Normal human epidermis harbors *p53* mutant clones, very few of which form tumors. Murai et al. track *p53* mutant clones as they colonize the epidermis in a transgenic mouse model showing how the tissue adapts to the mutant cells and uncovering distinct roles for physiological UV light exposure.

## Introduction

Proliferating human tissues harbor substantial populations of stem/progenitor cells carrying somatic mutations linked to neoplasia and other diseases (Alexandrov et al., 2015, Jaiswal et al., 2017). This process is exemplified by the epidermis, which is exposed to UV light over decades and accumulates a high proportion of progenitors carrying UV-induced oncogenic mutations under strong evolutionary selection (Martincorena et al., 2015). For example, 4%–14% of epidermal cells in sun-exposed human skin carry non-synonymous mutations of the tumor suppressor gene *p53 (TP53) (*Jonason et al., 1996, Martincorena et al., 2015). Despite this, aging epidermis remains histologically and functionally normal, and, in comparison with the size of the mutant progenitor population, the incidence of keratinocyte cancers is exceedingly low. Here, we investigate the mechanisms that constrain *p53* mutant progenitors and underpin the remarkable resilience of the epidermis to mutation.

**Figure 1:**
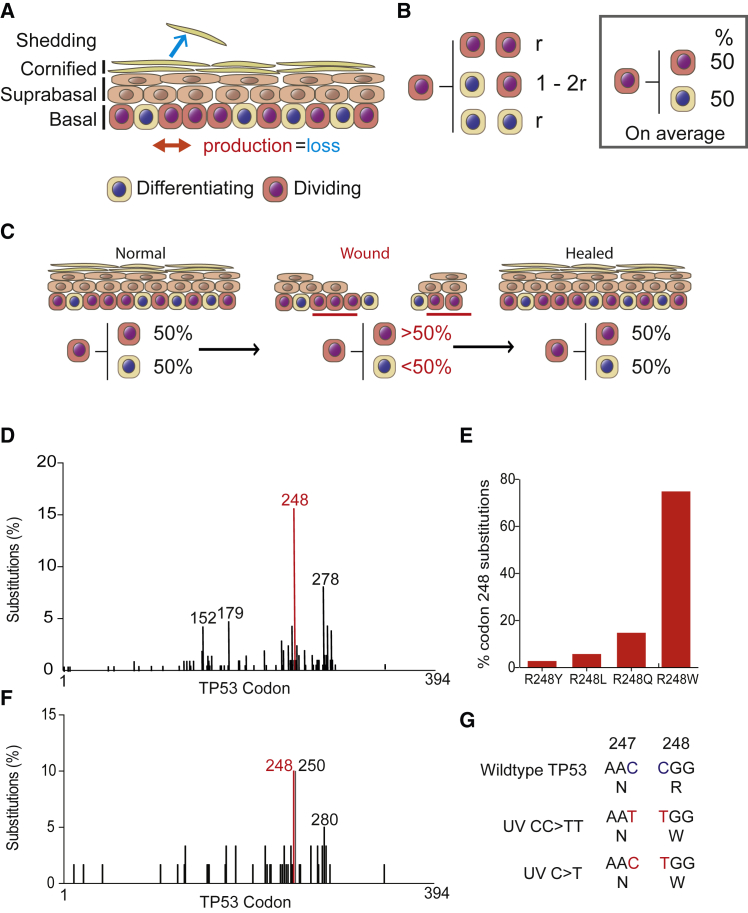
Cell Behavior in the Epidermis and *p53* Mutations (A) Interfollicular epidermis (IFE). The tissue consists of layers of keratinocytes. Proliferation is confined to the basal cell layer. Differentiating basal cells exit the cell cycle and then stratify out of the basal layer, migrating through the suprabasal and cornified layers to the surface from which they are shed. In normal IFE, the rate of cell production in the basal layer (red arrow) is the same as the rate of cell loss by shedding (blue arrow). (B) Single-progenitor model of IFE homeostasis. All dividing basal cells are functionally equivalent progenitor cells (pink). On division, a progenitor may generate two progenitors, two differentiating progeny that will cease division and stratify (beige) or one cell of each type. The outcome of a given division is unpredictable, but the likelihood (r) of producing two progenitor or two differentiating daughters is the same, so that, on average, across the population, equal proportions of progenitor and differentiating cells are generated (box). (C) Plasticity of epidermal progenitors. Following wounding, the progenitors adjacent to the injury (red bars) switch from homeostatic behavior to producing more progenitor than differentiating progeny, until the wound is healed, and then they revert to homeostasis; numbers indicate percentages of cells generated per average cell division in each state. (D) Distribution of TP53 missense mutations in cutaneous squamous cell carcinoma (data from COSMIC v.79, https://cancer.sanger.ac.uk/cosmic). (E) Frequency of TP53 Codon 248 amino acid changes in cutaneous squamous cell carcinoma. (F) Distribution of TP53 missense mutations in normal, sun-exposed human epidermis. Data from Martincorena et al., 2015. (G) The two modes of generating TP53^R248W^ codon change from UV-signature mutations.

The epidermis consists of layers of keratinocytes punctuated by hair follicles and sweat ducts (Alcolea and Jones, 2014). Keratinocytes are continually shed from the tissue surface and replaced by proliferation in the basal cell layer (Figure 1A). On commitment to terminal differentiation, proliferating basal cells exit the cell cycle and migrate into the suprabasal cell layers. They then undergo a sequence of changes in gene expression and cell morphology and are ultimately shed as anucleate cornified cells. Throughout life the epidermis self- renews, matching cell production in the basal layer with cell loss from the epidermal surface (Roshan and Jones, 2012).

Various models of normal epidermal homeostasis have been proposed (Allen and Potten, 1974, Sada et al., 2016). Multiple lineage tracing and intravital imaging studies suggest the interfollicular epidermis (IFE) is maintained by a single population of progenitor cells with stochastic fate (Clayton et al., 2007, Doupé et al., 2010, Lim et al., 2013, Rompolas et al., 2016, Roshan et al., 2016). In this paradigm, progenitor cells divide to generate two progenitor daughters, two non-dividing differentiating cells or one cell of each type (Figure 1B). The outcome of individual progenitor cell divisions is unpredictable, but the probability of generating differentiated or proliferating cells is balanced. As a result, the average cell division generates one progenitor and one differentiating daughter cell across the progenitor population, achieving cellular homeostasis and ensuring the majority of clones with mutations that do not alter cell dynamics are lost by differentiation and subsequent shedding (Figure 1B) (Clayton et al., 2007).

Epidermal progenitor fate is adaptable, enabling cells to both sustain normal cell turnover and respond to injury (Lim et al., 2013, Park et al., 2017, Roshan et al., 2016). Following wounding, nearby progenitors produce more proliferating than differentiating daughters until the epidermis is repaired, after which they revert to normal, balanced behavior (Figure 1C). While the ability to modulate progenitor cell fate provides a robust mechanism of tissue repair, it also represents a potential vulnerability, as somatic mutations may drive progenitors to produce an excess of proliferating cells in the absence of injury (Alcolea et al., 2014). Unchecked, even a small bias toward proliferation may lead to clonal dominance and eventually tumor formation (Frede et al., 2016).

Here, we focus on a *p53* mutant detected in normal human epidermis, *p53*^*R248W*^ (Figures 1D–1G, (https://cancer.sanger.ac.uk/cosmic/) (Martincorena et al., 2015). This mutation is frequently found in human squamous cell carcinoma and has gain-of-function (GOF) properties, distinct from those of a *p53*-null allele (Muller and Vousden, 2014, Song et al., 2007). *p53*^*R248W*^ is a contact mutant with altered DNA-binding properties and acts in dominant-negative manner over wild-type *p53* when they are co-expressed (Song et al., 2007). Several studies from cellular systems and humanized mouse models showed that *p53*^*R248W*^ plays a role in the cancer phenotype through increased proliferation, drug resistance, migration, and chromosomal instability (Song et al., 2007, Zhu et al., 2015). However, recent studies suggest that some *p53* mutants including p53^R248W^ exhibit GOF attributes only in cancer cells of particular lineages and the impact of the *p53*^*R248W*^ mutation on normal cell behavior is unclear (Sabapathy, 2015). We hypothesized that *p53*^*R248W*^ may alter normal progenitor behavior to increase the likelihood of clonal persistence and the acquisition of additional mutations.

To test this hypothesis, we used a mouse model. Carcinogenesis in mouse skin has been studied extensively, and, like humans, mice may harbor mutated cells long term within normal epidermis (Berenblum and Shubik, 1949, Zhang et al., 2001). We developed a transgenic strain to track the fate of individual epidermal progenitor cells following induction of *Trp53*^*R245W*^, the murine equivalent of human *TP53*^*R248W*^, in a background of wild-type cells, and the impact of physiological doses of UV light on mutant cell behavior.

## Results

### Wild-Type Progenitor Behavior in Dorsal Epidermis

We began by characterizing the behavior of *p53* wild-type (*p53*^*wt/wt*^*)* progenitors in dorsal murine IFE labeled with a neutral genetic reporter by the *Ahcre*^*ERT*^-inducible *cre* recombinase line. A conditional Yellow Fluorescent Protein (YFP) allele was induced at a low frequency in basal cells of *Ahcre*^*ERT*^*Rosa26*^*flYFP/wt*^ (*R-YFP*) mice (Clayton et al., 2007, Kemp et al., 2004) (Figures S1A, S2A, and S2B). Animals were sacrificed at different times and the number and location of cells in YFP-expressing (YFP^+^) clones determined by confocal imaging (Figures S2A–S2C) (Clayton et al., 2007, Doupé et al., 2010, Page et al., 2013). Perifollicular clones adjacent to hair follicle openings were excluded. The clonal data displayed the quantitative hallmarks of a single population of progenitors with stochastic fate (Figures S2D–S2H; STAR Methods) (Clayton et al., 2007, Doupé et al., 2010, Klein et al., 2007).

To further explore the kinetics of keratinocyte turnover, we used a transgenic cell proliferation assay based on H2BGFP dilution over a period of up to 24 weeks (Figures S1B and S2I) (Doupé et al., 2012, Sada et al., 2016). Keratinocytes showed a constant and homogeneous dilution pattern in a timescale of days. These data indicated that cycling keratinocytes divided at a similar rate (Figures S2K–S2M; STAR Methods), arguing that growth arrest and senescence, two of the stress responses mediated by *p53*, are rare in wild-type IFE (Figure S2J) (Muller and Vousden, 2014). Altogether, both lineage tracing and cell proliferation data were consistent with dorsal IFE being maintained by a single population of progenitors with balanced stochastic fate (Figures 1B and S3; STAR Methods).

### Inducible *p53*^*R245W/wt*^ Transgenic Mice

**Figure 2:**
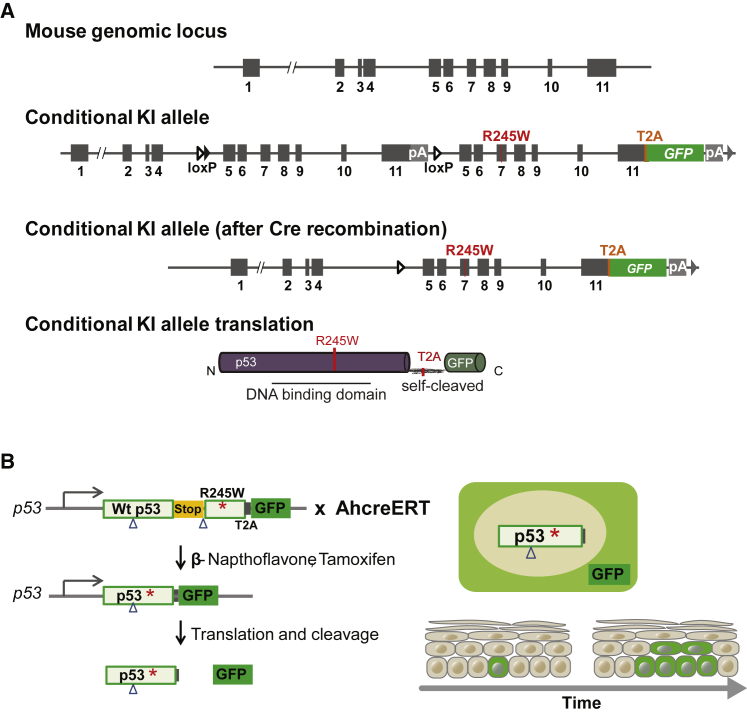
Conditional *p53* Mutant Transgenic Mouse Model (A) Schematic illustration of targeting construct. *Trp53* exons 5–11 were flanked by *loxP* sites (triangle). pA indicates an additional transcriptional STOP cassette. The engineered *Trp53* duplicate region contains R245W mutations in exon 7, a C-terminal in-frame self-cleaving T2A peptide and enhanced GFP (eGFP). The conditional knockin (cKI) *Trp53* allele was obtained after *Flp*-mediated deletion of the selection markers. Closed triangle and gray triangle indicate *FRT* site and *F3* site, respectively. Prior to induction, the cKI allele expresses the wild-type *Trp53* protein; however, after *cre*-mediated recombination, the allele co-expresses *Trp53*^*R245W*^ mutant protein and eGFP. (B) Genetic lineage tracing in *Ahcre*^*ERT*^*p53*^*∗/wt*^ mice. *Cre*-mediated recombination, induced by β-napthoflavone (βNF) and tamoxifen (Tam), results in expression of the mutant protein in place of the wild-type. The heritable expression of the mutant protein and GFP reporter allows the study of competition between mutant clones and the surrounding wt cells. See also Figures S4 and S5 and Table S1.

To determine whether induction of a heterozygous *p53*^*R245W*^ allele altered progenitor cell behavior, we developed a new conditional mouse strain, *Trp53*^*fl-R245W-GFP/wt*^ (henceforth referred to as *p53^∗^*^*/wt*^). A conditional allele of *p53^∗^*, with a GFP reporter linked to the C terminus of the p53^∗^ protein by a T2A self-cleaving peptide, was targeted to the *p53* locus (Figures 2A and S4) (Trichas et al., 2008). This design allowed us to track individual *p53^∗^*^*/wt*^ cells in a *p53*^*wt/wt*^ background. We confirmed that the p53^∗^ protein had properties similar to those of the human p53^R248W^ protein and observed perturbation of p53 targets and differentiation genes in cultured keratinocytes consistent with previous reports (Freije et al., 2014, Song et al., 2007, Truong et al., 2006) (Figures S4 and S5). Following tag cleavage, a 20-amino-acid peptide remained at the C terminus of p53^∗^ protein. To test whether this peptide altered the properties of the p53^∗^ protein, we generated a second conditional mouse strain with an untagged *p53*^*R245W*^ mutant allele. RNA sequencing (RNA-seq) analysis of recombined primary cultures of *p53^∗^*^*/*^*^∗^* and the untagged *p53*^*R245W/R245W*^ keratinocytes revealed minimal differences in transcription (Figure S5J; Table S1).

### *p53*^*∗/wt*^ Mutant Progenitor Cells Colonize Wild-Type Epidermis

**Figure 3:**
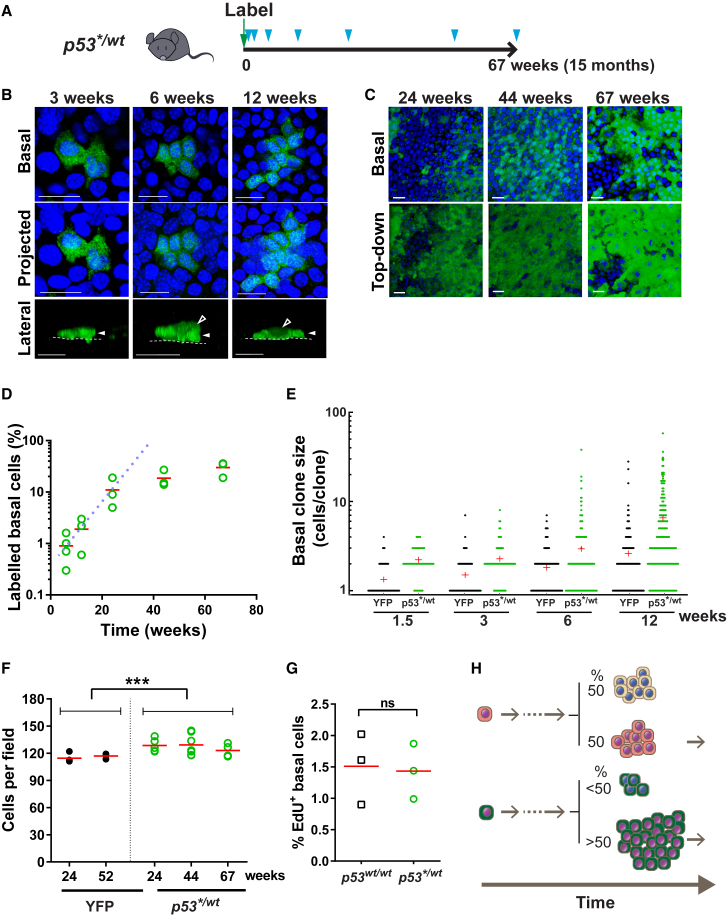
Heterozygous *p53*^*R245W*^ (*p53*^*∗/wt*^) Mutant Cell Fate (A) Protocol: clonal labeling of *Ahcre*^*ERT*^*p53*^*flR245W/wt*^ (*p53*^*∗/wt*^) mice followed by sampling (triangles). (B) Rendered confocal z stacks showing typical *p53*^*∗/wt*^ clones in back skin epidermis. Basal, top-down view of basal layer; projected, top-down view through all nucleated cell layers; lateral, side view. Cells in basal and first suprabasal cell layers are indicated by closed and open arrowheads, respectively; dotted line, basement membrane. Green, GFP; blue, DAPI. Scale bars, 20 μm. (C) Rendered z stacks showing basal and top-down views of typical whole mounts at times indicated. Green, GFP; blue, DAPI. Scale bars, 20 μm. (D) Proportion of labeled basal cells at indicated time points. Averaged value from 4–5 fields per animal, n = 3 animals per time point except n = 4 animals at the 6-week time point. Red lines, mean value. Dotted line indicates the expected growth of *p53*^*∗/wt*^ basal cells clone area if the rate of expansion is constant. (E) Clone size distributions (basal cells per clone). Red cross indicates mean clone size. Data are from 3–5 mice per time point. For *p53*^*∗/wt*^ and *RYFP*, respectively, n = 50, 93 clones at 1.5 weeks, 75, 106 clones at 3 weeks, 199, 181 clones at 6 weeks and 196, 183 clones at 12 weeks. (F) Mean basal cell density in *p53*^*∗/wt*^ and *RYFP* control mice. Data are means of 5 fields per mouse. n ≥ 4 mice at each time point/group. Exact sample numbers are in corresponding supplementary table, ^∗∗∗^p = 0.0007 by Mann-Whitney test. (G) Average percentage of EdU-labeled basal cells in *p53*^*∗/wt*^ clones at the 12-week time point compared to non-labeled cells (*p53*^*wt/wt*^) in same mouse. n = 3 mice. ns, no significant difference by paired t test. (H) Schematic illustration of *p53*^*∗/wt*^ cell behavior. A bias in the fate of *p53*^*∗/wt*^ cell can result in an increase in the proportion of *p53*^*∗/wt*^ progenitors in the IFE, even if the rate of mutant cell division was the same as that of wild-type cells. See also Figure S1–S6 and Tables S2 and S7.

To track the fate of *p53*^*∗/wt*^ progenitors *in vivo*, we bred *Ahcre*^*ERT*^*p53*^*∗/wt*^ mice and induced recombination in individual cells in adult mice (2B and Figures 3A). *p53*^*∗/wt*^-expressing clones in IFE whole mounts were imaged by confocal microscopy (Figures 3B and 3C). In contrast to control R-YFP mice, in which the area occupied by YFP^+^ basal cells remained approximately constant after induction, the proportion of *p53*^*∗/wt*^ basal cells rose progressively indicating the mutant population had a competitive advantage over their wild-type neighbors (Figures 3D and S2D). The number of basal cells/clone was consistently higher in *p53*^*∗/wt*^ than in YFP^+^, *p53*^*wt/wt*^ clones at the same time point (p < 0.0001 at 1.5, 3, 6, and 12 weeks, two-tailed Mann-Whitney test, Figure 3E). By 24 weeks, the *p53*^*∗/wt*^ clones had expanded so much that they had fused, but we noted a modest but statistically significant increase in the basal cell density (cells/area, p = 0.0007 by two-tailed Mann-Whitney test) (Figures 3C, 3D, and 3F). We concluded that following induction *p53*^*∗/wt*^ cells are dominant over wild-type keratinocytes, leading to colonization of the IFE.

Next, we investigated the cellular mechanism(s) that underpinned the competitive advantage of *p53*^*∗/wt*^ progenitors. Heightened resistance to the canonical *p53* stress responses in the mutant cells seemed unlikely, as apoptosis was hardly detectable (0%–0.5%) and growth arrested/senescent wild-type keratinocytes were not detected in wild-type epidermis (Figure S2J; Table S2) (Clayton et al., 2007, Doupé et al., 2010). The proportion of mutant and wild-type basal cells staining for 5-ethynyl-2′-deoxyuridine (EdU), a marker of the S phase of the cell cycle, was similar, arguing that the cell-cycle time and the proportion of progenitors were not substantially altered by the mutation (Figure 3G). Within the single-progenitor paradigm, a further candidate explanation for the competitive advantage of *p53*^*∗/wt*^ progenitor cells was a statistical bias in the fate of mutant progenitors, leading to an excess of dividing over differentiating daughters per average cell division. Such a bias would result in an increase in the proportion of *p53*^*∗/wt*^ progenitors in the IFE at each successive round of cell division, even if the rate of mutant cell division was the same as that of wild-type cells (Figure 3H). Quantitative analysis showed that up to 3 months post induction the observed behavior of *p53*^*∗/wt*^ clones was indeed consistent with such a model, with a marked excess of divisions generating two progenitor daughters over two differentiating daughters (Figures S6A and S6B; STAR Methods).

Strikingly, beyond 24 weeks post induction, the rate of expansion of the *p53*^*∗/wt*^ population slowed substantially (Figure 3D). Yet, EdU data at later time points suggest that this was not due to a decrease in the cell-division rate (Table S3). This argues that the cell-fate imbalance of mutant progenitors is not cell autonomous but responds to changes in the cellular environment, such as the observed increase in basal cell density by 24 weeks (Figure 3F) (Miroshnikova et al., 2018, Roshan et al., 2016). Also, since the proportion of mutant cells adjacent to wild-type cells will decrease as the area of mutant epidermis rises, any imbalance in fate driven by competition between mutant and wild-type will inevitably decline over time. The lack of long-term clonal level data precluded further analysis to resolve between these mechanisms. However, it is clear that the reversion of *p53*^*∗/wt*^ cell dynamics toward normal contributed to the ability of normal tissue to tolerate mutant cells.

### *p53*^*∗/wt*^ Perturbs Keratinocyte Differentiation and Shedding

**Figure 4:**
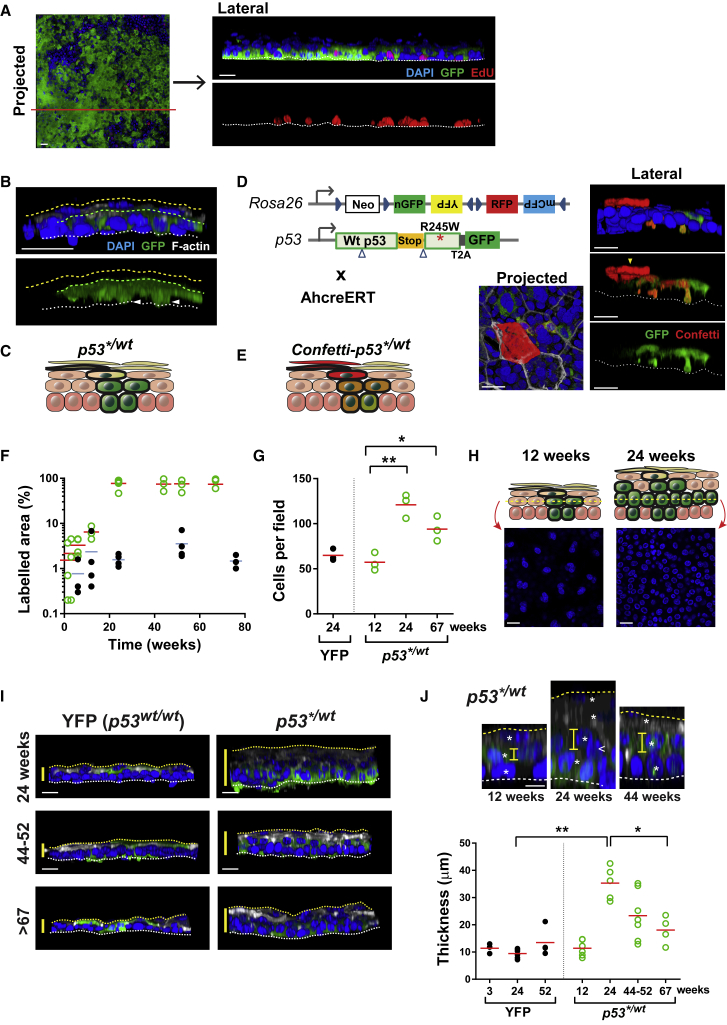
Behavior of Differentiating *p53*^*∗/wt*^ Keratinocytes (A) Rendered confocal z stacks showing *p53*^*∗/wt*^ clone area. Lateral view shows the section of projected image (red line). White line, basement membrane; blue, DAPI; green, GFP; red, EdU. Scale bars, 20 μm. (B) Side view of *p53*^*∗/wt*^ clone at the 12-week time point. White line, basement membrane; yellow line, epidermal surface; green line, outer limit of GFP-expressing cells. Green, GFP; blue, DAPI; white, F-actin. Scale bar, 20 μm, arrowheads, basal layer cells. (C) Schematic: typical *p53*^*∗/wt*^ clone with GFP expression (green) confined to basal and first suprabasal cell layers. (D) Multicolor lineage tracing in *Ahcre*^*ERT*^*Rosa26*^*flConfetti/wt*^*p53*^*∗/wt*^ animals. Left: the multicolor confetti reporter allele encodes four fluorescent proteins. Different *cre-*mediated inversion and excision recombination events result in the heritable expression of one of the four fluorescent proteins depicted, nuclear GFP, yellow fluorescent protein (YFP) red fluorescent protein (RFP), or membrane cyan fluorescent protein (CFP). The fate of *p53* mutant progenitors can be tracked in double-labeled clones even if the *p53* locus becomes inactive in the differentiating progeny. Right: rendered z stacks of typical *Confetti-p53*^*∗/wt*^ double-labeled clone 12 weeks post induction of *Ahcre*^*ERT*^*Rosa26*^*Confetti*^*p53*^*∗/wt*^ mice. Images representative of 8 clones. Yellow arrowhead, cornified cell. Scale bars, 20 μm. (E) Schematic: RFP expression reveals differentiation of *p53*^*∗/wt*^ clone. (F) Projected area of labeled epidermis (%) in induced *p53*^*∗/wt*^ (open green circles) and *p53*^*wt/wt*^*RYFP* (solid black circles) mice. Data are from 5 fields per animal. n = 3–5 mice per time point/group. Red and blue lines, mean area for *p53*^*∗/wt*^ and *p53*^*wt/wt*^*RYFP*, respectively. (G) Mean cell density in immediate suprabasal layer. Data are means of 5 fields per mouse. n = 3 mice at each time point/group except 4 fields from 1 animal are shown for YFP. ^∗^p = 0.0236, ^∗∗^p = 0.0035 by two-tailed unpaired t test. (H) Single z-slice image of immediate suprabasal layer from *p53*^*∗/wt*^ mice at indicated time point. Schematic: typical *p53*^*∗/wt*^ colonized area at indicated time point. Scale bar, 20 μm. (I) Rendered confocal z stacks showing side view of epidermal whole mounts. Yellow bars indicate the thickness of epidermis. Green, GFP; blue, DAPI; white, F-actin. Scale bar, 20 μm. White line, basement membrane; yellow line, epidermal surface. (J) Detail analysis of change in epidermal thickness. Top, confocal images (side view) showing changes in *p53*^*∗/wt*^ epidermis over time. Yellow bars, length of cells in first suprabasal layer; asterisks, each cell layers. Arrowhead indicates cell in between layers. Scale bar, 20 μm. Bottom, mean thickness of epidermis at time points indicated. n ≥ 3 mice at each time point/group. ^∗^p = 0.016, ^∗∗^p = 0.0043 by Mann-Whitney test. See also Figure S6 and Table S2–S4 and S7.

Motivated by previous reports linking *p53* with keratinocyte differentiation, we investigated the behavior of differentiating *p53*^*∗/wt*^ cells (Freije et al., 2014, Truong et al., 2006). As in wild-type IFE, mutant cell proliferation was confined to the basal layer (Figure 4A). 3D imaging of *p53*^*∗/wt*^ clones revealed that GFP expression was restricted to the basal and first suprabasal cell layers. This argued that mutant *p53* was not transcribed above the lowest epidermal cell layers and/or that *p53*^*∗/wt*^ cells failed to complete the differentiation program and reach the upper layers of the epidermis (Figures 4B and 4C). To resolve between these possibilities, we generated *Ahcre*^*ERT*^*p53*^*∗/wt*^*Rosa26*^*Confetti/wt*^ mice carrying a conditional multicolor “Confetti” allele targeted to the Rosa26 locus (Figure 4D) (Snippert et al., 2010). Imaging of clones expressing both red fluorescent protein and GFPs revealed *p53*^*∗/wt*^ cells were indeed capable of terminal differentiation into cornified layer cells (Figures 4D and 4E).

Defects in keratinocyte differentiation may have a major impact on epidermal function and promote carcinogenesis, motivating us to examine the behavior of differentiating mutant cells more closely (Darido et al., 2016). Over the first 6 months after induction, the area occupied by GFP-expressing mutant suprabasal cells rose even faster than the area occupied by mutant basal cells, arguing differentiating mutant cells accumulate in the tissue compared with their wild-type counterparts (Figures 4F and 3D). In keeping with this hypothesis, the thickness of the IFE and the density of cells in the first suprabasal layer increased substantially associated with changes in cell morphology, signs of cellular disorganization and extra cell layer(s) (Figures 4G–4J and S6C).

In order to explore possible mechanisms that could lead to this scenario, we implemented computational simulations of the cell-population dynamics and tissue structure under various hypotheses incorporating parameters measured from the epidermis (STAR Methods). Briefly, we considered changes in either the proportion of symmetric divisions, stratification rate, or shedding rate along with the extent of cell-fate imbalance. Predicted changes in the proportion of *p53*^*∗/wt*^ cells in basal/suprabasal layers and tissue thickness were compared against experimental observations. Only *p53*^*∗/wt*^ progenitor cell-fate imbalance accompanied by a substantial reduction in the shedding rate was able to reproduce the patterns seen, arguing this was the most likely explanation (Figure S6D; STAR Methods). Strikingly, however, beyond 6 months, the area occupied by mutant suprabasal cells remained approximately constant while epidermal thickness decreased, arguing that mutant cell differentiation and shedding had been at least partially restored. This slow adaptation of mutant differentiating cell dynamics resulted in the preservation of tissue integrity without the development of epidermal tumors (Table S4).

**Figure 5:**
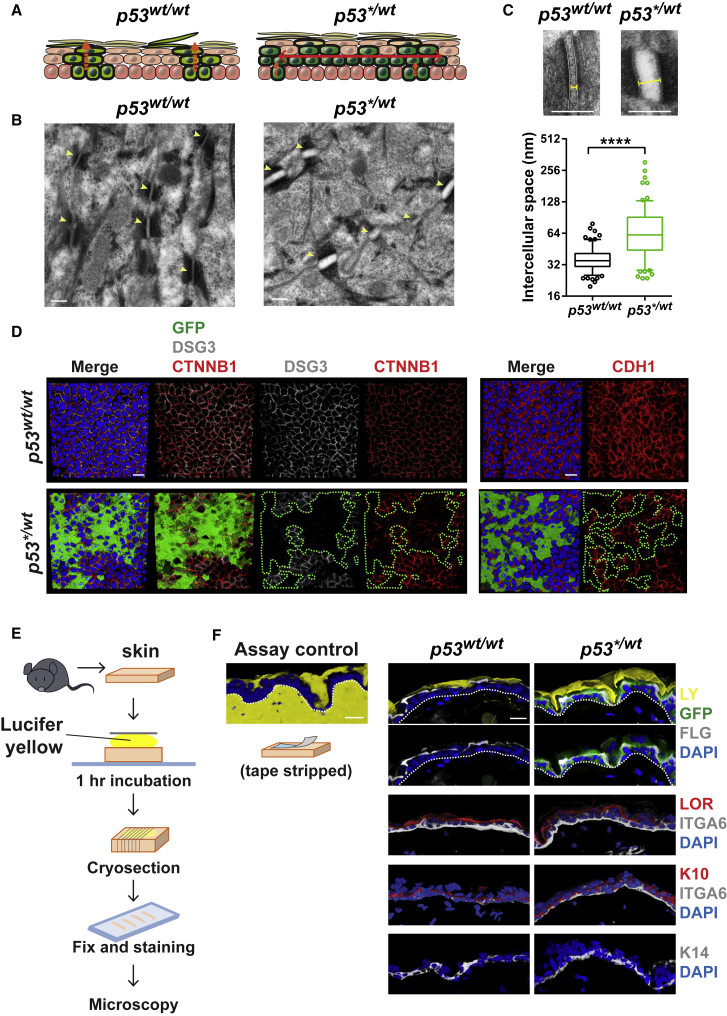
Disrupted Cell Adhesion in *p53*^*∗/wt*^ Epidermis at 1 Year post Induction (A) Schematic: movement of differentiating cells is predominantly vertical in *p53*^*wt/wt*^ mice but the excess of suprabasal over basal cells in *p53*^*∗/wt*^ IFE argues there is extensive lateral displacement of mutant cells. (B–F) Analysis of epidermis colonized by *p53*^*∗/wt*^ at 1 year post induction. Uninduced mice were used as control. n = 3 mice per group. (B) Transmission electron microscopy of induced *p53*^*∗/wt*^ and uninduced *p53*^*wt/wt*^ IFE. Arrows indicate desmosomes. Scale bar, 200 nm. (C) Intercellular distance (yellow capped bar) at desmosomes in *p53*^*wt/wt*^ (black) and *p53*^*∗/wt*^ (green) IFE. Box plots show median (line across box), 25^th^ and 75^th^ percentiles (box) and 5^th^ and 95^th^ percentiles (whiskers), dots are outliers (dots). n = 150 desmosomes from 3 animals per group. Scale bar, 200 nm. ^∗∗∗∗^p < 0.0001 by Mann-Whitney test. (D) Bottom-up view of rendered confocal z stacks showing the expression of cell adhesion molecules in *p53*^*∗/wt*^ and *p53*^*wt/wt*^ IFE. Images are representative from 3 mice per genotype. White, DSG3; red, CTNNB1 (left panels), CDH1 (right panels); green, GFP; blue, DAPI. Green line, *p53*^*∗/wt*^ clone area. Scale bar, 20 μm. (E) Scheme of epidermal permeability barrier function assay. (F) Cryosections of *p53*^*∗/wt*^ epidermis showing normal expression of markers and barrier function compared to that of *p53*^*wt/wt*^. *p53*^*∗/wt*^ colonized over 70% at this time point and GFP (green) indicates the expression of *p53^∗^* transcript. Lucifer yellow is excluded by a normal competent epidermal surface barrier; in the assay control, barrier was removed by tape stripping. Yellow, lucifer yellow; blue, DAPI. FLG, filaggrin; LOR, loricrin; ITGA6, integrin α6; K10, keratin 10; K14, keratin 14. Scale bar, 20 μm. See also Table S7.

The mismatch between the proportion of mutant basal and suprabasal cells has implications for cell movement within the epidermis. In normal IFE, the majority of differentiating cells stratify vertically through the suprabasal cell layers (Figure 5A) (Doupé et al., 2010, Rompolas et al., 2016). In contrast, in *p53*^*∗/wt*^ IFE, most mutant suprabasal cells overlie wild-type basal cells, indicating extensive lateral displacement has occurred (Figure 5A). We hypothesized that there may be alterations in cell-cell adhesion permissive of lateral migration in mutant IFE. Transmission electron microscopy of *p53*^*∗/wt*^ IFE at a year post induction revealed widespread disruption of desmosomes (Figures 5B and 5C) (Dusek and Attardi, 2011). Expression of the desmosomal proteins, DSG3 and CDH1, and levels of membrane-associated CTNNB1, which binds CDH1, were diminished in *p53*^*∗/wt*^ compared with adjacent wild-type areas (Figure 5D). Despite these changes, the *p53*^*∗/wt*^ epidermis retained its “outside-in” barrier function (Figures 5E and 5F). We concluded that the lateral migration of *p53*^*∗/wt*^ suprabasal cells may be facilitated by the disruption of desmosomes.

### Low-Dose UV Light Exposure Drives Mutant Clone Expansion

Human epidermis is frequently exposed to low doses of UV light, below the level that causes keratinocyte apoptosis and sunburn (Jonason et al., 1996). As well as generating new mutations, repeated low doses of UV have been argued to drive the expansion of clones carrying p53 protein stabilizing mutations (Klein et al., 2010). We therefore investigated the effect of such physiological exposure.

**Figure 6:**
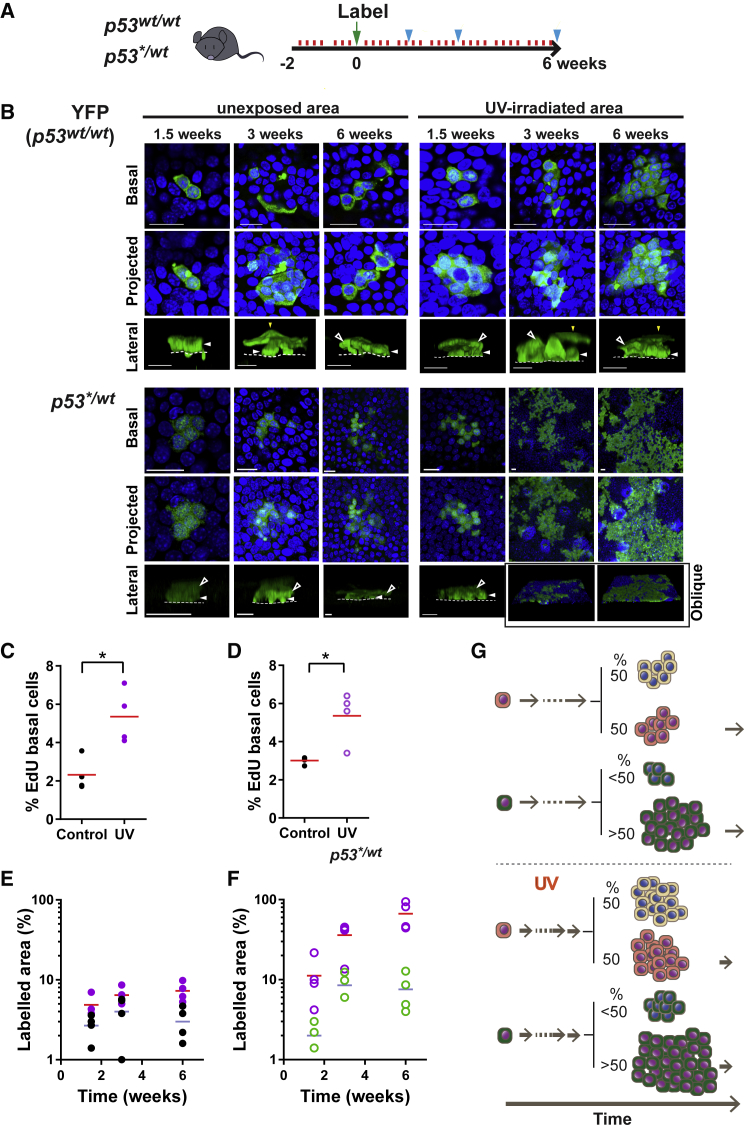
Short-Term Low-Dose UV Exposure Accelerates *p53*^*∗/wt*^ Colonization (A) Protocol: *AhcreERT-p53*^*∗/wt*^ or -*RYFP* mice were exposed to a sub-erythema dose of UVB daily, 4 days per week for 2 weeks (red lines), after which labeling was induced (green arrow) and UV exposure continued; sample collection is indicated by blue arrows. (B) Rendered confocal z stacks showing representative clones in epidermal whole mounts. Green indicates YFP in *YFP* panels and GFP in *p53*^*∗/wt*^ panels; blue, DAPI. Scale bars, 20 μm. Basal, immediate suprabasal cells and basement membrane are indicated by closed and open arrows and dotted line, respectively. (C and D) Average percentage of EdU-labeled basal cells at the 6-week time point in induced *Ah*^*creERT*^*-p53*^*∗/wt*^ (D) or *Ah*^*creERT*^-*RYFP* (C) IFE. Samples were taken from UV-irradiated (purple circles) or adjacent unexposed areas (ctl, black circles). Values are mean from 5 fields per mouse. Red line indicates mean. n = 4 mice per condition. ^∗^p < 0.05 by paired t test. Percentage EdU in UV-irradiated epidermis in (D) was quantified in *p53*^*∗/wt*^ clone area. (E and F) Projected area of labeled UV irradiated and adjacent unirradiated IFE. Values are percentage from 6 fields. n = 4 mice per time point. (E) *RYFP* (purple, UV; black, unirradiated). (F) *p53*^*∗/wt*^ (purple, UV; green, unirradiated). (G) Schematic illustration of the effect of UV on clone behavior. UV increases the rate of cell division in both *p53*^*wt/wt*^ and *p53*^*∗/wt*^ cells but accelerates IFE colonization by mutant cells as *p53*^*∗/wt*^ progenitors retain a bias in fate, generating more progenitor than differentiating daughters. See also Figure S7 and Tables S5 and S7.

First, we examined the impact of a short course of a low level UV-B (sub minimal erythema dose, MED, wavelengths 280–315 nm) treatment on *p53*^*wt/wt*^ and *p53*^*∗/wt*^ progenitors *in vivo*. The UV dose was titrated to induce detectable DNA damage with minimal change in apoptosis (Figures S7A and S7B; Table S5). An area of shaved dorsal epidermis was exposed to daily doses of UV for 4 days per week. After 2 weeks, *cre* was induced and samples collected from irradiated and adjacent unexposed epidermis for up to 6 weeks (Figures 6A and 6B). In wild-type *p53*, R-YFP mice, the proportion of EdU^+^ basal cells and the mean number of cells per clone were increased in irradiated compared to unexposed epidermis, consistent with UV treatment accelerating the rate of cell division (Figures 6C, S7C, and S7D). However, the area of labeled epidermis remained approximately constant despite the increased rate of epidermal turnover (Figure 6E). We concluded that the progenitor population continued to maintain the epidermis with balanced production of progenitor and differentiating keratinocytes during low-dose UV irradiation.

In induced *p53^∗^*^*/wt*^ animals, UV treatment greatly accelerated IFE colonization by mutant cells (Figures 6B and 6F). However, the proportion of proliferating EdU^+^ *p53^∗^*^*/wt*^ basal cells in irradiated IFE was similar to that in irradiated wild-type cells (Figures 6C and 6D). As in the unexposed epidermis, these observations indicate the fate of *p53^∗^*^*/wt*^ progenitors is biased toward proliferation. Accelerated turnover along with a fate imbalance is predicted to lead to a faster *p53^∗^*^*/wt*^ colonization (Figure 6G; STAR Methods).

**Figure 7:**
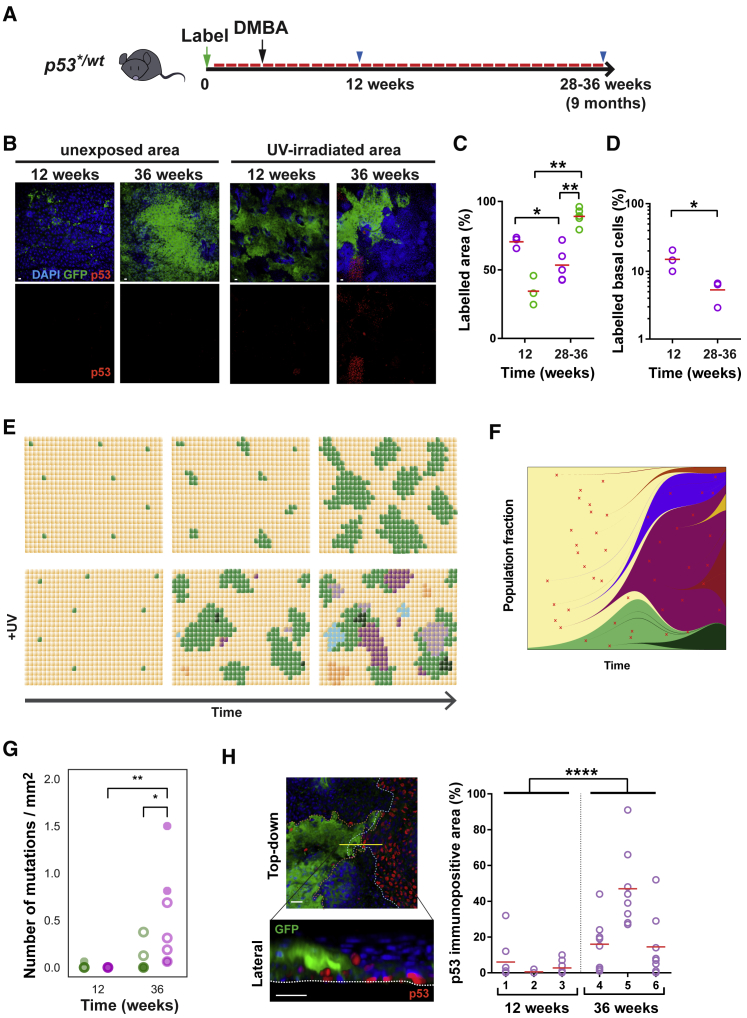
Long-Term UV Exposure Depletes Epidermis of *p53*^*∗/wt*^ Cells (A) Protocol: *Ahcre*^*ERT*^*-p53*^*∗/wt*^ mice were induced (green arrow), treated with a single dose of dimethylbenzanthracine (DMBA, black arrow), followed by repetitive sub-minimal erythema doses of UVB (red bars); blue arrows indicate sampling. (B) Confocal z stacks showing projected views of UV-irradiated and an adjacent unexposed area of IFE. Blue, DAPI; green, GFP, reporting *p53^∗^* transcription; red, p53 indicating a p53 protein-stabilizing mutation. Scale bar, 20 μm. (C) Labeled projected area of *p53*^*∗/wt*^ IFE in UV-exposed (purple circles; red line indicates mean value) and adjacent unexposed areas (green circles; red lines indicate mean value). n = 3 at the 12-week time point and n = 5 at the 28- to 36-week time point. Comparison between different time points, within same animal (unexposed versus UV irradiate area), ^∗∗^p = 0.0046, ^∗^p = 0.036 by unpaired two-tailed t test, ^∗∗^p = 0.0071 by paired t test. (D) Proportion of labeled basal cells in UV-irradiated IFE at indicated time points. Values are mean percentage from 5–8 fields per animal. n = 3 mice per time point. Red bars, mean value. ^∗^p = 0.042 by unpaired two-tailed t test. (E) Hypothesis: effect of prolonged low-dose UV on *p53*^*∗/wt*^ clonal dynamics. Following induction, in the absence of UV, *p53*^*∗/wt*^ clones (green) expand progressively in a background of wild-type cells (beige). In UV-exposed IFE, a wide variety of different mutant clones arises, indicated by multiple colors, some of which may expand, outcompete, and displace *p53*^*∗/wt*^ cells from the IFE. (F) Simulation of clone competition under ongoing mutagenesis (see Video S1). A transgenic mutant (green) is induced at 1% frequency in a background of wild-type cells (yellow). Subsequently, new mutations (red cross, shades of green if in transgenic cells, other colors if in wild-type cells) are induced at random and assigned a fitness value as described in STAR Methods. (G) The number of mutations detected per square millimeter by the Shearwater algorithm in each 16-mm^2^ biopsy of mouse back skin: 3 induced and 2 non-induced mice at 12 weeks, 4 induced and 3 non-induced mice at 36 weeks. Solid circles, non-induced samples; open circles, induced samples. Green, non-exposed skin; purple, UV irradiated. ^∗∗^p < 0.01, ^∗^p = 0.02 by two-tailed Mann-Whitney test. Note that this method does not detect the induced and uninduced *p53^∗^* allele. (H) Confocal z stacks showing *p53*^*∗/wt*^ clone in direct contact with p53-immunopositive clone in UV-irradiated area (36 weeks post-induction). Blue, DAPI; green, GFP; red, p53. Scale bar, 20 μm. Dashed lines indicate outline of clones in top-down image and basement membrane in lateral view. Graph on the right shows proportion of p53 immunopositive area in UV-irradiated skin. Measurement of 8 fields of view per mouse are plotted individually. n = 3 mice per time point. Red lines indicate mean values. ^∗∗∗∗^p < 0.0001 by two-tailed Mann-Whitney test. See also Figure S7 and Tables S6 and S7.

In humans, low-dose UV exposure continues over decades and results in a dense patchwork of epidermal clones carrying mutations in *p53* and other genes (Martincorena et al., 2015). To model this scenario, *p53*^*∗/wt*^ mice were induced and the skin given a single treatment with the mutagen dimethylbenzanthracene (DMBA) to load the epidermis with mutations (Abel et al., 2009). Dorsal epidermis was then treated with sub MED UV for up to 9 months, and exposed and adjacent unexposed areas compared (Figure 7A). Over the first 12 weeks the area occupied by GFP-labeled *p53^∗^*^*/wt*^ cells expanded significantly faster in the presence of UV light, as shown above (Figures 7B and 7C). However, continuing exposure resulted in a progressive decline in the *p53^∗^*^*/wt*^ population (Figures 7B–7D).

Given the short-term effect of UV treatment, the depletion of *p53*^*∗/wt*^ cells during long-term exposure was surprising. Several different scenarios can be envisaged to explain this observation. First, it might result from a non-cell-autonomous adaptation to a local tissue stress, e.g., such as sensitivity to increased cell density, which would also explain the restraining of *p53*^*∗/wt*^ expansion in non-irradiated epidermis. Alternatively, cell-autonomous factors, such as long-term UV-induced accumulation of cellular damage in p53^∗/wt^ cells or an adaptive increase in wild-type cell fitness may cause a decline in mutant competitiveness. Another possibility is competition between *p53*^*∗/wt*^ cells and mutations caused by the DMBA treatment or UV irradiation. In the last scenario, UV exposure generates *new* mutations with a range of possible effects on competitive fitness of mutant progenitors. Initially, *p53*^*∗/wt*^ cells encounter mostly wild-type cells over which they are dominant, resulting in progressive expansion of the *p53*^*∗/wt*^ population. However, as the experiment proceeds, DMBA- or UV-induced mutant clones fitter than wild-type cells will expand and eventually collide with each other and with *p53^∗^*^*/wt*^ cells. At this point, the less-fit cells would be displaced from the tissue (Figure 7E). Simple simulations embodying this hypothesis suggest such competition as a possible explanation for the observed changes in the *p53^∗^*^*/wt*^ population (Figures 7F and S7E; STAR Methods; Video S1). Hence, long-term UV exposure may contribute to the decrease in the *p53^∗^*^*/wt*^ population both by inducing mutations that allow cells to out-compete *p53^∗^*^*/wt*^ and by providing an environment in which other mutations have an advantage over *p53^∗^*^*/wt*^.

Video S1. Simulation of Clone Competition under Ongoing Mutagenesis A visualization of the non-spatial simulation shown in Figure 7F. A transgenic mutant (green) is induced at 1% frequency in a background of wild-type cells (yellow). Subsequently, new mutations (shades of green if in transgenic cells, other colors if in wild-type cells) are induced at random and assigned a fitness value as described in the Supplemental Information.

The cell-competition hypothesis in particular makes several testable predictions. If competition is responsible for the depletion of *p53*^*∗/wt*^ cells after 36 weeks, we would expect that clones carrying protein-altering mutations (possibly with the UV mutational signature) would be present in exposed epidermis and that the number and size of such clones would increase from 12 to 36 weeks of UV treatment. To test this, we used ultra-deep targeted exome sequencing on a panel of 74 genes linked with murine squamous carcinogenesis. Both induced and non-induced mice were included to check that the observed mutations were not merely passenger mutations within *p53*^*∗/wt*^ clones.

While no mutations were detected in epidermis exposed to UV for 12 weeks, we identified 58 mutations in epidermis exposed to UV for 36 weeks compared with 8 in adjacent unexposed skin in 7 mice (p = 0.02, 2-tailed Mann-Whitney test) (Figures 7G and S7F; Table S6). These mutant calls correspond with very large clones as the lower limit of detection was ∼0.15 mm^2^ in area. The mutations were largely C > T nucleotide changes, consistent with the UV-light mutational signature, and the majority of mutations were non-synonymous, making it feasible for some of them to have altered cellular competitive fitness (Figures S7G and S7H). We noted recurrent cancer-associated mutations in *Trp53*, finding *R270C* mutants in UV-exposed epidermis in 4/7 mice after 36 weeks UV. The mutations found in the non-induced samples show that the expansion of new mutations does not rely on hitch-hiking within *p53*^*∗/wt*^ clones. Taken together, these observations are consistent with clones carrying UV- or DMBA- induced mutations displacing *p53*^*∗/wt*^ cells during prolonged UV exposure. In contrast, in the adjacent unexposed areas of the skin, the expansion of the *p53*^*∗/wt*^ population was not impeded by competition (Figures 7B and 7C).

A further prediction of the cell-competition hypothesis is that transgenic and UV-induced mutant clones must ultimately collide. One way to visualize such events is to immuno-stain for p53. This reveals cells carrying UV-induced, protein-stabilizing mutations, such as *Trp53*^*R270C*^, but does not detect the low levels of wild-type p53 or mutations that do not stabilize the protein including p53^∗^ (Figure S5H) (Jonason et al., 1996). We observed an increase in the areas covered by p53 immuno-positive cells from 12 to 36 weeks under UV exposure but barely detected any in adjacent un-irradiated epidermis at 36 weeks (Figures 7B and 7H). In several cases, these cells were in direct contact with *p53*^*∗/wt*^ regions, infiltrating underneath differentiating edges of *p53*^*∗/wt*^ clones (Figure 7H). We concluded that cells carrying protein-stabilizing mutations in *p53* do indeed collide with *p53*^*∗/wt*^ cells during prolonged UV exposure.

Altogether, these results suggest that, in addition to the phenotypic adaptation observed in the non-irradiated experiments, competition between mutant clones may be an important factor in constraining *p53* mutant clone expansion in the evolving mutational landscape of normal epidermis

## Discussion

Here, we show that the phenotype of epidermal progenitor cells carrying a heterozygous *p53* gain-of-function mutation adapts to alterations in the cellular environment. In mice protected from UV light, *p53^∗^* confers a strong competitive advantage over wild-type progenitor cells, as the average mutant cell division generates more dividing than differentiating progeny. In addition, retardation of terminal differentiation and shedding leads to mutant cell accumulation in the suprabasal layers. Unchecked, the combination of progenitor cell fate favoring proliferation and a decrease in cell shedding would be sufficient to generate tumors (Frede et al., 2016). However, the progressive attenuation of the mutant phenotype enables the epidermis to retain its functional integrity despite a high burden of *p53* mutant cells.

The mechanisms underpinning the adaptation of *p53*^*∗/wt*^ cells remain to be determined. The slowing of expansion of the *p53*^*∗/wt*^ basal cell population beyond 6 months post induction is accompanied by crowding of cells in the basal layer. A similar phenomenon has been observed in “imprisoned” UV-induced *p53* mutant clones and *Notch* mutant keratinocytes (Alcolea et al., 2014, Zhang et al., 2001). Keratinocyte differentiation is sensitive to mechanical forces suggesting that basal cell crowding may promote a return toward cellular homeostasis (Le et al., 2016, Roshan et al., 2016). New technologies will be required to determine the possible role of feedback effects from suprabasal cell crowding on epidermal proliferation and tissue turnover.

A persistent legacy of *p53*^*∗/wt*^ mutation is the loss of desmosomes and downregulation of expression of CDH1 and other adhesion proteins, consistent with the observation that the human *p53*^*R248W*^ mutant represses the expression of CDH1 *in vitro* (Dusek and Attardi, 2011, Wang et al., 2009). However, within the mixed population of mutant and wild-type cells the tissue does not exhibit a barrier or fragility defect.

In non-UV-exposed epidermis, the early competitive dominance of the *p53*^*∗/wt*^ mutant is explicable in terms of cell-autonomous effects on progenitors. The short-term effects of low-dose UV light exposure may also be explained within this paradigm. UV irradiation increases the rate of cell division of both wild-type and mutant progenitors, but the imbalance of cell fate in mutant cells remains. The result is a substantial acceleration of epidermal colonization by *p53*^*∗/wt*^ cells.

DMBA/UV exposure leads to a complex and heterogeneous mutational landscape. After long-term UV exposure, the *p53*^*∗/wt*^ population declines but is not entirely displaced. We hypothesize that individual clone fates would then depend on the chance acquisition of additional mutations and the competitive fitness of neighboring mutant clones. It remains to be seen whether competitive interactions promote, inhibit, or are neutral in carcinogenesis. Properties that allow a particular mutant to survive in early clonal competition could differ from those required for outgrowth of neoplastic cell population (Aktipis et al., 2013). The stepwise acquisition of further mutations may then be key for breaking these evolutionary bottlenecks (Nowell, 1976).

This study argues that the patchwork of clones carrying oncogenic mutations in sun-exposed human epidermis is shaped by both phenotypic adaptation and cell competition. Understanding how mutant progenitor clones interact is key to understanding not only epidermal physiology, but also the formulation of rational approaches to prevent malignant transformation.

## STAR★Methods

### Key Resources Table

**Table:** 

REAGENT or RESOURCE	SOURCE	IDENTIFIER
**Antibodies**		

Caspase 3 (CAS3)	Abcam	Cat# ab44976; PRID: AB_868674
Caspase 3 (CAS3)	Abcam	Cat# ab2302; PRID: AB_302962
Green fluorescent	Life technologies	Cat# A10262; PRID: AB_2534023
Cyclo Butane prymidine	CosmoBio	Cat# NMDND001; PRID: AB_1962813
p53 (CM5)	Vector Laboratories	Cat# VP-P956; PRID: AB_2335917
p53 (Pab1801)	Abcam	Cat# ab26; PRID: AB_303198
Phospho Serine 392 p53	Millipore	Cat# 04-244; PRID: AB_1587353
Phospho Serine 15 p53	Cell signaling	Cat# 9284; PRID: AB_331464
Acetyl Lysine 379 p53	Cell signaling	Cat# 2570; RRID:AB_823591
Loricrin	Covance	Cat# PRB-145P; PRID: AB_292095
Filaggrin	Covance	Cat# PRB-417P; PRID: AB_291632
Cytokeratin 14	Covance	Cat# PRB-155P; PRID: AB_292096
Cytokeration 10	Abcam	Cat# ab 76318; PRID: AB_1523465
Tubulin β2	Abcam	Cat# ab151318
Mouse double minute 2	Abcam	Cat# ab16895; PRID: AB_2143534
CD45	Biolegends	Cat# 103102; RRID:AB_312967
E-cadherin (CDH1)	Cell signaling	Cat# 3195; PRID: AB_10694492
Desmoglein 3 (DSG3)	Santa Cruz Biotechnology	Cat# sc-23912; RRID:AB_627422
Beta Catenin (CTNNB1)	Cell signaling	Cat# 9562; PRID: AB_331149
Phalloidin	Life technologies	Cat# A22287; PRID: AB_2620155
Lrig1	R&D systems	Cat# AF3688; RRID:AB_2138836
Alexa Fluor 647 anti-human/mouse CD49f	BioLegend	Cat# 313610; PRID:AB_493637

**Bacterial and Virus Strains**		

Adeno-cre	Vector Laboratories	Cat# 1045

**Chemicals, Peptides, and Recombinant Proteins**		

β-napthoflavone	MP Biomedicals	Cat# 156738
Tamoxifen	Sigma	Cat# N3633
Doxycycline	Sigma	Cat# D9891
Fish Skin gelatin	Sigma Aldrich	Cat# G7765
Bovine Serum Albumin	Merk MIllipore	Cat# 126575
Donkey serum	Sigma Aldrich	Cat# D9633
Goat serum	Sigma Aldrich	Cat# G9023
Lucifer yellow	Sigma	Cat# L0259
Keratinocyte Serum Free media	Invitrogen	Cat# 10744018
Epidermal growth factor	GIBCO	Cat# 10450-013
Bovine pituitary extract	GIBCO	Cat# 13028-014
Polybrene	Sigma	Cat# H9268
0.25% trypsin	Sigma	Cat# T4424
HEPES	GIBCO	Cat# 15630080
HBSS	GIBCO	Cat#14175-053
Eagle’s Minimum Essential Medium	Lonza	Cat# LZBE06-174G
SimplyBlue Coomassie G-250	Thermo Scientific	Cat# LC6060
Protease and Phosphatase inhibitor	Thermo Scientific	Cat# 78415
10% Tris-HCl acrylamide gel	Bio-Rad	Cat# 161-155
Fibronectin	BD	Cat# 356008
Collagen Type I	BD	Cat# 354236
40, 6-diamidino-2-phenylindole (DAPI)	Sigma	Cat# D9542

**Critical Commercial Assays**		

Click-iTEdU imaging	Life technologies	Cat# C10086
Pierce TM Crosslink Magnetic IP/Co-IP kit	Thermo Scientific	Cat# 88805
RNeasy Mini kit	QIAGEN	Cat# 74106
Quantitect Reverse Transcription kit	QIAGEN	Cat# 205310
TaqMan Universal PCR Master Mix	Life technologies	Cat# 4304437
Lipofectamine 2000	Thermo Fisher	Cat# 11668-019
QuickSTART Bradford Dye reagents	BioRAD	Cat# 500-0202
Immobilon Western Chemiluminescent HRP substrate	Millipore	Cat# WBLUC0500
SuperSignal West Femto Chemiluminescent	Thermo Scientific	Cat# 34095
SimplyBlue Coomassie G-250 stain	Thermo Scientific	Cat# LC6060
PfuUltra II Fusion HS DNA polymerase	Agilent technologies	Cat# 600672
PCR purification kit	QIAGEN	Cat# 28106
Rapid DNA ligation kit	Roche	Cat# 11635379001
DNA MiniPrep kit	QIAGEN	Cat# 27106
Maxi Prep Endotoxin free kit	QIAGEN	Cat# 12362
Pierce TM Crosslink Magnetic immunoprecipitation (IP)/Co-IP kit	Thermo Scientific	Cat# 88805
QIAamp DNA Micro Kit	QIAGEN	Cat# 56304

**Deposited Data**		

RNaseq data: p53^wt/wt^	This paper	ENA: ERS1755594, ERS1755602, ERS1755610, ERS1755618
RNaseq data: p53^∗/wt^	This paper	ENA: ERS1755595, ERS1755603, ERS1755611, ERS1755619
RNaseq data: p53^∗/∗^	This paper	ENA: ERS1755596, ERS1755604, ERS1755612, ERS1755620
RNaseq data: p53^R245W/R245W^ (untagged)	This paper	ENA: ERS1755597, ERS1755605, ERS1755613, ERS1755621
Ultra-deep targeted DNA sequencing data	This paper	ENA: ERP023080

**Experimental Models: Cell Lines**		

Mouse embryonic fibroblast	Laboratory of Ashok Venkitaraman	PMID:15607980
Human derived amphotrophic phoenix cell	ATCC	ATCC CRL-3213

**Experimental Models: Organisms/Strains**		

Mouse: C57BL/6J	The Jackson Laboratory	JAX: 000664
Mouse: *Trp53*^*flR245W/flR245W*^	This paper	N/A
Mouse: *Ahcre*^*ERT*^	Kemp et al., 2004	
Mouse: *Ahcre*^*ERT*^*Trp53*^*flR245W/wt*^	This paper	N/A
Mouse: *Ahcre*^*ERT*^*R26*^*flconfetti/wt*^*Trp53*^*flR245W/wt*^	This paper	N/A
Mouse: *Ahcre*^*ERT*^*Rosa26*^*flYFP/wt*^	Clayton et al., 2007	N/A
Mouse: *R26*^*M2rTA*^*TETO-GFP*	Doupé et al., 2012	N/A

**Oligonucleotides**		

Taqman assay *Mdm2*	Life technologies	Mm01233136_m1
Taqman assay *Tubb2b*	Life technologies	Mm01620966_s1

**Recombinant DNA**		

pBabe puro IRES-EGFP	addgene	Cat# 14430
pBabe puro IRES-EGFP p53	This paper	N/A
pBabe puro IRES-EGFP p53^R245W^	This paper	N/A
pBabe puro IRES-EGFP p53^R245W^-T2A	This paper	N/A
pBabe puro IRES-EGFP p53^S389A^	This paper	N/A

**Software and Algorithms**		

LAS X	Leica	N/A
Volocity 6 and 6.3	Perkin Elmer	N/A
AxioVision	Zeiss	N/A
Tecnai User Interface	FEI	N/A
GraphPad	Prism 6	N/A
STAR 2.5.3a	Dobin et al., 2013	N/A
HTSeq framework version 0.6.1p1	Anders et al., 2015	N/A
R package: DESeq2	Love et al., 2014	https://bioconductor.org/packages/release/bioc/html/DESeq2.html
R package: pheatmap	R Kolde, R package version 61’	https://cran.r-project.org/web/packages/pheatmap/index.html
R package: RColorBrewer	ColorBrewer palettes E Neuwirth, RC Brewer - R package version, 2014 - auckland.ac.nz	https://cran.r-project.org/web/packages/RColorBrewer/index.html
R package: clusterProfiler	Yu et al., 2012	https://bioconductor.org/packages/release/bioc/html/clusterProfiler.html
R package: org.Mm.eg.db	Carlson M (2018). org.Mm.eg.db: Genome wide annotation for Mouse. R package version 3.6.0	http://bioconductor.org/packages/release/data/annotation/html/org.Mm.eg.db.html
BWA-MEM (v0.7.15)	Li, 2013	
Shearwater	Gerstung et al., 2014	https://bioconductor.org/packages/release/bioc/html/deepSNV.html
Ensembl Variant Effect Predictor (Version 84)	McLaren et al., 2016	N/A
MATLAB R2016b	MathWorks	N/A
Jupyter & Spyder 3.1 (Python 3)	Python Software Foundation	N/A

**Other**		

Leica TCS SP5 II and SP8	Leica	N/A
120kV FEI Spirit Biotwin	FEI	N/A
StepOne Plus Real-Time PCR system	Life technologies	N/A
UV irradiater UV-2	Tyler research Corporation	N/A
UV-irradiator CL-508M	Uvitec	N/A

### Contact for Reagent and Resource Sharing

Request for reagent and resource sharing should be addressed to the Lead Contact, Philip H Jones (pj3@sanger.ac.uk).

### Experimental Model and subject Details

#### Mice strains and induction of allele

All experiments were conducted according to UK government Home Office project licenses PPL22/2282 and PPL70/7543. Animals were maintained at specific and opportunistic pathogen free health status and were immune competent. No animals were involved in previous experiments and were drug naive prior to the start of experiments. Adult mice 12 weeks or more weeks in age were used for *in vivo* experiments. Animals were maintained on a *C57/Bl6* genetic background, housed in individually ventilated cages and fed on standard chow. Both male and female animals were used for experiments.

*Trp53*^*flR245W-GFP/wt*^ knock-in mice were generated by TaconicArtemis GMBH, Germany. In the targeting vector, exons 5 to 11 of the wild-type *Trp53* gene were flanked by *loxP* sites and an additional transcriptional STOP cassette inserted between the *Trp53* 3′untranslated region (UTR) and the distal *loxP* site, in order to prevent transcriptional read through to downstream exons (Figure 2A). A second *Trp53* genomic region from exon 5 to exon 11, including the splice acceptor site of intron 4 was introduced 3′ of the distal *LoxP* site. This duplicated region included a Trp53^R245W^ mutation introduced into exon 7 and a cassette including a glycine-serine-glycine flexible linker, a self-cleaving T2A peptide and the eGFP coding sequences inserted into exon 11, between the last codon of *Trp53* and the translation termination codon. Two positive selection markers were also introduced. A Neomycin resistance gene flanked by *Frt* sites was inserted 3′ of the 5′ *LoxP* site in exon 4, downstream of the predicted transcriptional initiation site. A Puromycin resistance gene flanked by F3 sites was placed downstream of the 3′UTR from the duplicated region. The targeting vector was generated using BAC clones from the *C57BL/6J RPCIB-731* BAC library and was fully sequenced. After transfection into the *C57BL/6N*^*Tac*^ embryonic stem cell line, clones that had undergone homologous recombination were selected using puromycin and neomycin and screened by Southern blotting.

The conditional *Trp53* strain was generated by *Flp*-mediated deletion of the selection markers. Prior to *cre*-mediated recombination these animals (*p53*^*∗/wt*^) express TRP53 protein from two wild-type alleles. Once the wild-type *Trp53* genomic region is deleted by *cre* both the *Trp53* mutant carrying the R245W mutation and the *eGFP* reporter are transcribed.

For lineage tracing of control, *Trp53* wild-type progenitors, the *Rosa26*^*flYFP/wt*^ (*R-YFP*) mice which express yellow fluorescent protein (YFP) from the constitutively active *Rosa 26* locus were used (Srinivas et al., 2001).

To assess the differentiation capacity of *p53*^*∗/wt*^ mutant clones, homozygous *Ahcre*^*ERT*^*R26fl*^*Confetti*^ animals were crossed onto *p53*^*∗/∗*^to create *Ahcre*^*ERT*^*R26fl*^*Confetti/wt*^*p53*^*∗/wt*^ animals (Snippert et al., 2010). Following induction this strain yields cells expressing one of 4 colors of reporter from the *Rosa26* locus and the GFP reporter of *p53* transcription. Cells doubly positive for red fluorescent protein and GFP were scored in experiments.

Each reporter line was crossed onto the *Ah*^*creERT*^ line in which transcription from a transgenic *CYP1A1* (arylhydrocarbon receptor, *Ah*) promoter is normally tightly repressed (Kemp et al., 2004). Following treatment with the non-genotoxic xenobiotic β-napthoflavone the *Ah* promoter is induced and a *cre* recombinase- mutant estrogen receptor fusion protein (creERT) is expressed. In the presence of tamoxifen, the cre^ERT^ protein enters the nucleus to mediate recombination.

For lineage tracing experiments, the relevant floxed reporter line was crossed onto the *Ah*^*creERT*^ strain. Doubly transgenic male and female animals were induced by a single interaperitoneal (i.p) injection of 80 mg/kg β-napthoflavone and 1 mg tamoxifen at 11-16 weeks of age. GFP and YFP expressing clones were visualized by immunostaining with an anti-GFP antibody.

To estimate the rate of epidermal cell division a transgenic proliferation assay was used (Doupé et al., 2012). Mice doubly transgenic for the reverse tetracycline-controlled transactivator (rtTA-M2) targeted to the *Rosa26* locus and a HIST1H2BJ-EGFP fusion protein (HGFP) expressed from a tetracycline promoter element were treated with doxycycline (DOX, 2 mg ml^-1^ in drinking water sweetened with sucrose) for 4 weeks. DOX was then withdrawn and animals were culled at different time points to track HGFP dilution.

To determine the proportion of proliferating cells, 100 μg of 5-ethynyl-2′-deoxyuridine (EdU) (Life technologies) was injected intraperitoneally 1 h before culling animals.

#### Primary culture and *in vitro* induction of allele

Primary mouse keratinocytes were isolated directly from tail epidermis of 4-12 week-old male and female *C57/BL6* wild-type, *p53*^*∗/wt*^, and *p53*^*∗/∗*^ mice and cultured in Keratinocyte Serum Free media (KSFM, Life technologies) supplemented with 1μg/ml Epidermal growth factor, 45 μg/ml Bovine pituitary extract, 20 mM HEPES-NaOH pH7.2-7.5, 1% Penicillin-Streptomycin and CaCl_2_ to give a final Ca^2+^ concentration of 0.02 mM for growth media (GM) and 0.6 mM for differentiation media (DM).

*Cre*-mediated recombination was carried out *in vitro* using an adenovirus carrying *Cre* recombinase (Adeno-*cre*, Vector Laboratories cat.no. 1045). When primary cultures reached 50%–60% confluency, cells were incubated with Adeno-*cre* at 2x10^6^ pfu/ml in GM for 18 hours at 37°C. Virus was then removed by washing in Hank’s Balanced Salt Solution (HBSS, Life technologies) three times. Cells maintained in GM for another 24 hours prior to use in experiments.

### Method Details

#### Ultraviolet (UV) irradiation

A UV irradiator UV-2 from Tyler Research Corporation was used for this study. During irradiation, animals were placed in a custom-made restrainer that restricted exposure to part of the dorsal skin. The dose of UVB-irradiation was titrated in wild-type C57BL/6 mice. A sub minimal erythema dose of UVB (750J/m^2^) was determined from the appearance of the skin, Cyclobutane Pyrimidine staining and level of cleaved Caspase 3. *Ahcre*^*ERT*^-*R-YFP* and *Ahcre*^*ERT*^*-p53*^*∗/wt*^ mice were lightly shaved on the back with electric shaver 3 days prior to the start of irradiation course, then exposed to sub MED UVB daily, 4 times a week, and shaved once a week. The irradiance was monitored by dosimeter every day. For short term treatment, mice were induced with β-napthoflavone and tamoxifen after 2 weeks of irradiation and irradiation continued as before. Animals were culled at intervals as described in the text.

UV-irradiation of cultured cells was performed with a UV irradiator (Uvitec, CL-508M with 5 × 8 W 312 nm tubes). Prior to UV-irradiation, cells were rinsed in HBSS briefly, exposed to a dose of 25 mJ/cm^2^ UVB in HBSS and then placed in growth media.

#### Wholemount sample preparation

Whole back skin was lightly shaved and treated with hair removal cream (Nair Tough Hair, Coarse/Dark). The skin was then cut into rectangular pieces of approximately 4 by 5 mm and incubated in PBS containing 5mM EDTA at 37°C for 2 hours. Samples were transferred into PBS and the epidermis was carefully scraped off using curved scalpel while holding one corner of the skin with forceps. The epidermal wholemounts were fixed in 4% paraformaldehyde in PBS for 30 minutes and then stored in PBS at 4°C.

#### Immunofluorescence

For staining, wholemounts were blocked in staining buffer (0.5% Bovine Serum Albumin, 0.25% Fish Skin Gelatin, and 0.5% Triton X-100 in PBS with 10% goat or donkey serum according to the secondary antibody used) for 1 hour at room temperature. Samples were incubated with primary antibody in staining buffer overnight, washed in PBS containing 0.2% Tween-20 four times, incubated with fluorchrome-conjugated secondary antibody for 2 hours at room temperature and washed as before After the final wash, samples were incubated with 40,6-diamidino-2-phenylindole (DAPI, 1 μg ml^-1^) in PBS at least for 20 minutes and mounted on slides using Vectashield Mounting Medium with DAPI (Vector Labs).

EdU incorporation was detected with a Click-iT imaging kit (Life technologies) according to manufacturer’s instructions.

Cryosections (20 μm thickness) were fixed in 4% paraformaldehyde in PBS for 10 minutes and stained as described above.

All immunofluorescence images are representative of at least 3 animals.

#### Imaging

Confocal images were acquired on Leica TCS SP5 II or SP8 microscopes using 10x, 20x or 40x objectives. Typical settings for acquisition of z stacks were optimal pinhole, line average 4 scan speed 400 Hz and a resolution of 1024 × 1024 pixels or 2048 × 2048 pixels. Image analysis was performed using Volocity 6 or 6.3 image processing software (Perkin Elmer).

#### Transmission Electron Microscopy

Small pieces of skin (1 mm^3^) were fixed at 20°C for 2 hours in 2% paraformaldehyde with 2.5% glutalaldehyde in 0.1 M sodium cacodylate buffer at pH 7.42 with 0.1% MgCl_2_ and 0.05% CaCl_2_. They were then rinsed three times for 10 minutes each in sodium cacodylate buffer with chlorides and placed into 1% osmium tetroxide in sodium cacodylate buffer only, at room temperature for a further 2 hours, rinsed 3 times again, mordanted with 1% tannic acid for 30 minutes and rinsed with 1% sodium sulfate for 10 minutes. The samples were then dehydrated through an ethanol series and stained *en bloc* with 2% uranyl acetate for 1 hour at the 30% ethanol stage and embedded in Epon resin. Ultrathin transverse sections were cut on a Leica UC6 microtome and mounted onto grids before contrasting with uranyl acetate and lead citrate. Finally, images were recorded on an 120kV FEI Spirit Biotwin using an F4.15 Teitz CCD camera and measurements of the junction gaps made directly using Tecnai User Interface software.

#### Clonal imaging

After immunostaining wholemounts, clones were imaged by confocal microscopy and the number of basal and suprabasal cells in each clone counted in live acquisition mode.

#### Epidermal permeability barrier function

Epidermal barrier function was assessed by Lucifer yellow (λ_ex_ 428 nm, λ_em_ 540 nm) dye diffusion assay. Mouse back skin was lightly shaved and small pieces were placed dermis side down on PBS-soaked Whatman filter paper. 10 μL of 1 mM Lucifer Yellow (Sigma L0259) in PBS was applied onto the surface of the skin and parafilm was laid on it to ensure the sample was covered with the solution. Following incubation at room temperature for 1 hour, samples were frozen in Tissue-Tek O.C.T compound (Sakura) and cyrosections (20 μm) were analyzed by confocal microscopy.

#### Immunoblotting

Cells were lysed in buffer containing 20 mM HEPES NaOH pH7.2-7.5, Glycerol 10%, 0.4 M NaCl, NP-40 0.5% (Sigma), 0.2 mM EDTA, 1 mM Dithiothreitol (DTT), and 0.01% Halt Protease and Phosphatase inhibitor (ThermoFisher Scientific, cat.no.78415) and centrifuged at 13000 rpm at 4°C for 10 minutes. Protein concentrations were measured using standard Bradford protein assays (BioRAD QuickSTART Bradford Dye Reagents, cat.no.500-0202). Lysates were mixed with equal amount of 2x loading buffer (100mM Tris-HCl pH6.8, 4% SDS, 20% Glycerol, Bromophenol blue and 0.2% β-mercaptoethanol) and boiled at 96°C for 5 minutes. 4-10 μg of each sample was loaded onto a 10% or 12% of SDS-polyacrylamide gel. Proteins were separated by electrophoresis and transferred onto Immobilon-P membrane (pore size 0.45 μm, Millipore). Membranes were incubated in blocking buffer (5% dried skimmed milk, PBS, 0.1% Tween-20) at room temperature for 1 hour and then with primary antibodies diluted in blocking buffer for 1 hour at room temperature or overnight at 4°C on rocking platform. After washing in 0.1% Tween-20 PBS three times, HRP conjugated secondary antibodies (Dakocytomation) diluted in 0.5% skimmed milk in PBST were applied to the membrane for 30 min at room temperature on a rocking platform followed by three washes in 0.1% Tween-20 PBS 20 min each. Proteins were detected using Immobilon Western Chemiluminescent HRP substrate (Millipore WBLUC0500) or SuperSignal West Femto Chemiluminescent Substrate (Thermo Scientific, cat.no. 34095) for high sensitivity.

#### Mass Spectrometry

##### Sample preparation

cDNAs encoding wild-type murine *p53*, *p53*^R245W^ and *p53*^R245W^ with a C-terminal cleaved T2A mutant sequence were amplified by PCR using cDNA from transgenic animals. A *p53*^*S387A*^ mutant was constructed by PCR mutagenesis. To produce retroviruses, pBabe puro IRES-EGFP plasmids carrying one of the above cDNAs were transfected into Amphotrophic phoenix producer cells (ATCC) using Lipofectamine 2000, according to manufacturer’s instructions (ThermoFisher). Culture media containing retrovirus was treated with polybrene (final concentration 8 μg/ml) and used to infect primary *p53*^*−/−*^ mouse embryonic fibroblasts (MEFs) (Olive et al., 2004). Whole cell lysate from the MEFs was prepared as described above, desalted using Amicon Ultra-0.5 Centrifugal Filter Unit with Ultracel-10 membrane and diluted with immunoprecipitation (IP) lysis/Wash buffer.

##### Immunoprecipitation

Approximately 4 mg of cell lysate was used for immunoprecipitation. CM5 p53 (Vector Labs Cat No #VP-P956) antibody was cross-linked with A/G magnetic beads using Pierce TM Crosslink Magnetic IP/Co-IP kit following the manufacturer’s instructions (ThermoScientific Cat No #88805). The lysate was pre-cleared and applied to 150 μl of A/G magnetic beads cross-linked with the p53 antibody. The beads were then washed and the protein was eluted by boiling at 95°C for 5 min in non-reducing buffer (2 x concentration of 100 mM Tris-HCl pH 6.8 (Sigma SLBC0806V), 4% SDS, 20% Glycerol, Bromophenol blue (BDH prolabs 101184K). The total IP product was loaded on a 10% Tris-HCl acrylamide gel (Bio-Rad Cat No #161-155) and protein bands visualized with SimplyBlue Coomassie® G-250 stain following manufacturer’s instructions (ThermoScientific Cat No #LC6060). The band at appropriate size was cut out and digested into peptides for liquid chromatography tandem*-*mass spectrometry.

##### Liquid Chromatography-Mass spectrometry/Mass spectrometry (LC-MS/MS)

Experiments were performed using a nanoAcquity UPLC (Waters Corp., Milford, MA) system and an LTQ Orbitrap Velos hybrid ion trap mass spectrometer (Thermo Scientific, Waltham, MA). Separation of peptides was performed by reverse-phase chromatography using a Waters reverse-phase nano column (BEH C18, 75 μm i.d. x 250 mm, 1.7 μm particle size) at flow rate of 300 nL/min. Peptides were initially loaded onto a pre-column (Waters UPLC Trap Symmetry C18, 180 μm i.d x 20mm, 5 μm particle size) from the nanoAcquity sample manager with 0.1% formic acid for 3 minutes at a flow rate of 10 μL/min. After this period, the column valve was switched to allow the elution of peptides from the pre-column onto the analytical column. Solvent A was water + 0.1% formic acid and solvent B was acetonitrile + 0.1% formic acid. The linear gradient employed was 3%–40% B in 60 minutes.

The LC eluent was sprayed into the mass spectrometer by means of a standard Thermo Scientific nanospray source. All *m/z* values of eluting ions were measured in the Orbitrap Velos mass analyzer, set at a resolution of 30000. Data dependent scans (Top 10) were employed to automatically isolate and generate fragment ions by collision-induced dissociation in the linear ion trap, resulting in the generation of MS/MS spectra. Ions with charge states of 2+ and above were selected for fragmentation. Post-run, the data was processed using Protein Discoverer (version 2.1., ThermoFisher). Briefly, all MS/MS data were converted to mgf files and the files were then submitted to the Mascot search algorithm (Matrix Science, London UK) and searched against a Uniprot *Mus musculus* database. A fixed modification of carbamidomethyl (C) and variable modifications of oxidation (M) and deamidation (NQ) were selected. A peptide tolerance of 10 ppm (MS) and 0.6 Da (MS/MS) were also selected along with 2 missed cleavages.

#### Transcriptome analysis

Total RNA extraction from cultured primary mouse keratinocytes was carried out using the QIAGEN RNeasy Mini kit according to manufactures instructions (QIAGEN). cDNA was synthesized using the Quantitect Reverse Transcription kit following manufactures instructions (QIAGEN) and diluted 20-foled prior to qPCR analysis.

qRT-PCR was performed on StepOne Plus Real-Time PCR system (Life technologies). Each reaction (20μl) contained 5μl cDNA, 10 μl of TaqMan® Universal PCR Master Mix (Life technologies) and 1 μl of an appropriate taqman probe. Following PCR parameters were used: hold 95°C for 10 min, followed by 45 cycles of step 1 at 94°C for 15 s, step 2 at 60°C for 55 s and acquiring to cycling A (FAM). All reactions were performed in triplicates. Data was analyzed using The StepOnePlus Real-Time PCR System and Excel.

**Table:** 

Target gene	TaqMan probe ID (Life technologies)
*Lce3d*	Mm04337274_sH
*Lor*	Mm01962650_s1
*Mdm2*	Mm01233136_m1
*Cdkn1a*	Mm04205640_g1
*Rprm*	Mm00469773_s1
*Fas*	Mm01204974_m1
*Dusp2*	Mm00839675_g1
*Tubb2b*	Mm00849948_g1

For RNA-seq, libraries were prepared in an automated fashion using an Agilent Bravo robot with a KAPA Standard mRNA-Seq Kit (KAPA BIOSYSTEMS). In house adaptors were ligated to 100-300 bp fragments of dsDNA. All the samples were then subject to 10 PCR cycles using sanger_168 tag set of primers and paired-end sequencing was performed on Illumina’s HiSeq 2500 with 75 bp read length. Reads were mapped using STAR 2.5.3a, the alignment files were sorted and duplicate-marked using Biobambam2 2.0.54, and the read summarization was done using the script htseq-count from version 0.6.1p1 of the HTSeq framework (Anders et al., 2015, Dobin et al., 2013). Differential expression analysis was done using the DESeq2 R package(Love et al., 2014), and the downstream pathway analysis and visualization using R (https://www.R-project.org/) and the packages Pheatmap (https://cran.r-project.org/web/packages/pheatmap/index.html), RColorBrewer (https://cran.r-project.org/web/packages/RColorBrewer/index.html), clusterProfiler(Yu et al., 2012) and org.Mm.eg.db (http://bioconductor.org/packages/release/data/annotation/html/org.Mm.eg.db.html).

#### Ultra-deep targeted sequencing

Epidermal whole mounts, approximately 4 mm x 4 mm per piece, were prepared from UV-exposed and adjacent unexposed areas of the skin as described above. This protocol was run for both induced and non-induced *p53*^*∗/wt*^ animals. DNA was extracted using QIAGEN DNA micro kit (QIAGEN). A panel of 74 genes was chosen (see list below) based on the criteria of genes highly mutated in cutaneous squamous cell carcinomas and/or basal cell carcinomas, as well as genes frequently mutated in normal skin samples. The genes sequenced were:

Aff3, Ajuba, Arid1a, Arid2, Arid5b, Atm, Atp2a2, Bcl11b, Braf, Cacna1d, Card11, Casp8, Ccnd1, Cdkn2a, Cobll1, Crebbp, Ct*cf.*, Ctnnb1, Dclk1, Dclre1a, Dnmt3a, Ddr2, Egfr, Eif2d, Ep300, Erbb2, Erbb3, Erbb4, Ezh2, Fat1, Fat2, Fat3, Fat4, Fbxw7, Fbxo21, Fgfr3, Flt3, Grin2a, Hras, Kdm6a, Kdr, Kit, Kmt2c, Kmt2d, Kras, Lrp1b, Mtor, Nf1, Nf2, Notch1, Notch2, Notch3, Notch4, Nras, Pik3ca, Ptch1, Pten, Rb1, Ros1, Smad4, Smarca4, Smo, Sox2, Stat5b, Tert, Tet2, Tgfbr1, Tgfbr2, Trp53, Tsc1, Vhl, Zfp750, Nrf2, Keap1.

A custom bait capture panel (Agilent) was designed using Agilent SureDesign, targeting the exonic sequences of these 74 genes.

Paired-end 75bp read sequencing was performed on an Illumina HiSeq 2000_v4 machine. After removing reads for off-target capture and PCR duplicates, the average on-target coverage across samples was 1476x. The paired-end reads were aligned to the reference mouse genome (GRCm38) using BWA-MEM (v0.7.15)(Li, 2013). Variants were called using the latest version of Shearwater (unpublished). Shearwater is a variant caller designed to detect low frequency somatic variants that can be challenging to find using more conventional variant callers designed for germline variants (Gerstung et al., 2014). To avoid the probability of false positive variant calls, Shearwater builds a model of the background error rate for each base in the genome. This error model is most accurate when using matched normal samples from the same or closely related individuals to those for which variants are being called (to avoid calling common germline variants) and processed in the same way in the lab (to avoid shared artifacts introduced during sample processing and sequencing).

After running Shearwater, we detect 5888 putative variants summing across all skin biopsies. To reduce the number of false-positives we then applied a series of filtering steps. First, we removed any variants detected in both irradiated and unexposed skin biopsies from the same mouse. These are unlikely to be true somatic variants because we do not expect somatic clones to extend across two distant biopsies. To correct for multiple-hypothesis testing we then applied the Benjamini-Hochberg correction for the biopsies in each mouse independently, retaining only mutations with an adjusted q-value of less than 0.1(Benjamini and Hochberg, 1995). Finally, we removed variants without at least one supporting read from the forward and reverse strand. This resulted in a total of 67 filtered variants. The variants called here do not include the *p53^∗^* mutation, as this is removed by the Shearwater algorithm as a germline variant. The variants were annotated using Ensembl Variant Effect Predictor (Version 84)(McLaren et al., 2016)

### Statistics

Source data and exact *P value*s for statistical tests are listed in the Table S7 for each Figure.

Statistical analysis was performed using the Graphpad Prism software. The D’Agostino-Pearson omnibus test was used to test for normality and the F-test to test for a significant difference in variance between groups. Student’s unpaired t test was performed for normally distributed data where there was no significant difference in variance between groups. A two tailed paired t test was used where applicable. For non-normally distributed data, a two tailed Mann-Whitney test was performed.

No statistical method was used to predetermine sample size. The experiments were not randomized. The investigators were not blinded to allocation during experiments or outcome assessment.

### Quantitative Analysis and Modeling

For details of quantitative analysis of wild-type (Figure S3A and S3B) and *p53* mutant progenitor cell lineage tracing data (Figures S6A and S6B), the dynamics of mutant cells in the suprabasal cell layers (Figures S6C and S6D) and a quantitative model of clonal competition during long-term ultraviolet light exposure (Figures 7F and S7E–S7G and Video S1) see STAR Methods, Quantitative Analysis.

### Data Availability

Raw transcriptomic data can be viewed on https://www.ebi.ac.uk/ena using the following accession numbers: p53^wt/wt^, ERS1755594, ERS1755602, ERS1755610, ERS1755618; p53^∗/wt^, ERS1755595, ERS1755603, ERS1755611, ERS1755619; p53^∗/∗^, ERS1755596, ERS1755604, ERS1755612, ERS1755620; p53^R245W/R245W^ (untag), ERS1755597, ERS1755605, ERS1755613, ERS1755621.

The accession number for the ultra-deep targeted DNA sequencing data reported in this paper is ENA: ERP023080.

### Code Availability

Source code is accessible via Github:

https://github.com/PHJonesGroup/Murai_etal_SI_code

## Supporting Citations

The following references appear in the Supplemental Information: Gillespie, 1976, Gillespie, 1977, Moran, 1957, Tibor and Krapivsky, 2010.

## Supplemental Information

Document S1. Figures S1–S7 and Tables S2, S3, S4, and S5

Table S1. Differentially Expressed Genes from RNA-Seq Analysis, Related to Figures 2, S4, and S5

Table S6. Mutations Detected by Deep-Exome Sequencing Analyzed by Shearwater, Related to Figures 7 and S7 See STAR Methods for details.

Table S7. Source Data and Statistical Tests for Figures, Related to Figures 3, 4, 5, 6, 7, S2, S4, S5, and S7

Document S2. Article plus Supplemental Information
