# Supplementary material for: Epidermal Tissue Adapts to Restrain Progenitors Carrying Clonal p53 Mutations
Source: Cell Stem Cell. 2018 Nov 1;23(5):687–699.e8. doi: 10.1016/j.stem.2018.08.017 (PMC6224607; doi:10.1016/j.stem.2018.08.017)
Supplement: Document S1. Figures S1–S7 and Tables S2, S3, S4, and S5 [file mmc1.pdf]

Cell Stem Cell, Volume *23*

Supplemental Information

Epidermal Tissue Adapts to Restrain

Progenitors Carrying Clonal *p53* Mutations

Kasumi Murai, Greta Skrupskelyte, Gabriel Piedrafita, Michael Hall, Vasiliki Kostiou, Swee Hoe Ong, Tibor Nagy, Alex Cagan, David Goulding, Allon M. Klein, Benjamin A. Hall, and Philip H. Jones

Supplementary Materials for
Epidermal tissue adapts to restrain progenitors carrying clonal p53 mutations

Kasumi Murai, Greta Skrupskelyte, Gabriel Piedrafita, Michael Hall, Vasiliki Kostiou, Swee Hoe Ong, Tibor Nagy, Alex Cagan, David Goulding, Allon M Klein, Benjamin A Hall, Philip H Jones

This PDF file includes

Supplementary Figures and Legends

Figure S1. Related to Figures 3, 4 and 6

Figure S2. Related to Figure 3

Figure S3. Related to Figure 3

Figure S4. Related to Figures 2 and 3

Figure S5. Related to Figures 2 and 3

Figure S6. Related to Figures 3 and 4

Figure S7. Related to Figures 6 and 7

Supplementary Movie. Related to Figure 7

Supplementary Tables and captions, S1 to S7

Methods S1, Quantitative analysis, linked to STAR methods

Supplemental Figures and Legends

Figure S1

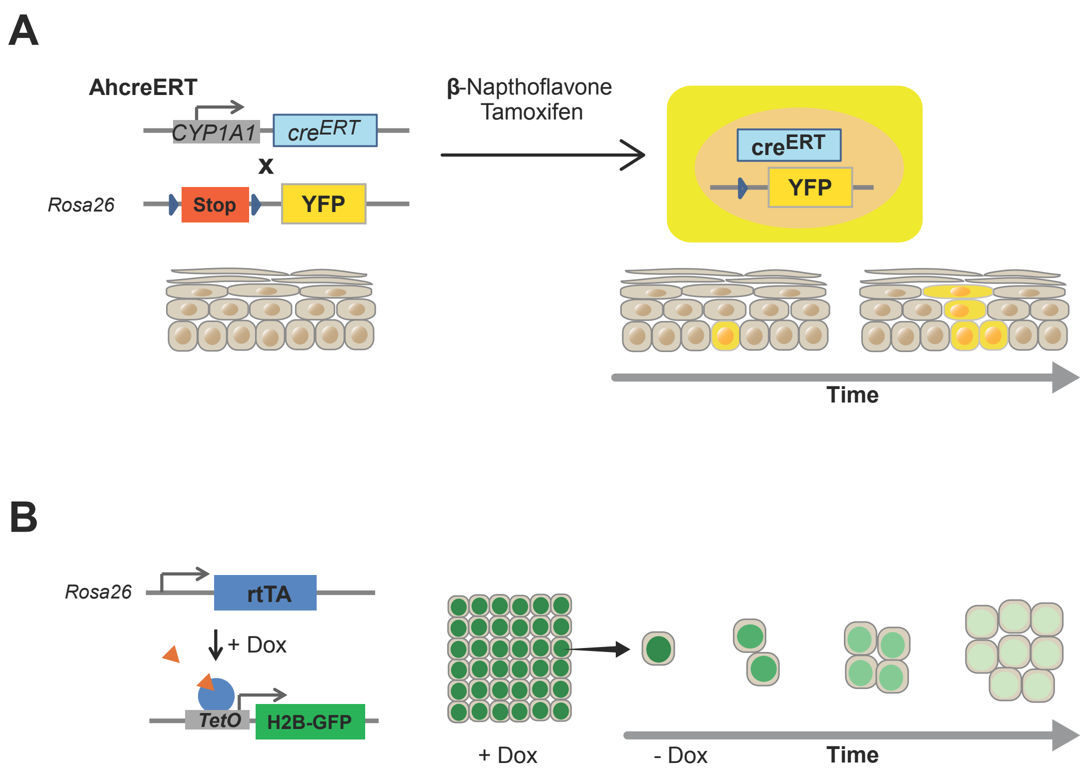

Figure S1. Related to Figures 3, 4 and 6; Transgenic assays used to track wild type cell behavior in this study

(A) Genetic lineage tracing in $Ahcre^{ERT}Rosa26^{flEYFP/wt}$ mice. *Cre*-mediated recombination, induced by β-napthoflavone (BNF) and tamoxifen (Tam), results in excision of stop cassette and the expression of the yellow fluorescent reporter (YFP). The reporter will be expressed in all the progeny of the recombined cell and form clusters of labelled cells termed clones.

(B) Quantification of the cell division rate. $R26^{M2rtTA}TETO\text{-}GFP$ animals carry a reverse tetracycline-controlled transactivator (rtTA-M2) and a HIST1H2BJ/EGFP fusion protein (HGFP) expressed from a tetracycline responsive promoter element. Doxycycline (dox) treatment in these animals results in nuclear labelling of all basal cells in the epidermis with HGFP. Upon withdrawal of dox, HGFP transcription ceases and the protein is diluted by cell division.

Figure S2

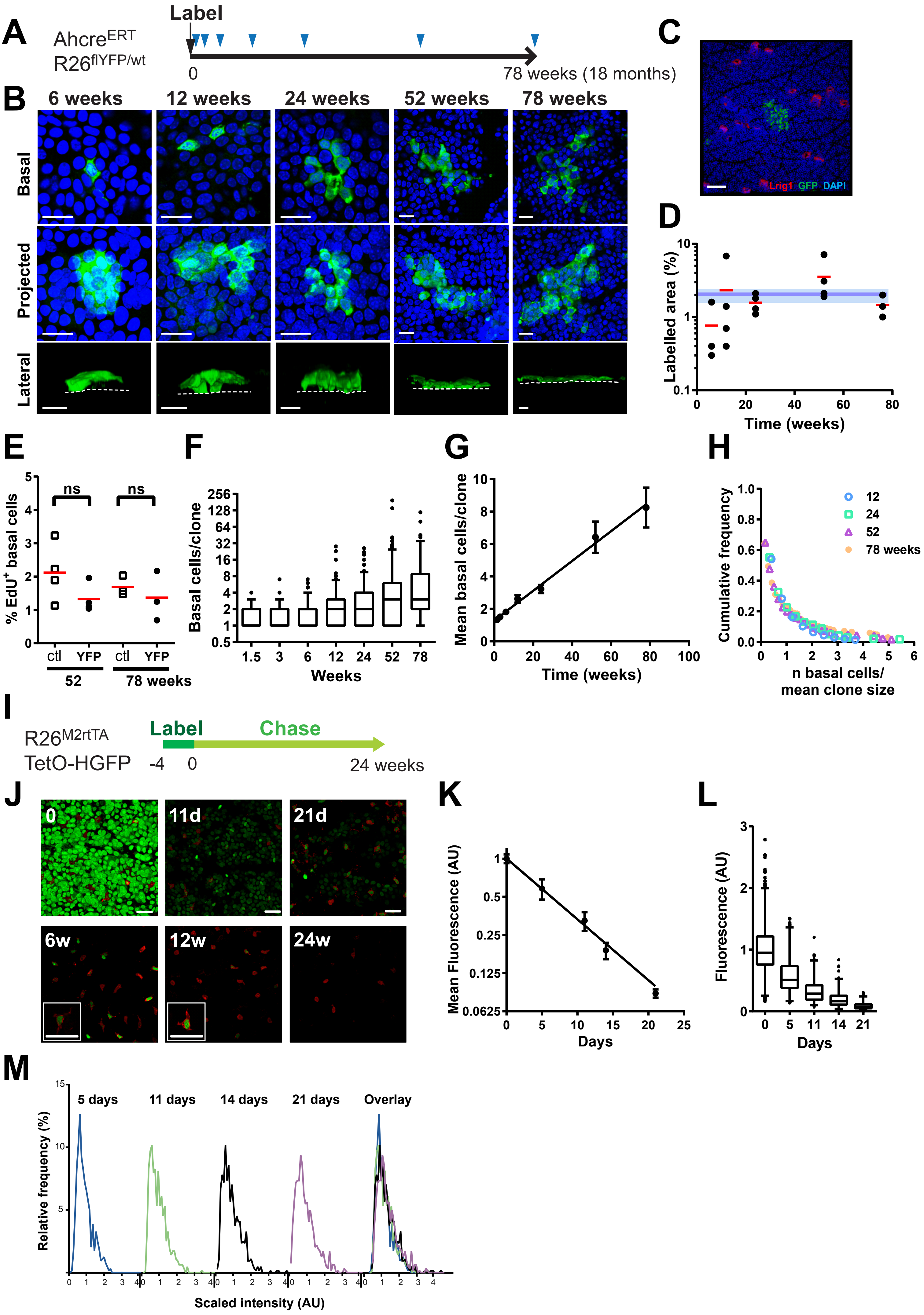

A
Label
AhcreERT R26flYFP/wt
0
78 weeks (18 months)
B
6 weeks
12 weeks
24 weeks
52 weeks
78 weeks
Basal
Projected
Lateral
C
Lrig1 GFP DAPI
D
Labelled area (%)
Time (weeks)
E
ns
ns
% EdU+ basal cells
ctl
YFP
52
78 weeks
F
Basal cells/clone
Weeks
G
Mean basal cells/clone
Time (weeks)
H
Cumulative frequency
12
24
52
78 weeks
n basal cells/ mean clone size
I
R26M2rtTA
TetO-HGFP
Label
Chase
24 weeks
J
0
11d
21d
6w
12w
24w
K
Mean Fluorescence (AU)
Days
L
Fluorescence (AU)
Days
M
5 days
11 days
14 days
21 days
Overlay
Relative frequency (%)
Scaled intensity (AU)

Figure S2. Related to Figure 3; Cell dynamics in wild-type mouse dorsal epidermis

(A) Lineage tracing protocol: Clonal frequency labelling was induced in the dorsal epidermis of *Ahcre*^{ERT} *R26*$^{flEYFP/wt}$ mice and samples collected at intervals from 11 days to 18 months (triangles).

(B) Rendered confocal z stacks showing typical clones in epidermal wholemounts at times indicated. Basal, top-down view of basal layer; projected, top-down view through all nucleated cell layers; lateral, side view of clone. White line, basement membrane; Green, YFP; blue, DAPI; Scale bars, 20 μm.

(C) Confocal image showing a representative clone 68 weeks post induction in IFE of mouse back skin. Hair follicles are marked by high *Lrig1* expression (red). Green, YFP; blue DAPI. Scale bar, 100 μm. 16 clones were imaged and all confined to the IFE.

(D) Average % projected labelled area at indicated time points. Values are from 5 fields per mouse. Red lines indicate mean value at each time point. Blue line and shading show average and s.e.m. across all time points from 6 weeks to 72 weeks. n = 18 animals.

(E) Percentage of EdU labelled basal cells in YFP clones and in non labelled cells (ctl) in same mouse at 1 year (52 weeks) and 78 weeks. n = 4 mice at 52 weeks and n = 3 mice at 78 weeks. ns, no significant difference by paired t test.

(F) Clone size distribution (basal cells per clone, for ≥ 2-cell clones). Box plots show median (line across box), 25th and 75th percentiles (box) and 5th and 95th percentiles (whiskers), dots are outliers (dots), at indicated time points. Epidermal wholemounts from at least 3 mice per time point were analyzed. n = 93 clones at 1.5 weeks, 106 clones at 3 weeks, 181 clones at 6 weeks, 183 clones at 12 weeks, 209 clones at 24 weeks, 266 clones at 52 weeks and 140 clones at 78 weeks.

(G) Mean basal cells per clone at each time point is shown; increase is linear, $r^2 = 0.99$. Error bars indicate s.e.m of clones.

(H) Cumulative clone size distribution showing convergence onto scaling behavior. Cumulative size distribution, Cn(t), plotted as a function of number of basal cells per clone, n, normalized by the average clone size at each time point.

(I) Protocol: Adult *Rosa26*^{M2rTA}*/TetO-H2BGFP* mice treated with doxycycline (DOX) to express HGFP (Green). Following Dox withdrawal, HGFP transcription ceases and the protein is diluted upon cell division. Slow-cycling cells retain higher levels of HGFP protein. Samples were taken at 0, 5, 11, 21 day, 6, 12 and 24 week time points. Cells in

hair follicle opening regions were excluded from the analysis.

(J) Rendered confocal z stacks of mouse back skin epidermis showing HGFP (green) and immunostaining for the pan leukocyte marker CD45 (red) at indicated times. Scale bar, 30 µm. Insets: typical appearance of label retaining cells (LRC) at 6 & 12 weeks. All LRCs were CD45 positive (total 310 LRCs, n = 4 mice at 6 weeks).

(K) Dilution of HGFP in epidermis quantified by confocal imaging up to 21days time point . Values are mean fluorescence at time points indicated. Error bars are s.e.m of individual intensities. 160 nuclei / animal are quantified. n = 4 mice per time point.

(L) Quantification of HGFP dilution by confocal imaging. Box plots show median (central line), 25th and 75th percentiles (box) and 1st and 99th percentiles (whiskers) and outliers (dots), n = 640 nuclei from 4 mice at each time point. AU, arbitrary units.

(M) HGFP intensity distributions scale with time. Graphs show distributions of HGFP intensity divided by the average intensity of all values at that time point. The scaled distributions overlie each other (overlay), suggesting the cells belong to a single population in terms of HGFP dilution.

Figure S3

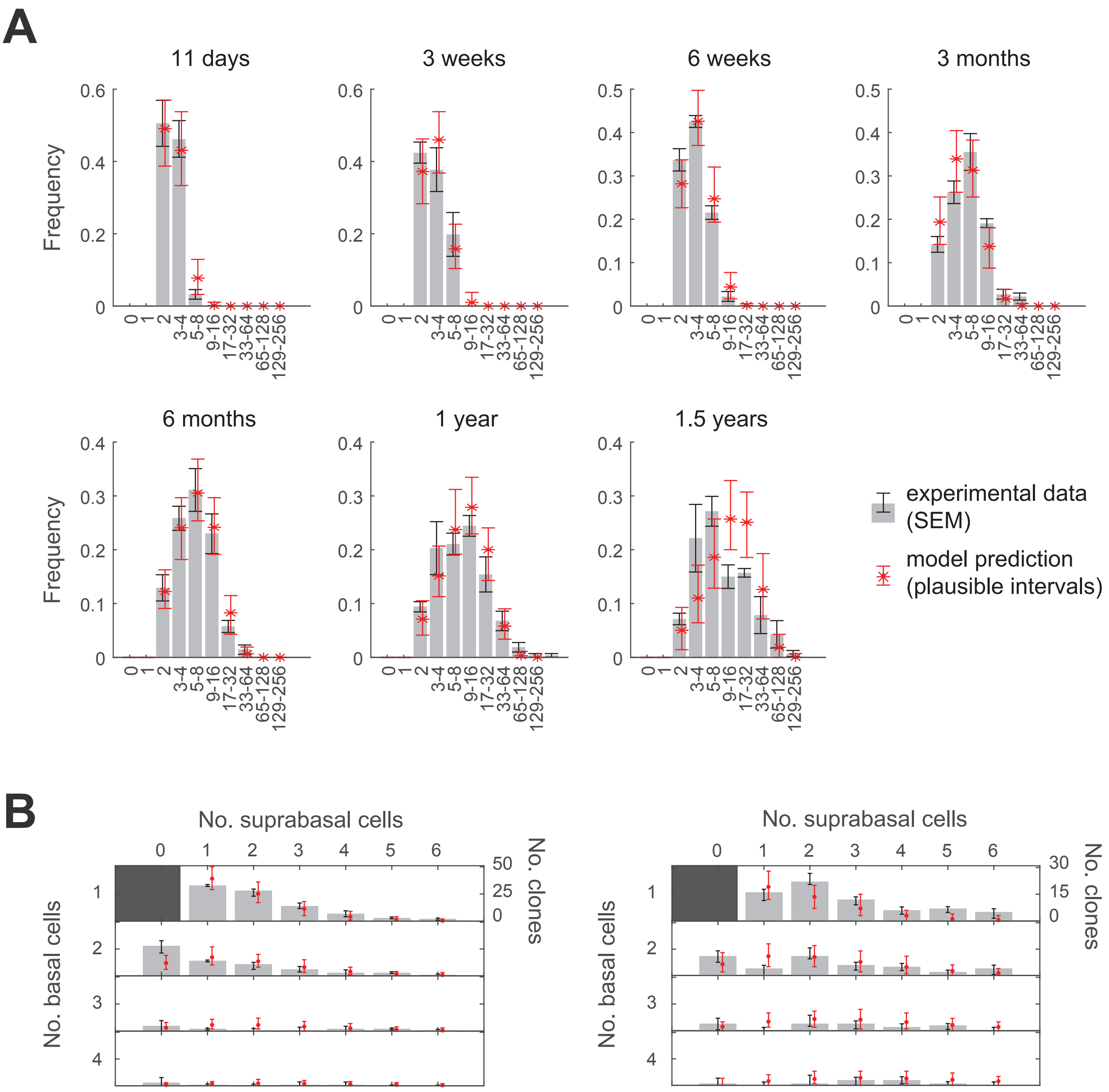
A
11 days
3 weeks
6 weeks
3 months
6 months
1 year
1.5 years
Frequency
0 1 2 3-4 5-8 9-16 17-32 33-64 65-128 129-256
experimental data (SEM)
model prediction (plausible intervals)
B
No. suprabasal cells
No. basal cells
No. clones
0 1 2 3 4 5 6
50 25 0
30 15 0

Figure S3. Related to Figure 3; *p53*$^{wt/wt}$ clone-size dynamics fit to single-progenitor model

(A) Total clone-size distributions obtained experimentally at different time points (grey bars show mean values; error bars refer to SEM; n = 3-5 mice per time point) are compared with the results of simulation of a single-progenitor model with balanced fates, with parameter values corresponding to those of the maximum likelihood estimate (red marks; see **Methods S1**) (the experimental suprabasal-to-basal cell ratio was further used to infer total sizes from basal estimates). Error bars in the model (red) represent *plausible intervals* defined as 95% likelihood intervals for each frequency value knowing the total number of clones observed at each time.

(B) Detailed clone sizes at early (6 weeks; left panel) and late (6 months; right panel) time points; frequencies displayed for *n* basal and *m* suprabasal clone sizes. Experimental data and model fit represented as in (A). In all cases floating clones (containing no basal cells) and single-cell clones were excluded from the analysis.

Figure S4

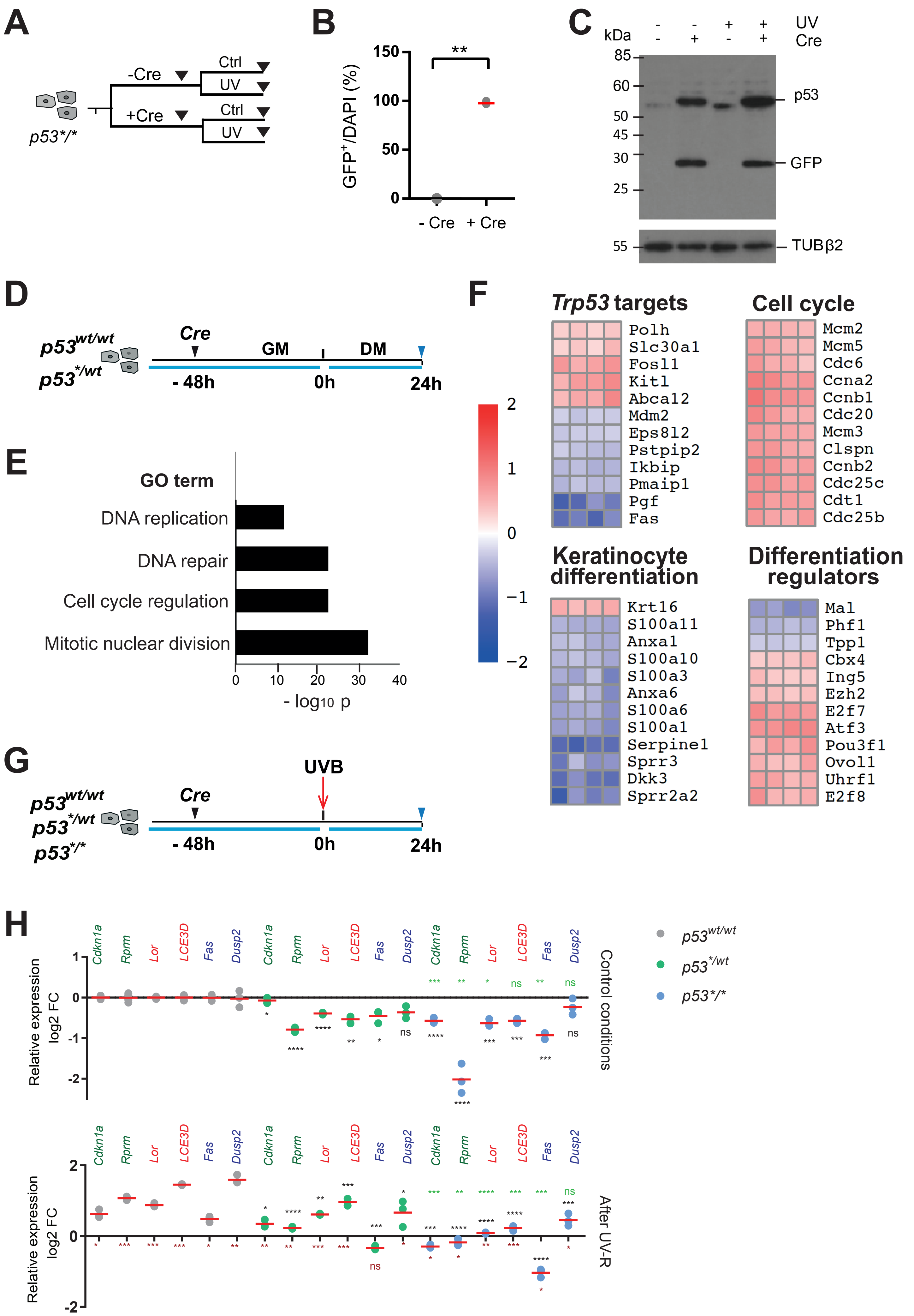
A
-Cre
+Cre
Ctrl
UV
Ctrl
UV
p53*/*
B
150
100
50
0
**
GFP+/DAPI (%)
- Cre
+ Cre
C
kDa
UV
Cre
85
60
50
45
30
25
55
p53
GFP
TUBβ2
D
p53wt/wt
p53*/wt
Cre
GM
DM
- 48h
0h
24h
E
GO term
DNA replication
DNA repair
Cell cycle regulation
Mitotic nuclear division
0
10
20
30
40
- log10 p
F
Trp53 targets
Polh
Slc30a1
Fosl1
Kitl
Abca12
Mdm2
Eps8l2
Pstpip2
Ikbip
Pmaip1
Pgf
Fas
Cell cycle
Mcm2
Mcm5
Cdc6
Ccna2
Ccnb1
Cdc20
Mcm3
Clspn
Ccnb2
Cdc25c
Cdt1
Cdc25b
Keratinocyte differentiation
Krt16
S100a11
Anxa1
S100a10
S100a3
Anxa6
S100a6
S100a1
Serpine1
Sprr3
Dkk3
Sprr2a2
Differentiation regulators
Mal
Phf1
Tpp1
Cbx4
Ing5
Ezh2
E2f7
Atf3
Pou3f1
Ovol1
Uhrf1
E2f8
2
1
0
−1
−2
G
UVB
p53wt/wt
p53*/wt
p53*/*
Cre
- 48h
0h
24h
H
Cdkn1a
Rprm
Lor
LCE3D
Fas
Dusp2
Relative expression
log2 FC
Control conditions
After UV-R
p53wt/wt
p53*/wt
p53*/*

Figure S4. Related to Figure 2 and 3; Validation of conditional *p53* mutant transgenic mouse model

(A) Protocol for *in vitro* recombination: homozygous $p53^{*/*}$ mutant keratinocytes were either treated or not treated with Cre and either exposed or unexposed to UV-B to stabilize p53.

(B) Recombination efficiency in $p53^{*/*}$ keratinocytes assessed by GFP reporter. A graph shows GFP positive cells over DAPI. **p = 0.0022 by Mann Whitney test. n = 3 biological replicates.

(C) Expression of p53 R245W mutant protein following *cre*-mediated recombination *in vitro*. Immunoblot shows the expression of p53 mutant protein and GFP reporter at expected size respectively. Tubulin β2 was used as loading control. Note mutant protein is more stable than wild type p53 protein so can be detected without UV-irradiation.

(D) Protocol: Primary keratinocytes from $p53^{wt/wt}$ or $p53^{*/wt}$ mice were cultured in growth media (GM) and infected with adenovirus encoding *cre* recombinase (black arrow). Cells were then switched to differentiation media (DM) and lysed after 24 hours (blue arrow).

(E) GO enrichment analysis associated with biological processes in differentially-expressed genes (selected categories with $-Log_{10}$ p, adjusted for multiple testing, are shown).

(F) Heatmaps showing selected differentially expressed genes in $p53^{*/wt}$ and wild-type cultures. Color scale indicates log_2 fold ratio, $p53^{*/wt}$ /$p53^{wt/wt}$, all genes shown have adjusted p value < 0.05. Full list of differentially expressed genes is in **Table S1**.

(G) Primary keratinocytes were isolated from $p53^{wt/wt}$, $p53^{*/wt}$ and $p53^{*/*}$mice. 48 hours after *in vitro cre*-mediated recombination, cells were exposed to 10 mJ/cm^2 UVB and transcriptional analysis was performed 24 hours later.

(H) Quantitative RT-PCR analysis for UV-inducible genes, *Cdk1na*, *Rprm* (cell cycle), *Lor*, *Lce3d* (terminal differentiation) and *Fas*, *Dusp2* (Apoptosis) with or without UVB irradiation (UV-R). Data are normalized housekeeping gene *Tubb2b* and presented as log2 fold change relative to $p53^{wt/wt}$ under control conditions. n = 3 independent experiments. Red horizontal line indicates the mean value. *p < 0.05; **p < 0.01; ***p < 0.001; ns, no significance; significance between genotypes was determined by two-tailed unpaired t-test, and between conditions (±UVB) by paired t-test. Black asterisks, comparison between $p53^{wt/wt}$ and mutant; Green asterisks, comparison between $p53^{*/wt}$

and *p53*$^{*/*}$mutants; Red asterisks, comparison between UV exposed and control conditions.

Figure S5

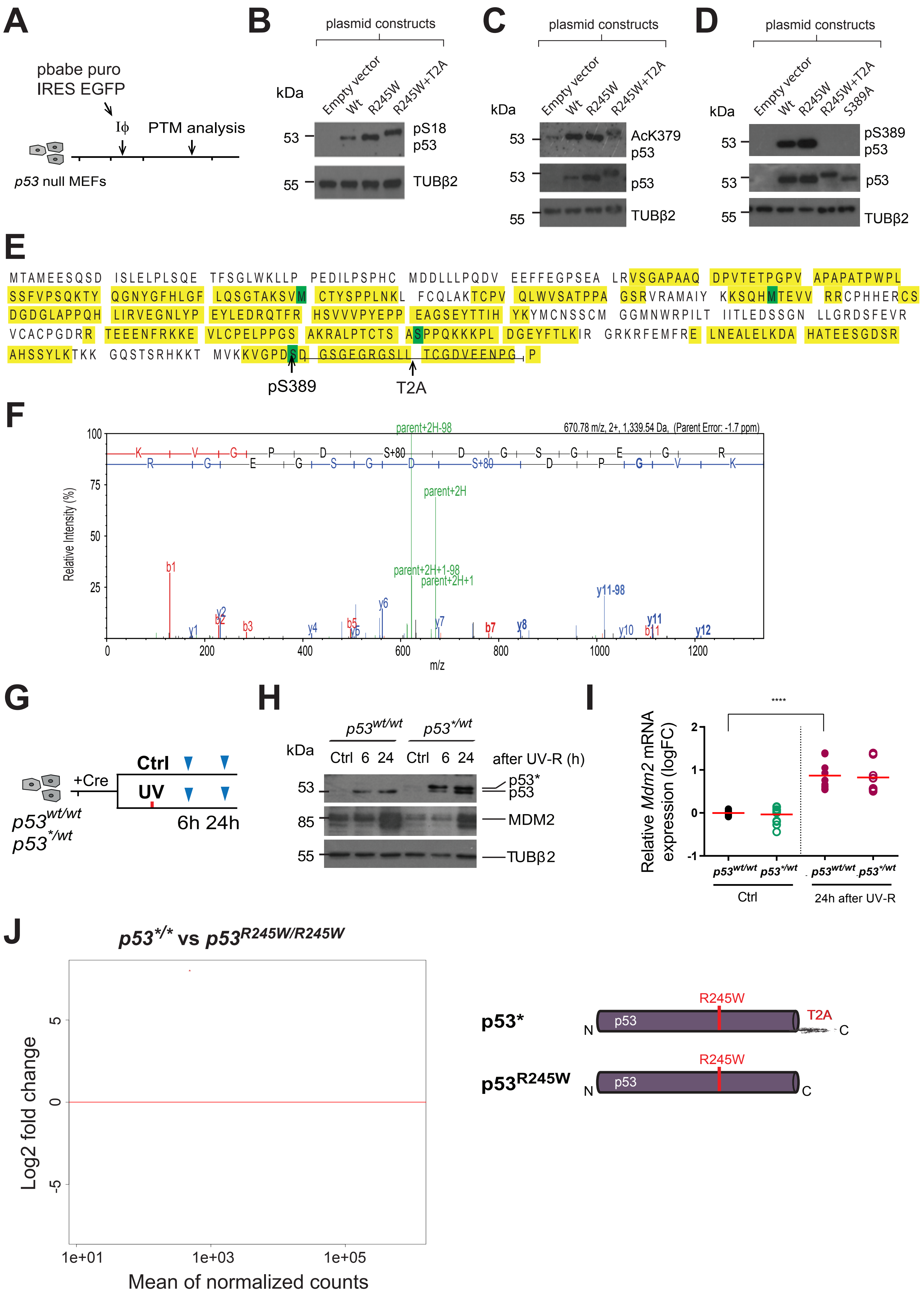
A
pbabe puro
IRES EGFP
Iϕ
PTM analysis
p53 null MEFs
B
plasmid constructs
Empty vector
Wt
R245W
R245W+T2A
kDa
53
55
pS18
p53
TUBβ2
C
plasmid constructs
Empty vector
Wt
R245W
R245W+T2A
kDa
53
53
55
AcK379
p53
p53
TUBβ2
D
plasmid constructs
Empty vector
Wt
R245W
R245W+T2A
S389A
kDa
53
53
55
pS389
p53
p53
TUBβ2
E
MTAMEESQSD ISLELPLSQE TFSGLWKLLP PEDILPSPHC MDDLLLPQDV EEFFEGPSEA LRVSGAPAAQ DPVTETPGPV APAPATPWPL
SSFVPSQKTY QGNYGFHLGF LQSGTAKSVM CTYSPPLNKL FCQLAKTCPV QLWVSATPPA GSRVRAMAIY KKSQHMTEVV RRCPHHERCS
DGDGLAPPQH LIRVEGNLYP EYLEDRQTFR HSVVVPYEPP EAGSEYTTIH YKYMCNSSCM GGMNWRPILT IITLEDSSGN LLGRDSFEVR
VCACPGDRR TEEENFRKKE VLCPELPPGS AKRALPTCTS ASPPQKKKPL DGEYFTLKIR GRKRFEMFRE LNEALELKDA HATEESGDSR
AHSSYLKTKK GQSTSRHKKT MVKKVGPDSD GSGEGRGSLL TCGDVEENPG P
pS389
T2A
F
670.78 m/z, 2+, 1,339.54 Da, (Parent Error: -1.7 ppm)
parent+2H-98
parent+2H
parent+2H+1-98
parent+2H+1
K V G P D S+80 D G S G E G R
R G E G S G D S+80 D P G V K
Relative Intensity (%)
100
75
50
25
b1
y1
y2
b2
b3
y4
b5
y5
y6
y7
b7
y8
y11-98
y10
b11
y11
y12
0
200
400
600
800
1000
1200
m/z
G
+Cre
Ctrl
UV
p53wt/wt
p53*/wt
6h 24h
H
kDa
p53wt/wt
p53*/wt
Ctrl 6 24
Ctrl 6 24
after UV-R (h)
53
85
55
p53*
p53
MDM2
TUBβ2
I
****
Relative Mdm2 mRNA
expression (logFC)
2
1
0
-1
p53wt/wt
p53*/wt
p53wt/wt
p53*/wt
Ctrl
24h after UV-R
J
p53*/* vs p53R245W/R245W
Log2 fold change
5
0
-5
1e+01
1e+03
1e+05
Mean of normalized counts
p53*
N
p53
R245W
T2A
C
p53R245W
N
p53
R245W
C

Figure S5. Related to Figure 2 and 3; Characterization of p53* protein

(A-F) p53 post-translational modifications (PTM):

(A) Primary *p53* null mouse embryonic fibroblasts were infected with retroviruses carrying either wild type *p53* or one of the mutants *p53*^{R245W}, *p53*$^{R245W+T2A}$ or *p53*^{S389A}. PTM were analysed using total cell lysate .

(B-D) Immunoblots showing p53 phosphorylation at Ser18 (B) and Ser389 (C), and acetylation at Lys379 (D). Tubulin β2 was used as loading control. Anti–Ser389 antibody failed to detect phosphor-p53$^{R245W+T2A}$.

(E and F) Confirmation of phosphorylation of p53$^{R245W+T2A}$ at Ser389 by mass spectrometry analysis. (E) The analysis covered 62% of *Mus Musculus p53* variant 1 sequence (GenBank: AAB03324.1), which is highlighted in yellow. Green, phosphorylated amino acids. (F) The plot shows the relative current intensity produced by phospho-ions of varying mass-to-charge ratio (*m/z*).

(G) Protocol for *in vitro* protein stability study: *p53*$^{wt/wt}$ and *p53*$^{*/wt}$ mutant keratinocytes were *cre* recombined and exposed to UV-R. Cell lysate collected at 6 and 24 hours post irradiation.

(H) Immunoblot of p53 and its negative regulator, MDM2, before and after UV-irradiation in *p53*$^{wt/wt}$ and *p53*$^{*/wt}$ keratinocytes. A representative blot from three independent experiments is shown, Tubulin β2 is loading control.

(I) Quantitative RT-PCR showing the transcription of *Mdm2*. Data was normalised to *Tubb2b* expression and shown as $\log_2$ transformed value. n = 4 independent experiments, significance determined by Mann Whitney test, ****$P < 0.0001$.

(J) Confirmation of functional similarity between p53* and untagged p53^{R245W}. Primary keratinocyte from *p53*$^{*/*}$ and untagged *p53*$^{R24W/R245W}$ mice were subjected to *in vitro* cre-mediated recombination and kept in growth media for 48 hours. Cells were then switched into culture media permissive of differentiation and analysed by RNAseq 24 hours later. MA plot shows the differentially expressed genes between *p53*$^{*/*}$ and untagged *p53*$^{R24W/R245W}$ primary keratinocyte cultures. The black dots represent the genes with no significant difference. We detected only 22 significantly (DEseq2 padj < 0.05) differentially expressed genes shown in red and listed in **Table S1**, indicating retained T2A peptide has negligible effect on the p53^{R245W} activity. Right: scheme showing tagged (p53*) and untagged (p53$^{R245W)}$ form of mutant proteins.

Figure S6

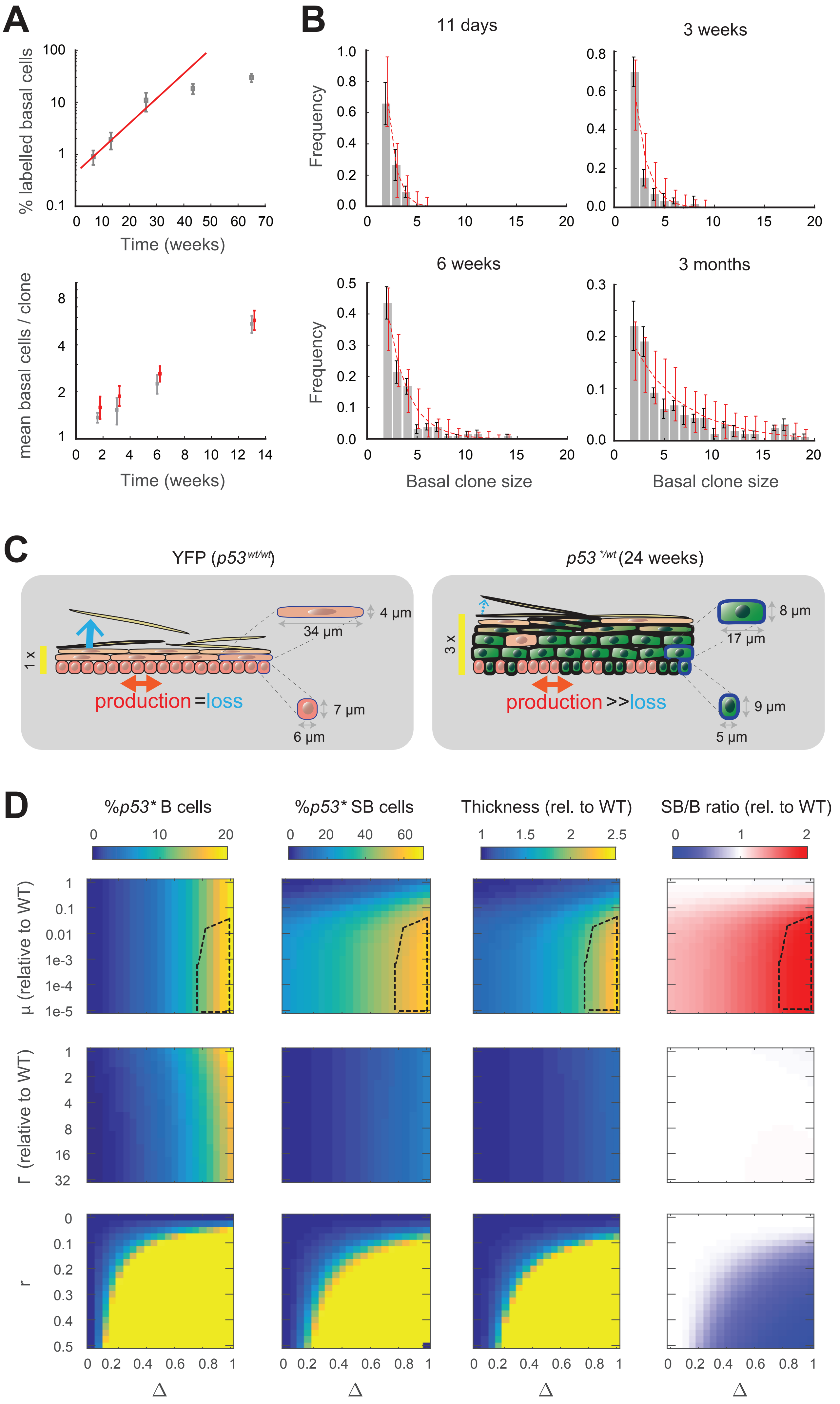

A
% labelled basal cells
100
10
1
0.1
Time (weeks)
mean basal cells / clone
Time (weeks)
B
11 days
3 weeks
6 weeks
3 months
Frequency
Basal clone size
C
YFP (p53 wt/wt)
p53 */wt (24 weeks)
1 x
3 x
4 µm
34 µm
7 µm
6 µm
8 µm
17 µm
9 µm
5 µm
production = loss
production >> loss
D
%p53* B cells
%p53* SB cells
Thickness (rel. to WT)
SB/B ratio (rel. to WT)
µ (relative to WT)
1
0.1
0.01
1e-3
1e-4
1e-5
Γ (relative to WT)
1
2
4
8
16
32
r
Δ

Figure S6. Related to Figure 3 and 4; *p53*/wt* clonal dynamics fit with a single-progenitor model with fate imbalance

(A and B) Comparison of experimental and simulated results for the *p53*/wt* mutant population dynamics. Average percentage of labelled basal cells (A, top panel) and average basal clone sizes (A, bottom panel) are shown as well as the basal-layer clone size distributions over time (B). Experimental data are shown in grey (error bars indicate s.e.m.; n = 3-4 mice per time point). For the simulation, a single-progenitor model with unbalance fates was considered (see **Methods S1**). Error bars in the model (red) represent *plausible intervals* defined as 95% likelihood intervals for each frequency value knowing the total number of clones observed at each time. The population growth trend obtained through simulation is overlaid on **A**, top panel.

(C) Schematic: Changes in the epidermal structure between *p53wt/wt* (WT) and *p53*/wt* mice (6 months post-induction). Pink, *p53wt/wt* cells. Green, *p53*/wt* expressing cells. Typical values of cell sizes measured in each case in the basal and first suprabasal layers are displayed for comparison. These were used in cell-population dynamics simulations to infer epidermal thickness under different assumptions (see D). Both the pattern of *p53*/wt* cell colonization and the tissue thickening are consistent with a substantial reduction in shedding rate (blue arrows).

(D) Deterministic analysis of the *p53*/wt* mutant cell population dynamics and tissue properties under different parameter conditions (see **Methods S1**). Displayed are simulation results for the proportion of *p53** cells in the basal and suprabasal layers, the suprabasal-to-basal cell ratio, and tissue thickness at 24 weeks in *p53*/wt* induced epidermis. Measured cell sizes given in C were used for the latter. The degree of fate imbalance (Δ) was allowed to change along with shedding rate (μ), stratification rate (Γ), or division choice-related parameter (*r*). Only a substantial decrease in μ along with a Δ >> 0 could explain the observed accumulation of *p53*/wt* cells in the suprabasal layers and the increased thickness (dashed regions correspond with those parameter values yielding tissue properties that are quantitatively consistent with the observed data).

Figure S7

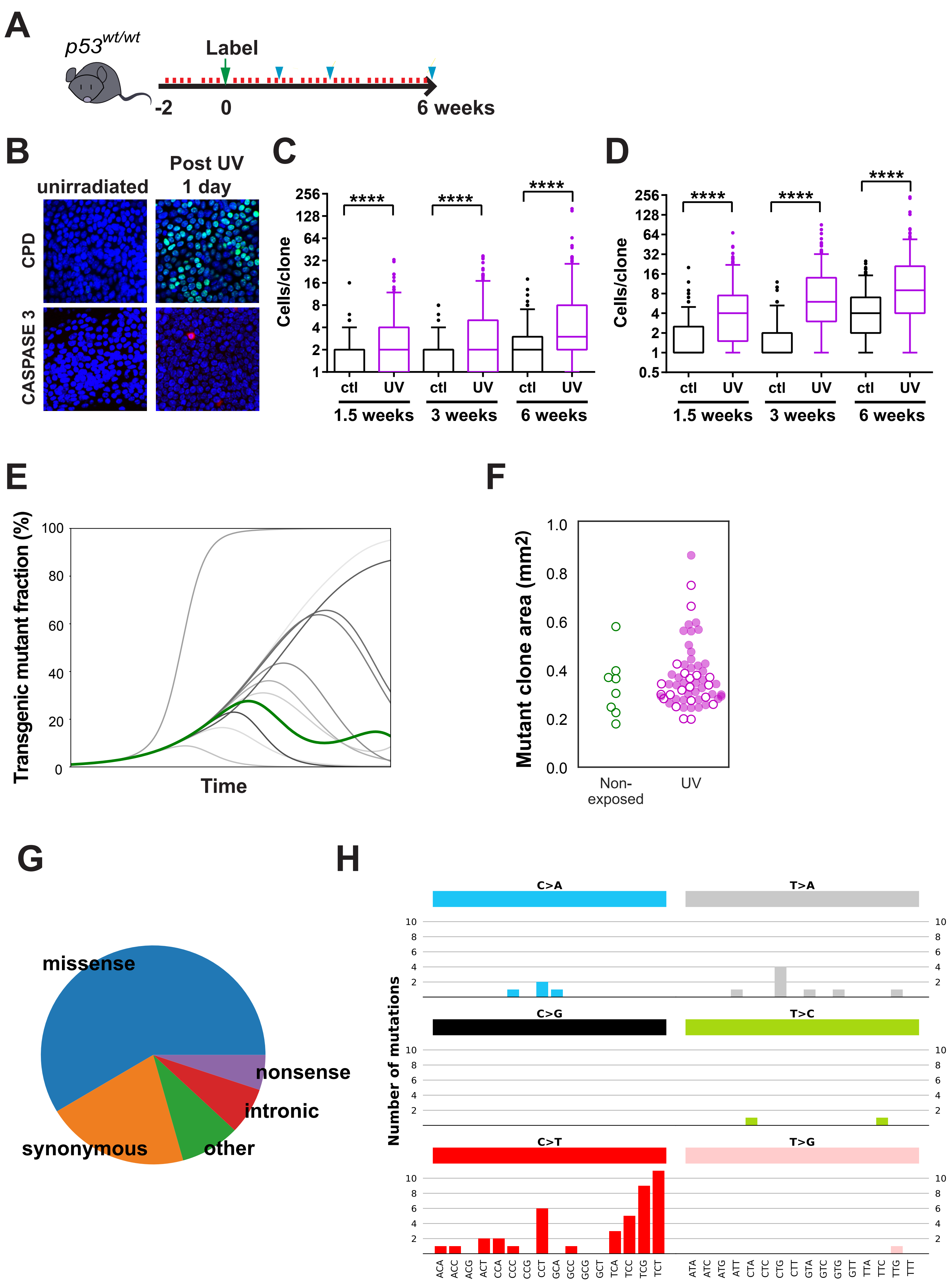

A
p53wt/wt
Label
-2
0
6 weeks
B
unirradiated
Post UV 1 day
CPD
CASPASE 3
C
Cells/clone
256
128
64
32
16
8
4
2
1
****
ctl
UV
1.5 weeks
3 weeks
6 weeks
D
Cells/clone
0.5
E
Transgenic mutant fraction (%)
100
80
60
40
20
0
Time
F
Mutant clone area (mm2)
1.0
0.8
0.6
0.4
0.2
0.0
Non-exposed
UV
G
missense
nonsense
intronic
synonymous
other
H
Number of mutations
C>A
T>A
C>G
T>C
C>T
T>G
10
8
6
4
2
ACA ACC ACG ACT CCA CCC CCG CCT GCA GCC GCG GCT TCA TCC TCG TCT
ATA ATC ATG ATT CTA CTC CTG CTT GTA GTC GTG GTT TTA TTC TTG TTT

Figure S7. Related to Figures 6 and 7; Titration of UVB dose and effect of UV exposure

(A) Protocol: mice were exposed to a sub-erythema dose of UVB daily, 4 days per week for 2 weeks (red lines) with shaving on the 5th day, labelling was induced and subsequently samples were collected (blue arrows).

(B) Confocal images showing basal layer of back skin epidermis. Animals were culled 24 hrs after 750 J/m^2 of UV-irradiation and wholemounts samples were prepared from UV-irradiated or unirradiated (ctl) back skin. Green, CPD; Red, Caspase-3; Blue, DAPI.

(C and D) Box plots showing basal (C) and total (D) clone size distribution in UV-irradiated epidermis (purple) and adjacent unexposed IFE (black) of induced *AhcreERT-RYFP* mice. Box plots show median (line across box), 25th and 75th percentiles (box) and 5th and 95th percentiles (whiskers), dots are outliers (dots), at indicated time points. UV, n = 220 clones at 1.5 weeks, n = 283 clones at 3 weeks, n = 229 clones at 6 weeks; control, n = 132 clones at 1.5 weeks, n = 131 clones at 3 weeks, n = 198 clones at 6 weeks. n = 4 mice per each time point. ****$p < 0.0001$ by by Mann Whitney test.

(E-G) Effect of long term UV exposure (see **Figure 7A** for the protocol) (E) Simulations of clone competition under ongoing mutagenesis (see **Supplementary Note 1.5** for details and **Supplementary Movie)**. A transgenic mutant is induced at 1% frequency in a background of wild-type cells. Subsequently, new mutations occur at random and are assigned a fitness value as described in Supplementary Note 1.5. The time course of the proportion of transgenic mutant cells in 10 independent simulations is shown. Green line indicates the outcome of the simulation captured in **Figure 7F**. (F) Estimated sizes of mutant clones detected in back skin biopsies of *p53*/wt* mice by the Shearwater algorithm (**Methods**). Clone sizes estimated by doubling the observed variant allele fraction (assuming the mutations were heterozygous). The induced $p53^{R245W}$ mutation was not detectable by this method and is excluded. Solid circles, mutations from non-induced samples; open circles, mutations from induced samples. Green, non-exposed skin; Purple, UV irradiated. (G) Functional annotation of all mutations from the 36 week UV irradiated skin biopsies, according to Ensembl Variant Effect Predictor (Version 84). The "other" category includes "splice_region_variant", "splice_acceptor_variant" and "coding_sequence_variant".

(H) Trinucleotide plot of mutations in 36 week UV biopsies. The nucleotide change and immediate nucleotide context of all SNVs from the 36 week UV irradiated skin biopsies. It can be seen that the large majority of mutations are C>T, a feature of the UV mutation signature.

Supplementary Movie. Related to Figure 7; Simulation of clone competition under ongoing mutagenesis.

A visualization of the *non-spatial* simulation shown in **Figure 7F.** A transgenic mutant (green) is induced at 1% frequency in a background of wild-type cells (yellow). Subsequently, new mutations (shades of green if in transgenic cells, other colors if in wild-type cells) are induced at random and assigned a fitness value as described in **Methods S1**.

Supplementary Tables and captions

Table S1. Related to Figure 2, S4 and S5; Excel spreadsheet showing differentially expressed genes from RNAseq analysis

Table S2. Related to Figure 3 and 4; Proportion of Caspase 3 positive cells in back skin epidermis

	Animal	Total nuclei (fields)	Caspase 3^+ cells	Proportion (%)
YFP (exp.1)	1	3007 (6)	15	0.499%
	2	2985 (6)	0	0.000%
p53$^{*/wt}$	1	2586 (6)	3	0.116%
	2	3161 (6)	2	0.063%
YFP (exp.2)	1	112050 (27)	2	0.002%
	2	95450 (23)	3	0.003%

Table S3. Related to Figure 4; Proportion of EdU positive basal cells in *p53*$^{*/wt}$ areas at late time points

	Time point		
	6 months	10 months	15 months
animal 1	1.9	1.2	2.4
animal 2	1.9		0.7
animal 3	0.5		

% EdU^+ basal cells per total GFP^+ basal cells

Table S4. Related to Figure 4; Tumor-free survival

Genotype	Time after induction (months)			
	3	6	12	15
Induced *YFP*	0/17	0/12	0/7	0/3
Induced *p53*$^{*/wt}$	0/23	0/20	0/15	0/4
Uninduced *p53*$^{*/wt}$	n.d.	n.d.	0/3	n.d.

Values are numbers of epidermal tumors/total number of mice at each time point

Table S5. Related to Figure 6 and S7; Proportion of Caspase 3 positive cells in UV irradiated *p53*$^{wt/wt}$ skin.

	Basal	Suprabasal
control	0.0%	0.0%
1 day	0.7%	0.9%
1 week	0.4%	0.4%
2 weeks	1.1%	0.0%
4 weeks	1.6%	0.0%

Table S6. Related to Figure 7 and S7; Excel spreadsheet showing mutations detected by deep exome sequencing analyzed by Shearwater, see **methods** for details.

Table S7. Related to Figure 3-7, S2, S4, S5 and S7
Excel spreadsheet showing source data and statistical tests for figures.

Methods S1: Quantitative analysis of lineage tracing and clonal competition, related to STAR materials

Here we first discuss experiments used to track wild type epidermal progenitor cells, described in the main text in more detail (**Section 1.1**), along with quantitative analysis of the results (**Section 1.2**). **Section 1.3** covers the *p53* mutant progenitor cell dynamics, **Section 1.4** the dynamics of mutant cells in the suprabasal cell layers and **Section 1.5** considers whether clonal competition is a possible explanation for the loss of *p53* mutant clones during long-term ultraviolet light exposure.

1.1 Experiments to track wild type epidermal progenitor cell behavior

In this section, we first consider the genetic lineage tracing of dorsal IFE progenitor cells in *Ahcre*^{ERT} *Rosa26*$^{flYFP/wt}$ mice introduced in the main text (**Figures S1A and S2A-S2H)**. In this experiment, a heritable genetic label (YFP) is induced at low frequency and is expressed in individual proliferating cells and their progeny. A key issue is whether the YFP-labelled cells are representative of all proliferating cells in the interfollicular epidermis (IFE). We noted that the proportion of labelled basal cells in IFE remained approximately constant over the duration of the experiment, indicating the YFP$^+$ population was self-sustaining and exhibited the cellular homeostasis characteristic of the entire IFE (**Figure S2D)**. Furthermore, there was no significant difference in the proportion of S-phase cells within YFP$^+$ clones and the surrounding non-labelled epidermis at 12 and 18 months after induction (**Figure S2E**). Taken together these results argue that labelled cells constitute a representative sample of proliferating epidermis. The characteristics of the evolving YFP$^+$ clone size distributions are discussed below in **section 1.2**.

A second experiment examined the rate of proliferation of cells in the epidermis by using the dilution of a transiently expressed transgenic histone–green fluorescent protein fusion (HGFP) to infer the mean rate of epithelial cell division (**Figures S1B and S2I-S2M)**(Doupe et al., 2012). Transgenic animals were treated with doxycycline (Dox) to induce HGFP transcription from a synthetic Dox-regulated promoter, resulting in high-level expression of HGFP throughout the epidermis (**Figures S1B, S2I and S2J**). Following withdrawal of Dox, HGFP levels were measured by confocal imaging of

epidermal whole mounts. Histone mRNA is short-lived, so HGFP transcript is unlikely to persist following Dox removal (Harris et al., 1991; Mascre et al., 2012). Assuming that the rate of HGFP protein degradation is small compared with the cell cycle time, the rate of HGFP decrease will reflect the rate at which it is diluted by cell division(Doupe et al., 2012). Slow cycling or quiescent cells will retain HGFP protein for long periods. We detected such HGFP retaining cells in IFE but all stained positive for the pan leukocyte marker CD45. No keratinocyte label-retaining cells were observed (**Figure S2J**). The mean level of HGFP in IFE keratinocytes fell with a half-life of 6 days and the form of the (unimodal) distribution scaled with time (**Figures S2K-S2M).** These results indicate that all cycling keratinocytes in dorsal skin IFE divide at a similar rate.

1.2. Wild type epidermal progenitor cell dynamics

Analysis of the evolving clone size distributions was used to test whether cell behavior in murine dorsal epidermis was consistent with the single-progenitor population model proposed in earlier work on squamous epithelial tissues (**Figure 1B**)(Clayton et al., 2007; Doupe et al., 2012). According to this model, the basal layer is populated by a single type of progenitor cell (A-cells) and differentiating cells (B-cells) which are post-mitotic. A-cells divide at a rate λ to give two daughter cells whose fates are determined stochastically: with a certain probability, the divisions are symmetric, yielding either two proliferating cells (A+A) or two differentiated cells (B+B), with the other divisions being asymmetric (A+B). Differentiating B cells leave the basal layer with a stratification rate Γ and become suprabasal cells (C-cells) that are ultimately lost by shedding at a rate μ.

$$
\begin{aligned}
&A \xrightarrow{\lambda} \begin{cases} A+A & \text{Prob. } r \\ A+B & \text{Prob. } 1-2r \\ B+B & \text{Prob. } r \end{cases} \\
&B \xrightarrow{\Gamma} C \qquad (1) \\
&C \xrightarrow{\mu} \emptyset
\end{aligned}
$$

In the simplest scenario, the probabilities that symmetric divisions lead to two proliferating cells or two differentiated cells are balanced, i.e. both are defined by a fixed parameter *r*, ensuring tissue homeostasis (**Figure 1B**). Under these conditions, one can assume that the proportion of proliferating cells in the basal layer, denoted as ρ, remains constant, and overall, the net rate at which post-mitotic cells are generated in the basal layer is compensated for by cell stratification and shedding (**Figure 1A**). It follows that

some relationships between the parameters can be established at homeostasis. In particular, $\Gamma = \lambda\rho/(1-\rho)$, and $\mu = \lambda\rho/m$, where the parameter m corresponds to the global ratio of suprabasal-to-basal cell populations.

Evidence from the YFP genetic lineage tracing experiments (**Figure S2**, **Section 1.1**) argues that the dynamics of the back-skin epidermis in *p53* wild-type ($p53^{wt/wt}$) mice conforms to the single-progenitor model with balanced fates(Klein et al., 2007). Firstly, despite the high variability of YFP labelling efficiency between individual mice, the percentage of area covered by labelled clones remains approximately constant over time, at around 2% (**Figure S2D**). Secondly, the average size of the persisting clones (i.e. those containing at least 1 basal cell) continuously expands with time, and the clone size distributions adopt a scaling behavior at late time points (**Figures S2F and S2H**).Thirdly, the average basal-layer clone size growth shows a linear increase over time (**Figure S2G**). The slope, sometimes denoted as τ^{-1} , can be identified as $r\lambda/\rho$ (Clayton et al., 2007). When analyzing back-skin clone sizes we find that $r\lambda/\rho$ = 0.08 ± 0.01 week^{-1} (95% confidence interval). We also find that the ratio of suprabasal cells to basal cells among labelled clones converges into a stationary value. Measurements of suprabasal-to-basal cells within relatively large persisting clones (containing at least 4 basal and 4 suprabasal cells) at late time points (≥ 6 months) allowed us to estimate m as 1.07 ± 0.17 (95% confidence interval). Finally, the pattern of HGFP dilution over a period of 5-21 days keeps very homogeneous, indicating a single type of progenitor cell (**Figures S2K-S2M**). The fluorescence intensity I decays exponentially with time, following $I_t = I_{t=0} * 2^{-\lambda t}$, the fitting giving an average division rate λ = 1.16 ± 0.06 week^{-1} (95% confidence interval)(Frede et al., 2016).

To demonstrate the validity of the model and fit clone size distributions with a single parameter solution we performed inference analysis on the unknown parameters based on the experimental basal-layer clone size distributions at different times. Using prior average estimates of λ, m and the parameter constraints given by the condition of homeostasis (see relationships above), the clonal dynamics can be estimated from the identification of the independent parameters r and ρ. For this, we implemented a maximum likelihood estimation (MLE) approach, iterating on different values of the

parameters r and ρ (grid search). A log-likelihood value $l(\theta; x)$ was obtained for every parameter set θ in the following way:

$$l(\theta; x) = \sum_t \sum_n (x_n(t) * \log p_n(t, \theta)) \qquad (2)$$

where $x_n(t)$ is the observed frequency of clones with a basal size n at time t, and $p_n(t, \theta)$ is the probability of observing clones of that size at time t given the parameter values θ. Due to issues in computing the available analytical solution(Tibor and Krapivsky, 2010)in our analysis $p_n(t, \theta)$ was estimated for each parameter set from multiple simulations (100,000) of the Master equation, following Gillespie's algorithm (Gillespie, 1977; Gillespie, 1976). Given the particular scaling behavior of the clone size distributions and their large asymmetry, clone sizes were binned in ranges increasing in powers of two, as in previous work, i.e. n above stands for clones with a number of basal cells in the range 2^{n-1} to $(2^n - 1)$ (Clayton et al., 2007).

Only persisting clones with at least 2 basal cells were considered for the MLE analysis, to exclude any possible distortion due to post-mitotic cells labelled at induction. In addition, a small proportion (~1%) of late-time clones were reported with sizes that greatly exceeded the vast majority of their coexisting clones (i.e. sizes >> 2.3*SD above the mean clone size). These outlier clones were pooled in a common category (clones with >32 cells) to circumvent the computational issues of estimating the probability of extremely rare events with sufficient precision. However, excluding the outstandingly large clones from the analysis did not significantly alter the parameter estimates provided below.

The previous analysis gave the following maximum likelihood estimate on the parameters (95% confidence interval):

r = 0.06 (0.05; 0.09) ρ = 0.77 (0.69; 0.97)

This corresponds with the following values for the other (dependent) parameters: Γ = 3.9 (2.6; 38.0) week^{-1}, μ = 0.8 (0.6; 1.2) week^{-1}. Altogether, the MLE values $\hat{\theta}_{MLE}$ match well with the linear slope of the average clone size scaling, and the predicted clone size distributions fit well with the experimental total clone sizes over time, corroborating that the single progenitor model suits the cell dynamics in back skin epidermis (**Figures S3A and S3B**).

1.3. $p53^{*/wt}$ clonal expansion

In balanced, homeostatic conditions, the total labelled area remains constant, as seen in the data of the wild type, $p53^{wt/wt}$ (**Figure S2D**). However, the experimental data reveal a progressive increase in the area occupied by both $p53^{*/wt}$ basal and suprabasal cells (**Figures 3D and 4F**), which indicates that the mutant population is not in homeostasis.

In principle, one could try to explain this new scenario under the hypothesis of a single-progenitor model in balanced mode (1) in which some of the parameters have changed, leading to a transient period of growth before approaching a new stationary state.

Within the scenario of a balanced fate model, a reduction in stratification rate or an increase in division rate would lead to an increase in the basal population. This would proceed with an increase in the number of differentiated B cells n_B, as the A-cell population n_A remains constant, on average, in a balanced system. We would therefore expect to see a reduction in ρ as the proportion of B cells increases. This would mean a reduction in ρ to less than 0.03 given the increase in mutant population from <1% to 30% of the basal layer by 15 months. However, the fraction of EdU+ labelled basal cells is roughly constant and similar to wild type (**Table S3; Figure 3G**), and furthermore the proportions observed (~1.5%) appear incompatible with such a small value of ρ.

We would also expect to see a sublinear growth of the basal mutant population. Under the balanced model, new B cells are formed at a rate $\lambda^* n_A$ and stratify at a rate $\Gamma^* n_B$:

$$\frac{dn_B}{dt} = \lambda n_A - \Gamma n_B \qquad (3)$$

As $p53^{*/wt}$ suprabasal cells are observed (**Figure 4B and 4D**), we know that Γ > 0. Since the production rate is constant (fixed n_A), the growth rate dn_B/dt will decrease following an increase in the B cell population n_B, leading to a sublinear growth. Eventually the population would approach a new homeostatic state where production equals loss. In contrast to the expected balanced behavior, up to 6 months the mutant subpopulation can be seen to grow superlinearly from an initial small population, to ~10 % of the basal compartment (**Figure 3D**).

The previous features lead us to exclude the balanced mode as a model for the mutant dynamics. It follows, as we show below, that the data is consistent with a model including a cell fate imbalance:

$$A \xrightarrow{\lambda} \begin{cases} A + A & \text{Prob. } r(1+\Delta) \\ A + B & \text{Prob. } 1 - 2r \\ B + B & \text{Prob. } r(1-\Delta) \end{cases} \tag{4}$$

This model has been used previously to describe the clonal expansion of mutant epithelial progenitors(Klein et al., 2010) and can be described (in global, deterministic terms) by the following set of ODEs, whose solutions adopt an exponential shape:

$$\begin{aligned} \frac{dn_A}{dt} &= 2\Delta r\lambda n_A \\ \frac{dn_B}{dt} &= \lambda n_A - 2\Delta r\lambda n_A - \Gamma n_B \\ \frac{dn_C}{dt} &= \Gamma n_B - \mu n_C \end{aligned} \tag{5}$$

In this imbalanced case, there is an additional parameter and less available data to use for fitting than for the wild type fitting (note that *p53**/wt* clones converge and become fused after 3 months post-induction). We will therefore aim only to provide some simple explanations for the global dynamics and to show that there exist at least some parameter values for which the unbalanced model is consistent with the early stages of the *p53**/wt* mutant growth.

For the sake of simplicity, we have taken the parameter values from the wild type MLE $\hat{\theta}_{MLE}$ and we have made a minimum adjustment with the addition of a positive Δ. The small number of *p53**/wt* floating clones at the 12 week time point suggests that Δ cannot be equal to one. Based on these assumptions, we ran multiple stochastic simulations using Gillespie's Monte-Carlo algorithm with the following set of parameters:

λ = 1.16 week^{-1} r = 0.06 Γ = 3.9 week^{-1} Δ = 0.95

The results are shown in **Figures S6A and S6B**, where the simulation outcome is overlaid on the experimental data with reasonable fits, both in terms of average mutant cell populations as well as clone size distributions. In addition, one can retrieve the steady-state value of ρ from the solutions of the ODEs (5). This value (ρ^{ss} = 0.80) is similar to the wild type estimate (ρ = 0.77), in agreement with the similarity in the observed EdU data (**Table S4; Figure 3G**). While we do not claim that these are the best-fitting parameters, the consistency of the simulated results with the observed data suggests that the imbalanced fate model (4) is a plausible model to describe the first 6 months of *p53**/wt* growth.

In the case under UV irradiation, there is a large increase in the rate of $p53^{*/wt}$ colonization (**Figures 6B and 6F**). This could be explained as a result of rise in the cellular turnover (**Figures 6C and 6D**). Notice that an increase in λ would accelerate the expansion of $p53^{*/wt}$ clones according to the equations (5).

1.4 Dynamics of mutant suprabasal cells

The percentage of projected area labelled also showed a supralinear increase over the first 24 weeks post-induction (**Figure 4F**). However, this represented up to 70-80% coverage after 24 weeks in contrast with the value of 10-15% GFP+ cells reached in the basal layer. Although the projected area may partially depend on the degree of vertical stacking and lateral movement of the clonal cells, this apparent mismatch suggests an over-accumulation of the $p53^{*/wt}$ cells in the suprabasal layers. This is further supported by the increase in the epidermal thickness (**Figures 4I and 4J**) and the doubling of the keratinocyte density in the first suprabasal layer by 24 weeks (**Figures 4G and 4H**), compared with the modest increase in basal cell density (around 10%, **Figure 3F**).

To evaluate some simple possible explanations that could lead to this scenario we proceeded to numerically solve the set of ODEs that describe the unbalanced-system dynamics (5) under different hypotheses in which not only Δ but the value of other parameters of the model is allowed to vary from its configuration for the WT ($p53^{wt/wt}$). Only λ was left unaltered consistent with EdU data (**Figure 3G**). Setting an initial condition of 1% $p53^{*/wt}$ proliferating cells (in the range of the labelling induction efficiency), we tracked for each case the simulated time courses in the proportion of mutant basal population, the proportion of mutant suprabasal population, as well as the derived changes in ρ, *m*, and thickness, and compared these to the experimental observations at 6 months (**Figure S6D**). Note that simulated values for tissue thickness were estimated by converting the non-spatial cell populations into volumes using average basal and suprabasal cell sizes measured from rendered confocal z stacks – **Figures 4J and S6C**).

We observe that a large value for Δ, i.e. highly unbalanced stochastic division fates, was sufficient to explain the given changes in the basal compartment, but could only, by itself, explain a modest increase in the proportion of $p53^{*/wt}$ cells in the suprabasal layer (up to

20-30% by 6 months at most, with little change in tissue thickness). Similarly, the suprabasal fraction would not be significantly altered if there was also an increase in the rate of stratification (value of Γ) and thickness would be largerly unaffected. In contrast, small changes in *r* could dramatically alter the proportions of *p53*/wt* cells both in the basal and the suprabasal layers during the 6-months period, but the suprabasal-to-basal cell ratio indicates that mutant expansion would proceed in that case with a bigger relative cell accumulation in the basal compartment; hence, hard to reconcile with the measurements reporting just small changes in basal cell density (**Figure 3F**). We find that a large reduction in the shedding rate μ is the simplest plausible scenario. For instance, when setting values for μ 1-2 orders of magnitude smaller than in WT in conjunction with a large value of Δ (keeping other parameters as in WT), predictions are compatible with the experimental data not only in terms of the magnitude of growth in the proportion of *p53*/wt* cells in the basal layer, but also in terms of the mutant over-crowdedness in the suprabasal compartment. Furthermore, the moderate increase in thickness is also explained (**Figure S6D**).

1.5 Clonal competition during ongoing mutagenesis

Here we consider the behavior of *p53*/wt* cells induced at low frequency in mouse epidermis treated with the mutagen dimethylbenzanthracene and exposed to low levels of UV irradiation over a prolonged period. In the experiment, the area of IFE occupied *p53*/wt* cells increased before decreasing (**Figures 7C and 7D**). Detailed resolution of the multiple cellular and molecular mechanisms that may underpin the growth and decline of *p53*/wt* cells in this experiment is beyond the scope of this paper. However, we hypothesized that this behavior could be a consequence of competition between *p53*/wt* cells and cells carrying newly acquired mutations.

UV irradiation may represent a new selective pressure and cause an ongoing process of mutagenesis. New mutations occur at random throughout the UV irradiated epidermis (**Figure 7G**). Some of these can convey a competitive advantage over wild type cells and form expanding clones. At early times after induction, the *p53*/wt* cells are surrounded by wild type cells over which they have a proliferative advantage and the *p53*/wt* area grows, but the majority of the new mutations will occur within the wild type cells, since they occupy most of the tissue. Once expanding clones come into contact with each other, the

clones may compete, and the fitter clone may displace the weaker (**Figure 7E**). Most clones with a competitive advantage over wild type cells would eventually come into contact with a stronger clone, in which case we may expect to see a pattern of initial growth followed by decline, as shown in the experiments. Note that different factors may influence the competitive advantage of a clone at this stage, which may include its proliferation rate but also resilience to mechanical stress (cell crowdedness), etc.

To test whether the induction of new mutations could provide an explanation for the growth and decline of a specific cell population we ran simulations of clone growth under ongoing mutagenesis under very simple assumptions. The simulations track the relative populations of clones under two basic rules:

1. Clones grow or shrink depending on their fitness relative to the other clones in the simulation. The population of the weaker clones will decrease while stronger clones will expand.
2. New clones are generated at a certain rate by randomly occurring mutations and assigned a random fitness value.

We start the simulations with a large number of wild type cells and a small population of cells carrying a transgenic mutation, which has a slightly higher fitness than the wild type and is analogous to the *p53*$^{*/wt}$ in the experiment.

In a first implementation, populations were simulated as continuous variables and were updated by multiplying their size by their fitness to get new population sizes; then these populations were re-normalized to keep the total population constant. In each generation, there is a constant probability of a single new mutation occurring. Following this method, we tracked the descendants of the initial transgenic cells (**Figure S7E**) and also followed the progress of all clones in the simulation (**Figure 7F**). An animation of the simulation results from **Figure 7F** is shown in the **Supplemental Movie**. This animation takes the clone sizes from a *non-spatial* simulation and displays them on a 2D grid for visual purposes (the spatial behavior should not be considered meaningful). Altogether, the results shown in **Figure 7F** suggest that simulations of the basic rules above are capable of reproducing the growth and decline behavior observed in the experiment.

To confirm that these qualitative results are indeed a consequence of the basic rules rather than the exact implementation method, we applied another, independent, algorithm for simulation. This second algorithm is based on a Moran process (Moran, 1957). In this method, there is a finite population of N cells and at each step one cell is selected to replicate itself (the size of the clone it belongs to grows by one cell) and one cell is selected to die (the corresponding clone size decreases by one cell). The probability of selecting a cell to replicate is in proportion to its fitness; the selection of the cell to die is independent of fitness. Mutations are introduced at random at any point in between a birth-death event. We found that similar patterns of growth and decline of the transgenic descendants could be reproduced under this implementation.

Regardless of the particular implementation used, multiple simulation runs show that the decline of the transgenic population is not necessarily inevitable (**Figure S7E**). The particular parameters selected can influence the pattern of results. For example, a low mutation rate or a high initial growth rate for the transgenic cells means that the transgenic clone is more likely to take over the whole population before stronger mutations emerge to compete. We do not attempt to relate the parameters in these simulations directly to experimental results, merely to suggest that the principle of random mutation and competition *may* provide an explanation for the growth and decline of $p53^{*/wt}$ seen in the experiment.

For further details of the algorithms see the code, which is available at:
https://github.com/PHJonesGroup/Murai_etal_SI_code
