# Supplementary material for: Epidermal Tissue Adapts to Restrain Progenitors Carrying Clonal p53 Mutations
Source: Cell Stem Cell. 2018 Nov 1;23(5):687–699.e8. doi: 10.1016/j.stem.2018.08.017 (PMC6224607; doi:10.1016/j.stem.2018.08.017)
Supplement: Document S2. Article plus Supplemental Information [file mmc6.pdf]

Cell Stem Cell

Article

Epidermal Tissue Adapts to Restrain Progenitors Carrying Clonal *p53* Mutations

Graphical Abstract

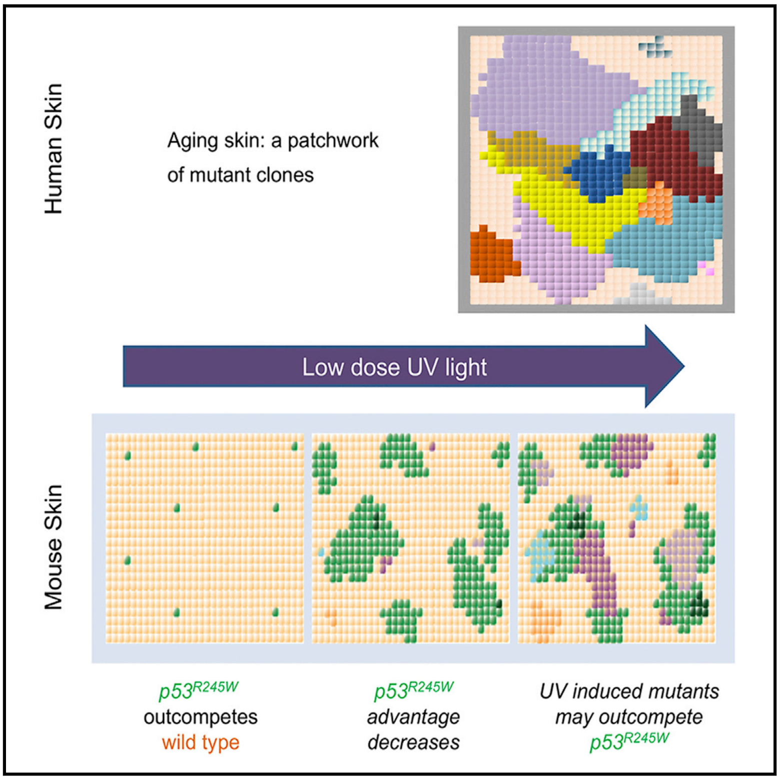

Highlights

- *p53* mutant progenitors outcompete wild-type cells to colonize normal epidermis
- The epidermis adapts to the mutant clones, slowly returning toward normal
- Low-dose UV light drives *p53* mutant clone expansion in the short term
- Prolonged UV exposure generates mutant clones that may outcompete *p53* mutant cells

Authors

Kasumi Murai, Greta Skrupskelyte, Gabriel Piedrafita, ..., Allon M. Klein, Benjamin A. Hall, Philip H. Jones

Correspondence

pj3@sanger.ac.uk

In Brief

Normal human epidermis harbors *p53* mutant clones, very few of which form tumors. Murai et al. track *p53* mutant clones as they colonize the epidermis in a transgenic mouse model showing how the tissue adapts to the mutant cells and uncovering distinct roles for physiological UV light exposure.

Murai et al., 2018, Cell Stem Cell *23*, 687–699
November 1, 2018 © 2018 The Author(s). Published by Elsevier Inc.
https://doi.org/10.1016/j.stem.2018.08.017

Epidermal Tissue Adapts to Restrain Progenitors Carrying Clonal *p53* Mutations

Kasumi Murai,[1,4] Greta Skrupskelyte,[1,4] Gabriel Piedrafita,[1] Michael Hall,[1,2] Vasiliki Kostiou,[2] Swee Hoe Ong,[1] Tibor Nagy,[1] Alex Cagan,[1] David Goulding,[1] Allon M. Klein,[3] Benjamin A. Hall,[2] and Philip H. Jones[1,2,5,*]
[1]Wellcome Sanger Institute, Hinxton CB10 1SA, UK
[2]MRC Cancer Unit, University of Cambridge, Hutchison-MRC Research Centre, Cambridge Biomedical Campus, Cambridge CB2 0XZ, UK
[3]Department of Systems Biology, Harvard Medical School, Harvard Medical School, Boston, MA 02115, USA
[4]These authors contributed equally
[5]Lead Contact
*Correspondence: pj3@sanger.ac.uk
https://doi.org/10.1016/j.stem.2018.08.017

SUMMARY

Aging human tissues, such as sun-exposed epidermis, accumulate a high burden of progenitor cells that carry oncogenic mutations. However, most progenitors carrying such mutations colonize and persist in normal tissue without forming tumors. Here, we investigated tissue-level constraints on clonal progenitor behavior by inducing a single-allele *p53* mutation (*Trp53^{R245W}; p53$^{*/wt}$*), prevalent in normal human epidermis and squamous cell carcinoma, in transgenic mouse epidermis. *p53$^{*/wt}$* progenitors initially outcompeted wild-type cells due to enhanced proliferation, but subsequently reverted toward normal dynamics and homeostasis. Physiological doses of UV light accelerated short-term expansion of *p53$^{*/wt}$* clones, but their frequency decreased with protracted irradiation, possibly due to displacement by UV-induced mutant clones with higher competitive fitness. These results suggest multiple mechanisms restrain the proliferation of *p53$^{*/wt}$* progenitors, thereby maintaining epidermal integrity.

INTRODUCTION

Proliferating human tissues harbor substantial populations of stem/progenitor cells carrying somatic mutations linked to neoplasia and other diseases (Alexandrov et al., 2015; Jaiswal et al., 2017). This process is exemplified by the epidermis, which is exposed to UV light over decades and accumulates a high proportion of progenitors carrying UV-induced oncogenic mutations under strong evolutionary selection (Martincorena et al., 2015). For example, 4%–14% of epidermal cells in sun-exposed human skin carry non-synonymous mutations of the tumor suppressor gene *p53 (TP53)* (Jonason et al., 1996; Martincorena et al., 2015). Despite this, aging epidermis remains histologically and functionally normal, and, in comparison with the size of the mutant progenitor population, the incidence of keratinocyte cancers is exceedingly low. Here, we investigate the mechanisms that constrain *p53* mutant progenitors and underpin the remarkable resilience of the epidermis to mutation.

The epidermis consists of layers of keratinocytes punctuated by hair follicles and sweat ducts (Alcolea and Jones, 2014). Keratinocytes are continually shed from the tissue surface and replaced by proliferation in the basal cell layer (Figure 1A). On commitment to terminal differentiation, proliferating basal cells exit the cell cycle and migrate into the suprabasal cell layers. They then undergo a sequence of changes in gene expression and cell morphology and are ultimately shed as anucleate cornified cells. Throughout life the epidermis self- renews, matching cell production in the basal layer with cell loss from the epidermal surface (Roshan and Jones, 2012).

Various models of normal epidermal homeostasis have been proposed (Allen and Potten, 1974; Sada et al., 2016). Multiple lineage tracing and intravital imaging studies suggest the interfollicular epidermis (IFE) is maintained by a single population of progenitor cells with stochastic fate (Clayton et al., 2007; Doupé et al., 2010; Lim et al., 2013; Rompolas et al., 2016; Roshan et al., 2016). In this paradigm, progenitor cells divide to generate two progenitor daughters, two non-dividing differentiating cells or one cell of each type (Figure 1B). The outcome of individual progenitor cell divisions is unpredictable, but the probability of generating differentiated or proliferating cells is balanced. As a result, the average cell division generates one progenitor and one differentiating daughter cell across the progenitor population, achieving cellular homeostasis and ensuring the majority of clones with mutations that do not alter cell dynamics are lost by differentiation and subsequent shedding (Figure 1B) (Clayton et al., 2007).

Epidermal progenitor fate is adaptable, enabling cells to both sustain normal cell turnover and respond to injury (Lim et al., 2013; Park et al., 2017; Roshan et al., 2016). Following wounding, nearby progenitors produce more proliferating than differentiating daughters until the epidermis is repaired, after which they revert to normal, balanced behavior (Figure 1C). While the ability to modulate progenitor cell fate provides a robust mechanism of tissue repair, it also represents a potential vulnerability, as somatic mutations may drive progenitors to produce an excess of proliferating cells in the absence of injury (Alcolea et al., 2014). Unchecked, even a small bias toward proliferation may lead to clonal dominance and eventually tumor formation (Frede et al., 2016).

This is an open access article under the CC BY license (http://creativecommons.org/licenses/by/4.0/).

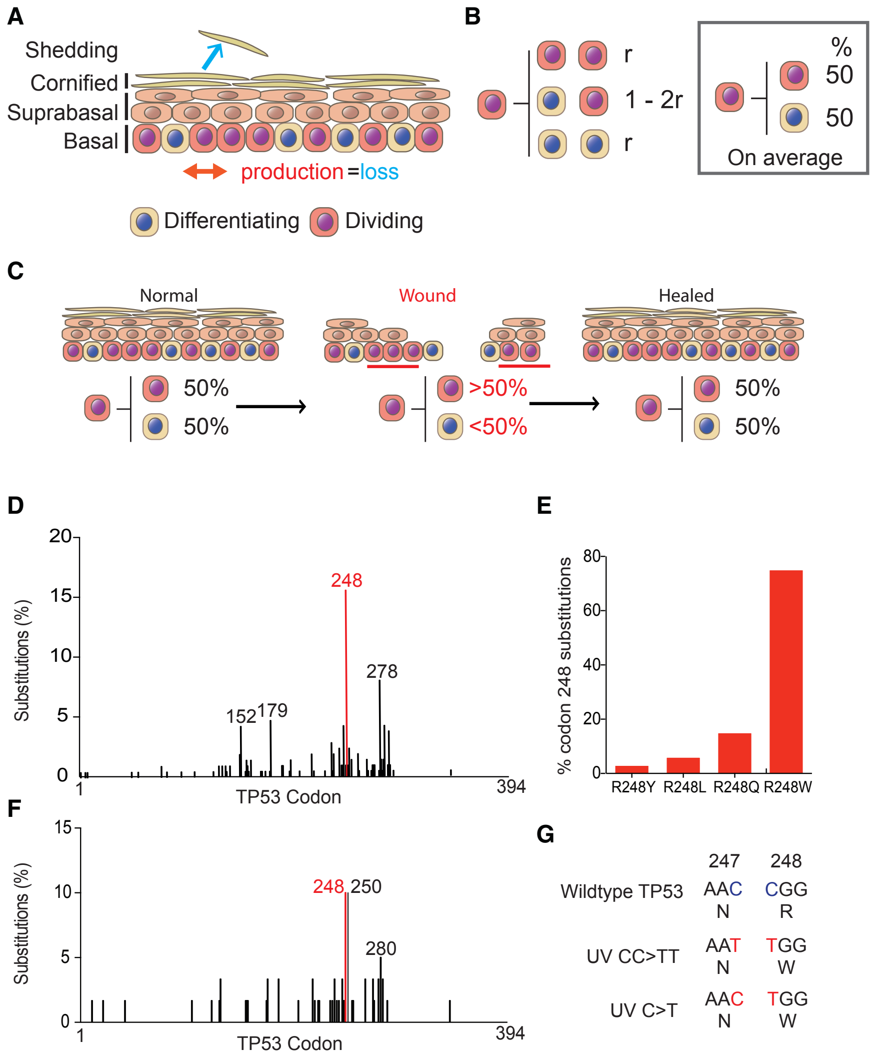

Figure 1. Cell Behavior in the Epidermis and *p53* Mutations

(A) Interfollicular epidermis (IFE). The tissue consists of layers of keratinocytes. Proliferation is confined to the basal cell layer. Differentiating basal cells exit the cell cycle and then stratify out of the basal layer, migrating through the suprabasal and cornified layers to the surface from which they are shed. In normal IFE, the rate of cell production in the basal layer (red arrow) is the same as the rate of cell loss by shedding (blue arrow).

(B) Single-progenitor model of IFE homeostasis. All dividing basal cells are functionally equivalent progenitor cells (pink). On division, a progenitor may generate two progenitors, two differentiating progeny that will cease division and stratify (beige) or one cell of each type. The outcome of a given division is unpredictable, but the likelihood (r) of producing two progenitor or two differentiating daughters is the same, so that, on average, across the population, equal proportions of progenitor and differentiating cells are generated (box).

(C) Plasticity of epidermal progenitors. Following wounding, the progenitors adjacent to the injury (red bars) switch from homeostatic behavior to producing more progenitor than differentiating progeny, until the wound is healed, and then they revert to homeostasis; numbers indicate percentages of cells generated per average cell division in each state.

(D) Distribution of TP53 missense mutations in cutaneous squamous cell carcinoma (data from COSMIC v.79, https://cancer.sanger.ac.uk/cosmic).

(E) Frequency of TP53 Codon 248 amino acid changes in cutaneous squamous cell carcinoma.

(F) Distribution of TP53 missense mutations in normal, sun-exposed human epidermis. Data from Martincorena et al., 2015.

(G) The two modes of generating $TP53^{R248W}$ codon change from UV-signature mutations.

Here, we focus on a *p53* mutant detected in normal human epidermis, $p53^{R248W}$ (Figures 1D–1G, (https://cancer.sanger.ac.uk/cosmic/) (Martincorena et al., 2015). This mutation is frequently found in human squamous cell carcinoma and has gain-of-function (GOF) properties, distinct from those of a *p53*-null allele (Muller and Vousden, 2014; Song et al., 2007). $p53^{R248W}$ is a contact mutant with altered DNA-binding properties and acts in dominant-negative manner over wild-type *p53* when they are co-expressed (Song et al., 2007). Several studies from cellular systems and humanized mouse models showed that $p53^{R248W}$ plays a role in the cancer phenotype through increased proliferation, drug resistance, migration, and chromosomal instability (Song et al., 2007; Zhu et al., 2015). However, recent studies suggest that some *p53* mutants including $p53^{R248W}$ exhibit GOF attributes only in cancer cells of particular lineages and the impact of the $p53^{R248W}$ mutation on normal cell behavior is unclear (Sabapathy, 2015). We hypothesized that $p53^{R248W}$ may alter normal progenitor behavior to increase the likelihood of clonal persistence and the acquisition of additional mutations.

To test this hypothesis, we used a mouse model. Carcinogenesis in mouse skin has been studied extensively, and, like humans, mice may harbor mutated cells long term within normal epidermis (Berenblum and Shubik, 1949; Zhang et al., 2001). We developed a transgenic strain to track the fate of individual epidermal progenitor cells following induction of $Trp53^{R245W}$, the murine equivalent of human $TP53^{R248W}$, in a background of wild-type cells, and the impact of physiological doses of UV light on mutant cell behavior.

RESULTS

Wild-Type Progenitor Behavior in Dorsal Epidermis

We began by characterizing the behavior of *p53* wild-type ($p53^{wt/wt}$) progenitors in dorsal murine IFE labeled with a neutral genetic reporter by the $Ahcre^{ERT}$-inducible *cre* recombinase line. A conditional Yellow Fluorescent Protein (YFP) allele was induced at a low frequency in basal cells of $Ahcre^{ERT}$ $Rosa26^{flYFP/wt}$ (*R-YFP*) mice (Clayton et al., 2007; Kemp et al., 2004) (Figures S1A, S2A, and S2B). Animals were sacrificed at different times and the number and location of cells in YFP-expressing (YFP^{+}) clones determined by confocal imaging (Figures S2A–S2C) (Clayton et al., 2007; Doupé et al., 2010; Page et al.,

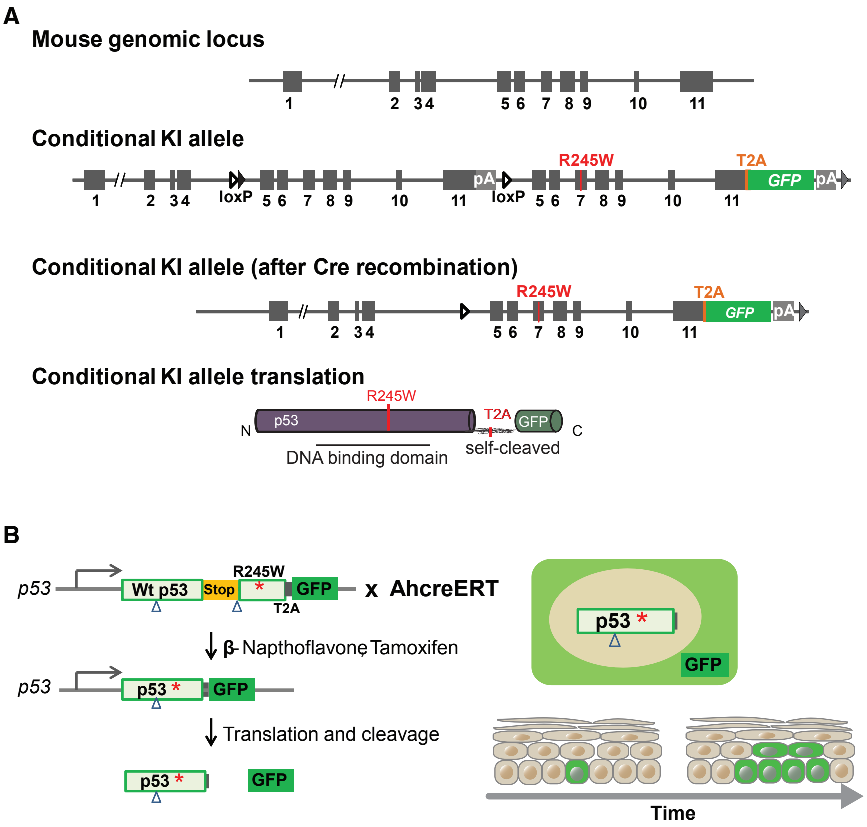

Figure 2. Conditional *p53* Mutant Transgenic Mouse Model

(A) Schematic illustration of targeting construct. *Trp53* exons 5–11 were flanked by *loxP* sites (triangle). pA indicates an additional transcriptional STOP cassette. The engineered *Trp53* duplicate region contains R245W mutations in exon 7, a C-terminal in-frame self-cleaving T2A peptide and enhanced GFP (eGFP). The conditional knockin (cKI) *Trp53* allele was obtained after *Flp*-mediated deletion of the selection markers. Closed triangle and gray triangle indicate *FRT* site and *F3* site, respectively. Prior to induction, the cKI allele expresses the wild-type *Trp53* protein; however, after *cre*-mediated recombination, the allele co-expresses $Trp53^{R245W}$ mutant protein and eGFP.

(B) Genetic lineage tracing in $Ahcre^{ERT}$ $p53^{*/wt}$ mice. *Cre*-mediated recombination, induced by β-napthoflavone (βNF) and tamoxifen (Tam), results in expression of the mutant protein in place of the wild-type. The heritable expression of the mutant protein and GFP reporter allows the study of competition between mutant clones and the surrounding wt cells.

See also Figures S4 and S5 and Table S1.

2013). Perifollicular clones adjacent to hair follicle openings were excluded. The clonal data displayed the quantitative hallmarks of a single population of progenitors with stochastic fate (Figures S2D–S2H; STAR Methods) (Clayton et al., 2007; Doupé et al., 2010; Klein et al., 2007).

To further explore the kinetics of keratinocyte turnover, we used a transgenic cell proliferation assay based on H2BGFP dilution over a period of up to 24 weeks (Figures S1B and S2I) (Doupé et al., 2012; Sada et al., 2016). Keratinocytes showed a constant and homogeneous dilution pattern in a timescale of days. These data indicated that cycling keratinocytes divided at a similar rate (Figures S2K–S2M; STAR Methods), arguing that growth arrest and senescence, two of the stress responses mediated by *p53*, are rare in wild-type IFE (Figure S2J) (Muller and Vousden, 2014). Altogether, both lineage tracing and cell proliferation data were consistent with dorsal IFE being maintained by a single population of progenitors with balanced stochastic fate (Figures 1B and S3; STAR Methods).

Inducible $p53^{R245W/wt}$ Transgenic Mice

To determine whether induction of a heterozygous $p53^{R245W}$ allele altered progenitor cell behavior, we developed a new conditional mouse strain, $Trp53^{fl\text{-}R245W\text{-}GFP/wt}$ (henceforth referred to as $p53^{*/wt}$). A conditional allele of *p53**, with a GFP reporter linked to the C terminus of the p53* protein by a T2A self-cleaving peptide, was targeted to the *p53* locus (Figures 2A and S4) (Trichas et al., 2008). This design allowed us to track individual $p53^{*/wt}$ cells in a $p53^{wt/wt}$ background. We confirmed that the p53* protein had properties similar to those of the human $p53^{R248W}$ protein and observed perturbation of p53 targets and differentiation genes in cultured keratinocytes consistent with previous reports (Freije et al., 2014; Song et al., 2007; Truong et al., 2006) (Figures S4 and S5). Following tag cleavage, a 20-amino-acid peptide remained at the C terminus of p53* protein. To test whether this peptide altered the properties of the p53* protein, we generated a second conditional mouse strain with an untagged $p53^{R245W}$ mutant allele. RNA sequencing (RNA-seq) analysis of recombined primary cultures of $p53^{*/*}$ and the untagged $p53^{R245W/R245W}$ keratinocytes revealed minimal differences in transcription (Figure S5J; Table S1).

$p53^{*/wt}$ Mutant Progenitor Cells Colonize Wild-Type Epidermis

To track the fate of $p53^{*/wt}$ progenitors *in vivo*, we bred $Ahcre^{ERT}p53^{*/wt}$ mice and induced recombination in individual cells in adult mice (2B and Figures 3A). $p53^{*/wt}$-expressing clones in IFE whole mounts were imaged by confocal microscopy (Figures 3B and 3C). In contrast to control R-YFP mice, in which the area occupied by YFP^+ basal cells remained approximately constant after induction, the proportion of $p53^{*/wt}$ basal cells rose progressively indicating the mutant population had a competitive advantage over their wild-type neighbors (Figures 3D and S2D). The number of basal cells/clone was consistently higher in $p53^{*/wt}$ than in YFP^+, $p53^{wt/wt}$ clones at the same time point ($p < 0.0001$ at 1.5, 3, 6, and 12 weeks, two-tailed Mann-Whitney test, Figure 3E). By 24 weeks, the $p53^{*/wt}$ clones had expanded so much that they had fused, but we noted a modest but statistically significant increase in the basal cell density (cells/area, $p = 0.0007$ by two-tailed Mann-Whitney test) (Figures 3C, 3D, and 3F). We concluded that following induction $p53^{*/wt}$ cells

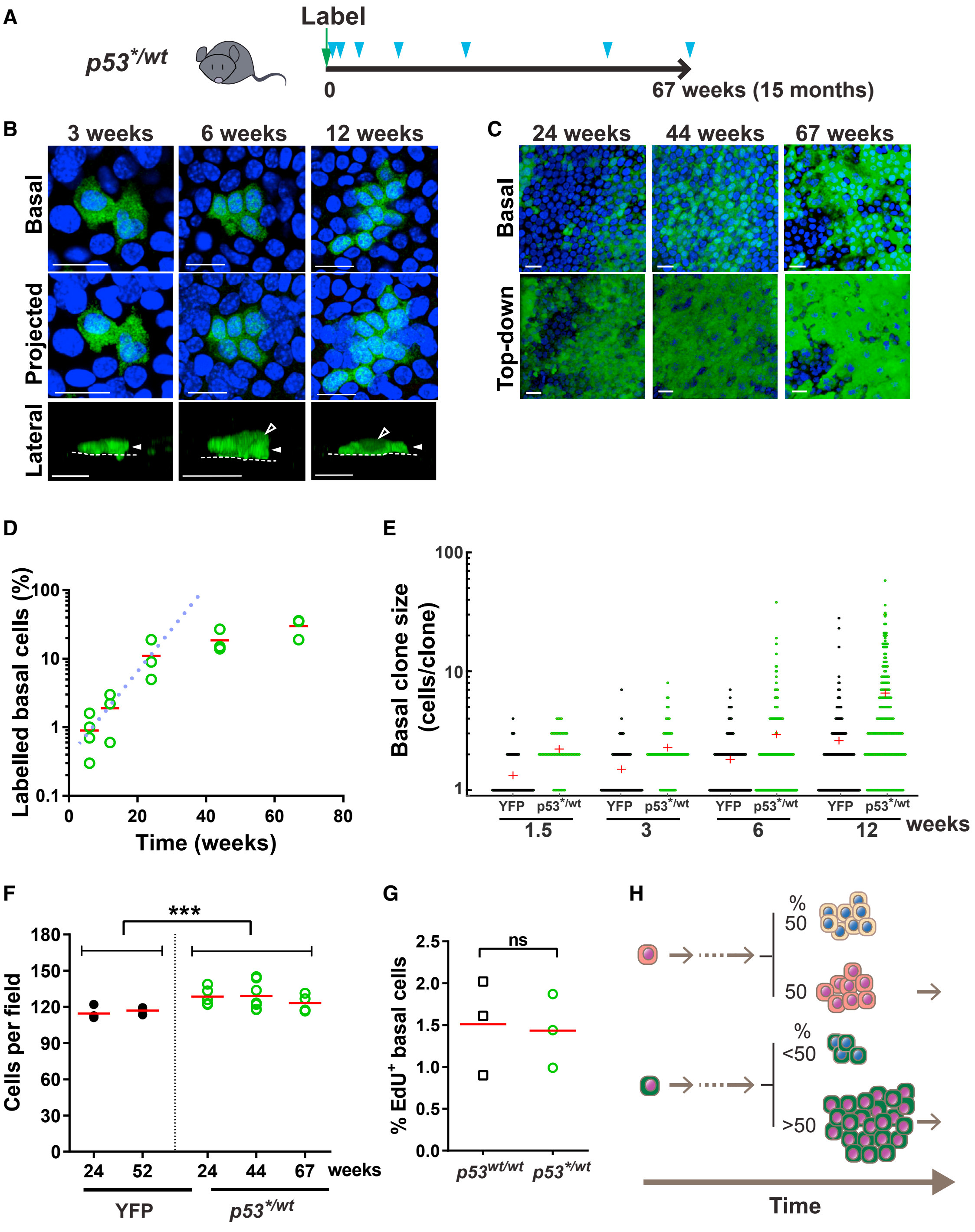

Figure 3. Heterozygous $p53^{R245W}$ ($p53^{*/wt}$) Mutant Cell Fate

(A) Protocol: clonal labeling of *Ahcre*^{ERT}*p53*$^{flR245W/wt}$ (*p53*$^{*/wt}$) mice followed by sampling (triangles).

(B) Rendered confocal z stacks showing typical *p53*$^{*/wt}$ clones in back skin epidermis. Basal, top-down view of basal layer; projected, top-down view through all nucleated cell layers; lateral, side view. Cells in basal and first suprabasal cell layers are indicated by closed and open arrowheads, respectively; dotted line, basement membrane. Green, GFP; blue, DAPI. Scale bars, 20 μm.

(C) Rendered z stacks showing basal and top-down views of typical whole mounts at times indicated. Green, GFP; blue, DAPI. Scale bars, 20 μm.

(D) Proportion of labeled basal cells at indicated time points. Averaged value from 4–5 fields per animal, n = 3 animals per time point except n = 4 animals at the 6-week time point. Red lines, mean value. Dotted line indicates the expected growth of *p53*$^{*/wt}$ basal cells clone area if the rate of expansion is constant.

(E) Clone size distributions (basal cells per clone). Red cross indicates mean clone size. Data are from 3–5 mice per time point. For *p53*$^{*/wt}$ and *RYFP*, respectively, n = 50, 93 clones at 1.5 weeks, 75, 106 clones at 3 weeks, 199, 181 clones at 6 weeks and 196, 183 clones at 12 weeks.

(legend continued on next page)

are dominant over wild-type keratinocytes, leading to colonization of the IFE.

Next, we investigated the cellular mechanism(s) that underpinned the competitive advantage of *p53*$^{*/wt}$ progenitors. Heightened resistance to the canonical *p53* stress responses in the mutant cells seemed unlikely, as apoptosis was hardly detectable (0%–0.5%) and growth arrested/senescent wild-type keratinocytes were not detected in wild-type epidermis (Figure S2J; Table S2) (Clayton et al., 2007; Doupé et al., 2010). The proportion of mutant and wild-type basal cells staining for 5-ethynyl-2′-deoxyuridine (EdU), a marker of the S phase of the cell cycle, was similar, arguing that the cell-cycle time and the proportion of progenitors were not substantially altered by the mutation (Figure 3G). Within the single-progenitor paradigm, a further candidate explanation for the competitive advantage of *p53*$^{*/wt}$ progenitor cells was a statistical bias in the fate of mutant progenitors, leading to an excess of dividing over differentiating daughters per average cell division. Such a bias would result in an increase in the proportion of *p53*$^{*/wt}$ progenitors in the IFE at each successive round of cell division, even if the rate of mutant cell division was the same as that of wild-type cells (Figure 3H). Quantitative analysis showed that up to 3 months post induction the observed behavior of *p53*$^{*/wt}$ clones was indeed consistent with such a model, with a marked excess of divisions generating two progenitor daughters over two differentiating daughters (Figures S6A and S6B; STAR Methods).

Strikingly, beyond 24 weeks post induction, the rate of expansion of the *p53*$^{*/wt}$ population slowed substantially (Figure 3D). Yet, EdU data at later time points suggest that this was not due to a decrease in the cell-division rate (Table S3). This argues that the cell-fate imbalance of mutant progenitors is not cell autonomous but responds to changes in the cellular environment, such as the observed increase in basal cell density by 24 weeks (Figure 3F) (Miroshnikova et al., 2018; Roshan et al., 2016). Also, since the proportion of mutant cells adjacent to wild-type cells will decrease as the area of mutant epidermis rises, any imbalance in fate driven by competition between mutant and wild-type will inevitably decline over time. The lack of long-term clonal level data precluded further analysis to resolve between these mechanisms. However, it is clear that the reversion of *p53*$^{*/wt}$ cell dynamics toward normal contributed to the ability of normal tissue to tolerate mutant cells.

p53$^{*/wt}$ Perturbs Keratinocyte Differentiation and Shedding

Motivated by previous reports linking *p53* with keratinocyte differentiation, we investigated the behavior of differentiating *p53*$^{*/wt}$ cells (Freije et al., 2014; Truong et al., 2006). As in wild-type IFE, mutant cell proliferation was confined to the basal layer (Figure 4A). 3D imaging of *p53*$^{*/wt}$ clones revealed that GFP expression was restricted to the basal and first suprabasal cell layers. This argued that mutant *p53* was not transcribed above the lowest epidermal cell layers and/or that *p53*$^{*/wt}$ cells failed to complete the differentiation program and reach the upper layers of the epidermis (Figures 4B and 4C). To resolve between these possibilities, we generated *Ahcre*^{ERT}*p53*$^{*/wt}$*Rosa26*$^{Confetti/wt}$ mice carrying a conditional multicolor “Confetti” allele targeted to the Rosa26 locus (Figure 4D) (Snippert et al., 2010). Imaging of clones expressing both red fluorescent protein and GFPs revealed *p53*$^{*/wt}$ cells were indeed capable of terminal differentiation into cornified layer cells (Figures 4D and 4E).

Defects in keratinocyte differentiation may have a major impact on epidermal function and promote carcinogenesis, motivating us to examine the behavior of differentiating mutant cells more closely (Darido et al., 2016). Over the first 6 months after induction, the area occupied by GFP-expressing mutant suprabasal cells rose even faster than the area occupied by mutant basal cells, arguing differentiating mutant cells accumulate in the tissue compared with their wild-type counterparts (Figures 4F and 3D). In keeping with this hypothesis, the thickness of the IFE and the density of cells in the first suprabasal layer increased substantially associated with changes in cell morphology, signs of cellular disorganization and extra cell layer(s) (Figures 4G–4J and S6C).

In order to explore possible mechanisms that could lead to this scenario, we implemented computational simulations of the cell-population dynamics and tissue structure under various hypotheses incorporating parameters measured from the epidermis (STAR Methods). Briefly, we considered changes in either the proportion of symmetric divisions, stratification rate, or shedding rate along with the extent of cell-fate imbalance. Predicted changes in the proportion of *p53*$^{*/wt}$ cells in basal/suprabasal layers and tissue thickness were compared against experimental observations. Only *p53*$^{*/wt}$ progenitor cell-fate imbalance accompanied by a substantial reduction in the shedding rate was able to reproduce the patterns seen, arguing this was the most likely explanation (Figure S6D; STAR Methods). Strikingly, however, beyond 6 months, the area occupied by mutant suprabasal cells remained approximately constant while epidermal thickness decreased, arguing that mutant cell differentiation and shedding had been at least partially restored. This slow adaptation of mutant differentiating cell dynamics resulted in the preservation of tissue integrity without the development of epidermal tumors (Table S4).

The mismatch between the proportion of mutant basal and suprabasal cells has implications for cell movement within the epidermis. In normal IFE, the majority of differentiating cells stratify vertically through the suprabasal cell layers (Figure 5A) (Doupé et al., 2010; Rompolas et al., 2016). In contrast, in *p53*$^{*/wt}$ IFE, most mutant suprabasal cells overlie wild-type basal cells, indicating extensive lateral displacement has occurred

(F) Mean basal cell density in *p53*$^{*/wt}$ and *RYFP* control mice. Data are means of 5 fields per mouse. n $\geq$ 4 mice at each time point/group. Exact sample numbers are in corresponding supplementary table, ***p = 0.0007 by Mann-Whitney test.

(G) Average percentage of EdU-labeled basal cells in *p53*$^{*/wt}$ clones at the 12-week time point compared to non-labeled cells (*p53*$^{wt/wt}$) in same mouse. n = 3 mice. ns, no significant difference by paired t test.

(H) Schematic illustration of *p53*$^{*/wt}$ cell behavior. A bias in the fate of *p53*$^{*/wt}$ cell can result in an increase in the proportion of *p53*$^{*/wt}$ progenitors in the IFE, even if the rate of mutant cell division was the same as that of wild-type cells.

See also Figure S1–S6 and Tables S2 and S7.

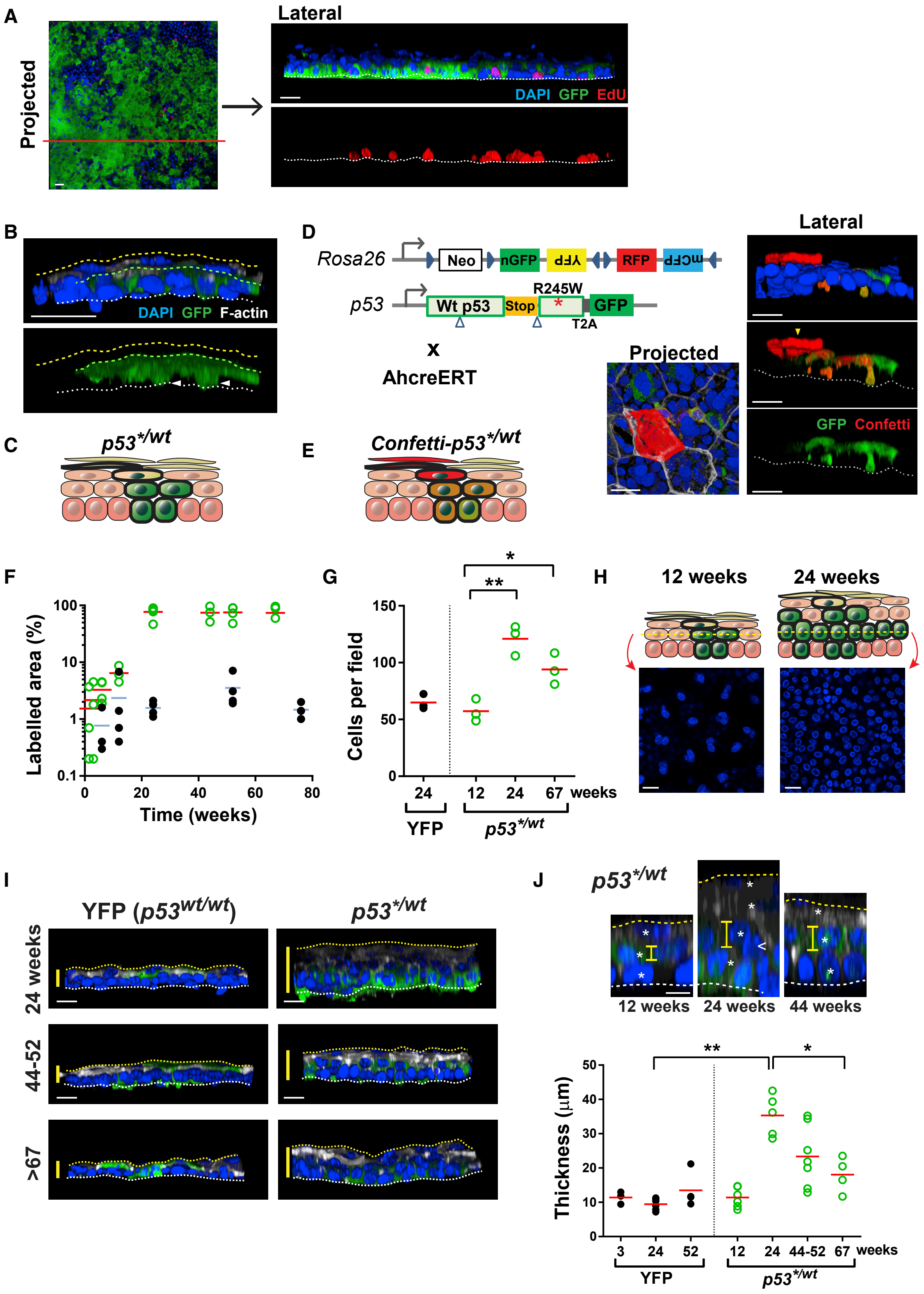

Figure 4. Behavior of Differentiating $p53^{*/wt}$ Keratinocytes

(A) Rendered confocal z stacks showing $p53^{*/wt}$ clone area. Lateral view shows the section of projected image (red line). White line, basement membrane; blue, DAPI; green, GFP; red, EdU. Scale bars, 20 μm.

(legend continued on next page)

(Figure 5A). We hypothesized that there may be alterations in cell-cell adhesion permissive of lateral migration in mutant IFE. Transmission electron microscopy of *p53*$^{*/wt}$ IFE at a year post induction revealed widespread disruption of desmosomes (Figures 5B and 5C) (Dusek and Attardi, 2011). Expression of the desmosomal proteins, DSG3 and CDH1, and levels of membrane-associated CTNNB1, which binds CDH1, were diminished in *p53*$^{*/wt}$ compared with adjacent wild-type areas (Figure 5D). Despite these changes, the *p53*$^{*/wt}$ epidermis retained its "outside-in" barrier function (Figures 5E and 5F). We concluded that the lateral migration of *p53*$^{*/wt}$ suprabasal cells may be facilitated by the disruption of desmosomes.

Low-Dose UV Light Exposure Drives Mutant Clone Expansion

Human epidermis is frequently exposed to low doses of UV light, below the level that causes keratinocyte apoptosis and sunburn (Jonason et al., 1996). As well as generating new mutations, repeated low doses of UV have been argued to drive the expansion of clones carrying p53 protein stabilizing mutations (Klein et al., 2010). We therefore investigated the effect of such physiological exposure.

First, we examined the impact of a short course of a low level UV-B (sub minimal erythema dose, MED, wavelengths 280–315 nm) treatment on *p53*$^{wt/wt}$ and *p53*$^{*/wt}$ progenitors *in vivo*. The UV dose was titrated to induce detectable DNA damage with minimal change in apoptosis (Figures S7A and S7B; Table S5). An area of shaved dorsal epidermis was exposed to daily doses of UV for 4 days per week. After 2 weeks, *cre* was induced and samples collected from irradiated and adjacent unexposed epidermis for up to 6 weeks (Figures 6A and 6B). In wild-type *p53*, R-YFP mice, the proportion of EdU^{+} basal cells and the mean number of cells per clone were increased in irradiated compared to unexposed epidermis, consistent with UV treatment accelerating the rate of cell division (Figures 6C, S7C, and S7D). However, the area of labeled epidermis remained approximately constant despite the increased rate of epidermal turnover (Figure 6E). We concluded that the progenitor population continued to maintain the epidermis with balanced production of progenitor and differentiating keratinocytes during low-dose UV irradiation.

In induced *p53*$^{*/wt}$ animals, UV treatment greatly accelerated IFE colonization by mutant cells (Figures 6B and 6F). However, the proportion of proliferating EdU^{+} *p53*$^{*/wt}$ basal cells in irradiated IFE was similar to that in irradiated wild-type cells (Figures 6C and 6D). As in the unexposed epidermis, these observations indicate the fate of *p53*$^{*/wt}$ progenitors is biased toward proliferation. Accelerated turnover along with a fate imbalance is predicted to lead to a faster *p53*$^{*/wt}$ colonization (Figure 6G; STAR Methods).

In humans, low-dose UV exposure continues over decades and results in a dense patchwork of epidermal clones carrying mutations in *p53* and other genes (Martincorena et al., 2015). To model this scenario, *p53*$^{*/wt}$ mice were induced and the skin given a single treatment with the mutagen dimethylbenzanthracene (DMBA) to load the epidermis with mutations (Abel et al., 2009). Dorsal epidermis was then treated with sub MED UV for up to 9 months, and exposed and adjacent unexposed areas compared (Figure 7A). Over the first 12 weeks the area occupied by GFP-labeled *p53*$^{*/wt}$ cells expanded significantly faster in the presence of UV light, as shown above (Figures 7B and 7C). However, continuing exposure resulted in a progressive decline in the *p53*$^{*/wt}$ population (Figures 7B–7D).

Given the short-term effect of UV treatment, the depletion of *p53*$^{*/wt}$ cells during long-term exposure was surprising. Several different scenarios can be envisaged to explain this observation. First, it might result from a non-cell-autonomous adaptation to a local tissue stress, e.g., such as sensitivity to increased cell density, which would also explain the restraining of *p53*$^{*/wt}$ expansion in non-irradiated epidermis. Alternatively, cell-autonomous factors, such as long-term UV-induced accumulation of cellular damage in p53$^{*/wt}$ cells or an adaptive increase in wild-type cell fitness may cause a decline in mutant competitiveness. Another possibility is competition between *p53*$^{*/wt}$ cells and mutations caused by the DMBA treatment or UV irradiation. In the last scenario, UV exposure generates *new* mutations with a range of possible effects on competitive fitness of mutant progenitors. Initially, *p53*$^{*/wt}$ cells encounter mostly wild-type cells

(B) Side view of *p53*$^{*/wt}$ clone at the 12-week time point. White line, basement membrane; yellow line, epidermal surface; green line, outer limit of GFP-expressing cells. Green, GFP; blue, DAPI; white, F-actin. Scale bar, 20 μm, arrowheads, basal layer cells.

(C) Schematic: typical *p53*$^{*/wt}$ clone with GFP expression (green) confined to basal and first suprabasal cell layers.

(D) Multicolor lineage tracing in *Ahcre*^{ERT}*Rosa26*$^{flConfetti/wt}$*p53*$^{*/wt}$ animals. Left: the multicolor confetti reporter allele encodes four fluorescent proteins. Different *cre*-mediated inversion and excision recombination events result in the heritable expression of one of the four fluorescent proteins depicted, nuclear GFP, yellow fluorescent protein (YFP) red fluorescent protein (RFP), or membrane cyan fluorescent protein (CFP). The fate of *p53* mutant progenitors can be tracked in double-labeled clones even if the *p53* locus becomes inactive in the differentiating progeny. Right: rendered z stacks of typical *Confetti-p53*$^{*/wt}$ double-labeled clone 12 weeks post induction of *Ahcre*^{ERT}*Rosa26*^{Confetti}*p53*$^{*/wt}$ mice. Images representative of 8 clones. Yellow arrowhead, cornified cell. Scale bars, 20 μm.

(E) Schematic: RFP expression reveals differentiation of *p53*$^{*/wt}$ clone.

(F) Projected area of labeled epidermis (%) in induced *p53*$^{*/wt}$ (open green circles) and *p53*$^{wt/wt}$ *RYFP* (solid black circles) mice. Data are from 5 fields per animal. n = 3–5 mice per time point/group. Red and blue lines, mean area for *p53*$^{*/wt}$ and *p53*$^{wt/wt}$ *RYFP*, respectively.

(G) Mean cell density in immediate suprabasal layer. Data are means of 5 fields per mouse. n = 3 mice at each time point/group except 4 fields from 1 animal are shown for YFP. *p = 0.0236, **p = 0.0035 by two-tailed unpaired t test.

(H) Single z-slice image of immediate suprabasal layer from *p53*$^{*/wt}$ mice at indicated time point. Schematic: typical *p53*$^{*/wt}$ colonized area at indicated time point. Scale bar, 20 μm.

(I) Rendered confocal z stacks showing side view of epidermal whole mounts. Yellow bars indicate the thickness of epidermis. Green, GFP; blue, DAPI; white, F-actin. Scale bar, 20 μm. White line, basement membrane; yellow line, epidermal surface.

(J) Detail analysis of change in epidermal thickness. Top, confocal images (side view) showing changes in *p53*$^{*/wt}$ epidermis over time. Yellow bars, length of cells in first suprabasal layer; asterisks, each cell layers. Arrowhead indicates cell in between layers. Scale bar, 20 μm. Bottom, mean thickness of epidermis at time points indicated. n ≥ 3 mice at each time point/group. *p = 0.016, **p = 0.0043 by Mann-Whitney test.

See also Figure S6 and Table S2–S4 and S7.

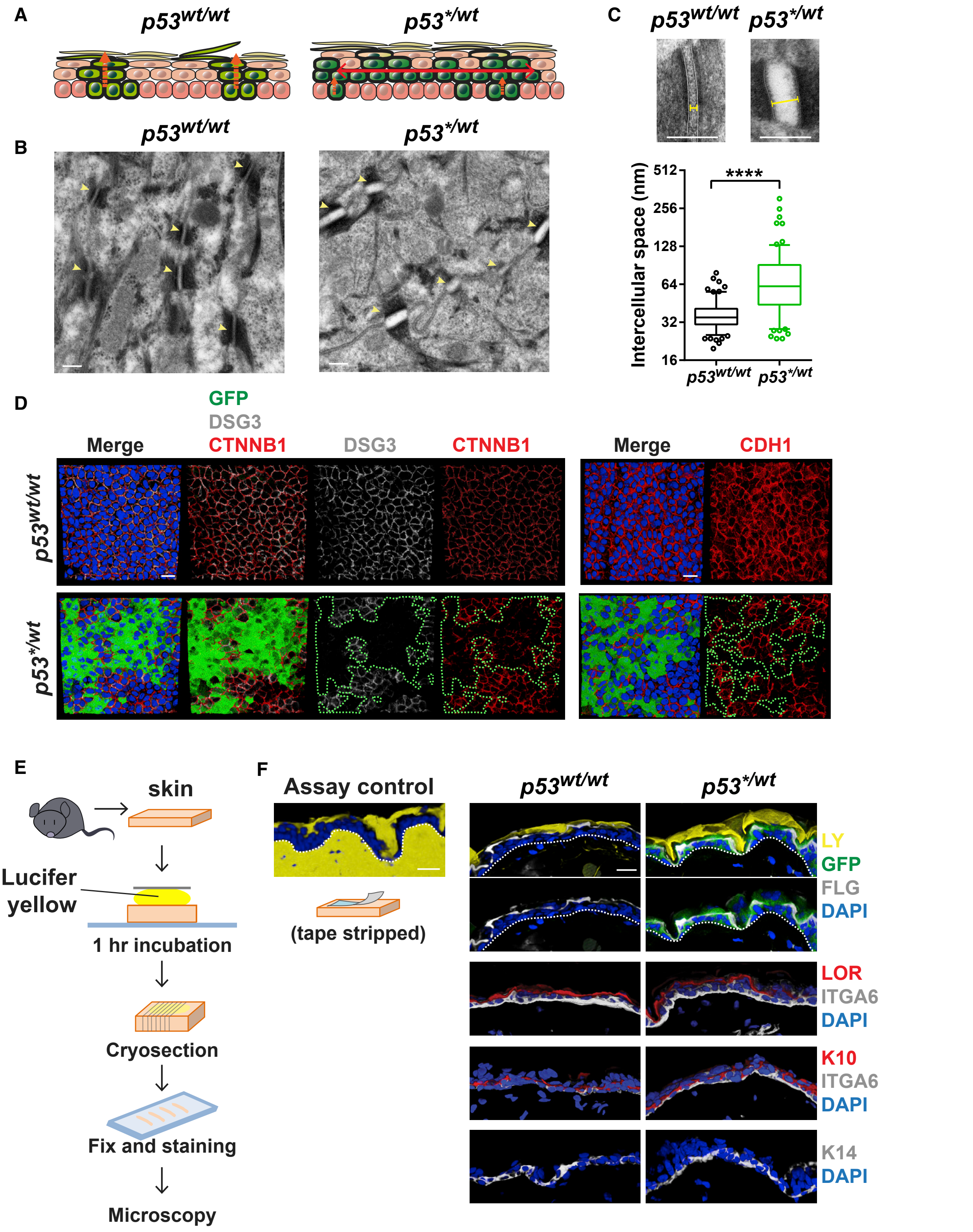

Figure 5. Disrupted Cell Adhesion in $p53^{*/wt}$ Epidermis at 1 Year post Induction

(A) Schematic: movement of differentiating cells is predominantly vertical in $p53^{wt/wt}$ mice but the excess of suprabasal over basal cells in $p53^{*/wt}$ IFE argues there is extensive lateral displacement of mutant cells.

(B–F) Analysis of epidermis colonized by $p53^{*/wt}$ at 1 year post induction. Uninduced mice were used as control. n = 3 mice per group.

(legend continued on next page)

over which they are dominant, resulting in progressive expansion of the $p53^{*/wt}$ population. However, as the experiment proceeds, DMBA- or UV-induced mutant clones fitter than wild-type cells will expand and eventually collide with each other and with $p53^{*/wt}$ cells. At this point, the less-fit cells would be displaced from the tissue (Figure 7E). Simple simulations embodying this hypothesis suggest such competition as a possible explanation for the observed changes in the $p53^{*/wt}$ population (Figures 7F and S7E; STAR Methods; Video S1). Hence, long-term UV exposure may contribute to the decrease in the $p53^{*/wt}$ population both by inducing mutations that allow cells to out-compete $p53^{*/wt}$ and by providing an environment in which other mutations have an advantage over $p53^{*/wt}$.

The cell-competition hypothesis in particular makes several testable predictions. If competition is responsible for the depletion of $p53^{*/wt}$ cells after 36 weeks, we would expect that clones carrying protein-altering mutations (possibly with the UV mutational signature) would be present in exposed epidermis and that the number and size of such clones would increase from 12 to 36 weeks of UV treatment. To test this, we used ultra-deep targeted exome sequencing on a panel of 74 genes linked with murine squamous carcinogenesis. Both induced and non-induced mice were included to check that the observed mutations were not merely passenger mutations within $p53^{*/wt}$ clones.

While no mutations were detected in epidermis exposed to UV for 12 weeks, we identified 58 mutations in epidermis exposed to UV for 36 weeks compared with 8 in adjacent unexposed skin in 7 mice ($p = 0.02$, 2-tailed Mann-Whitney test) (Figures 7G and S7F; Table S6). These mutant calls correspond with very large clones as the lower limit of detection was $\sim 0.15\ mm^2$ in area. The mutations were largely C > T nucleotide changes, consistent with the UV-light mutational signature, and the majority of mutations were non-synonymous, making it feasible for some of them to have altered cellular competitive fitness (Figures S7G and S7H). We noted recurrent cancer-associated mutations in *Trp53*, finding *R270C* mutants in UV-exposed epidermis in 4/7 mice after 36 weeks UV. The mutations found in the non-induced samples show that the expansion of new mutations does not rely on hitch-hiking within $p53^{*/wt}$ clones. Taken together, these observations are consistent with clones carrying UV- or DMBA-induced mutations displacing $p53^{*/wt}$ cells during prolonged UV exposure. In contrast, in the adjacent unexposed areas of the skin, the expansion of the $p53^{*/wt}$ population was not impeded by competition (Figures 7B and 7C).

A further prediction of the cell-competition hypothesis is that transgenic and UV-induced mutant clones must ultimately collide. One way to visualize such events is to immuno-stain for p53. This reveals cells carrying UV-induced, protein-stabilizing mutations, such as $Trp53^{R270C}$, but does not detect the low levels of wild-type p53 or mutations that do not stabilize the protein including p53* (Figure S5H) (Jonason et al., 1996). We observed an increase in the areas covered by p53 immunopositive cells from 12 to 36 weeks under UV exposure but barely detected any in adjacent un-irradiated epidermis at 36 weeks (Figures 7B and 7H). In several cases, these cells were in direct contact with $p53^{*/wt}$ regions, infiltrating underneath differentiating edges of $p53^{*/wt}$ clones (Figure 7H). We concluded that cells carrying protein-stabilizing mutations in *p53* do indeed collide with $p53^{*/wt}$ cells during prolonged UV exposure.

Altogether, these results suggest that, in addition to the phenotypic adaptation observed in the non-irradiated experiments, competition between mutant clones may be an important factor in constraining *p53* mutant clone expansion in the evolving mutational landscape of normal epidermis

DISCUSSION

Here, we show that the phenotype of epidermal progenitor cells carrying a heterozygous *p53* gain-of-function mutation adapts to alterations in the cellular environment. In mice protected from UV light, *p53** confers a strong competitive advantage over wild-type progenitor cells, as the average mutant cell division generates more dividing than differentiating progeny. In addition, retardation of terminal differentiation and shedding leads to mutant cell accumulation in the suprabasal layers. Unchecked, the combination of progenitor cell fate favoring proliferation and a decrease in cell shedding would be sufficient to generate tumors (Frede et al., 2016). However, the progressive attenuation of the mutant phenotype enables the epidermis to retain its functional integrity despite a high burden of *p53* mutant cells.

The mechanisms underpinning the adaptation of $p53^{*/wt}$ cells remain to be determined. The slowing of expansion of the $p53^{*/wt}$ basal cell population beyond 6 months post induction is accompanied by crowding of cells in the basal layer. A similar phenomenon has been observed in “imprisoned” UV-induced *p53* mutant clones and *Notch* mutant keratinocytes (Alcolea et al., 2014; Zhang et al., 2001). Keratinocyte differentiation is sensitive to mechanical forces suggesting that basal cell crowding may promote a return toward cellular homeostasis (Le et al., 2016; Roshan et al., 2016). New technologies will be required to determine the possible role of feedback effects

(B) Transmission electron microscopy of induced $p53^{*/wt}$ and uninduced $p53^{wt/wt}$ IFE. Arrows indicate desmosomes. Scale bar, 200 nm.

(C) Intercellular distance (yellow capped bar) at desmosomes in $p53^{wt/wt}$ (black) and $p53^{*/wt}$ (green) IFE. Box plots show median (line across box), 25th and 75th percentiles (box) and 5th and 95th percentiles (whiskers), dots are outliers (dots). n = 150 desmosomes from 3 animals per group. Scale bar, 200 nm. ****$p < 0.0001$ by Mann-Whitney test.

(D) Bottom-up view of rendered confocal z stacks showing the expression of cell adhesion molecules in $p53^{*/wt}$ and $p53^{wt/wt}$ IFE. Images are representative from 3 mice per genotype. White, DSG3; red, CTNNB1 (left panels), CDH1 (right panels); green, GFP; blue, DAPI. Green line, $p53^{*/wt}$ clone area. Scale bar, 20 μm.

(E) Scheme of epidermal permeability barrier function assay.

(F) Cryosections of $p53^{*/wt}$ epidermis showing normal expression of markers and barrier function compared to that of $p53^{wt/wt}$. $p53^{*/wt}$ colonized over 70% at this time point and GFP (green) indicates the expression of *p53** transcript. Lucifer yellow is excluded by a normal competent epidermal surface barrier; in the assay control, barrier was removed by tape stripping. Yellow, lucifer yellow; blue, DAPI. FLG, filaggrin; LOR, loricrin; ITGA6, integrin α6; K10, keratin 10; K14, keratin 14. Scale bar, 20 μm.

See also Table S7.

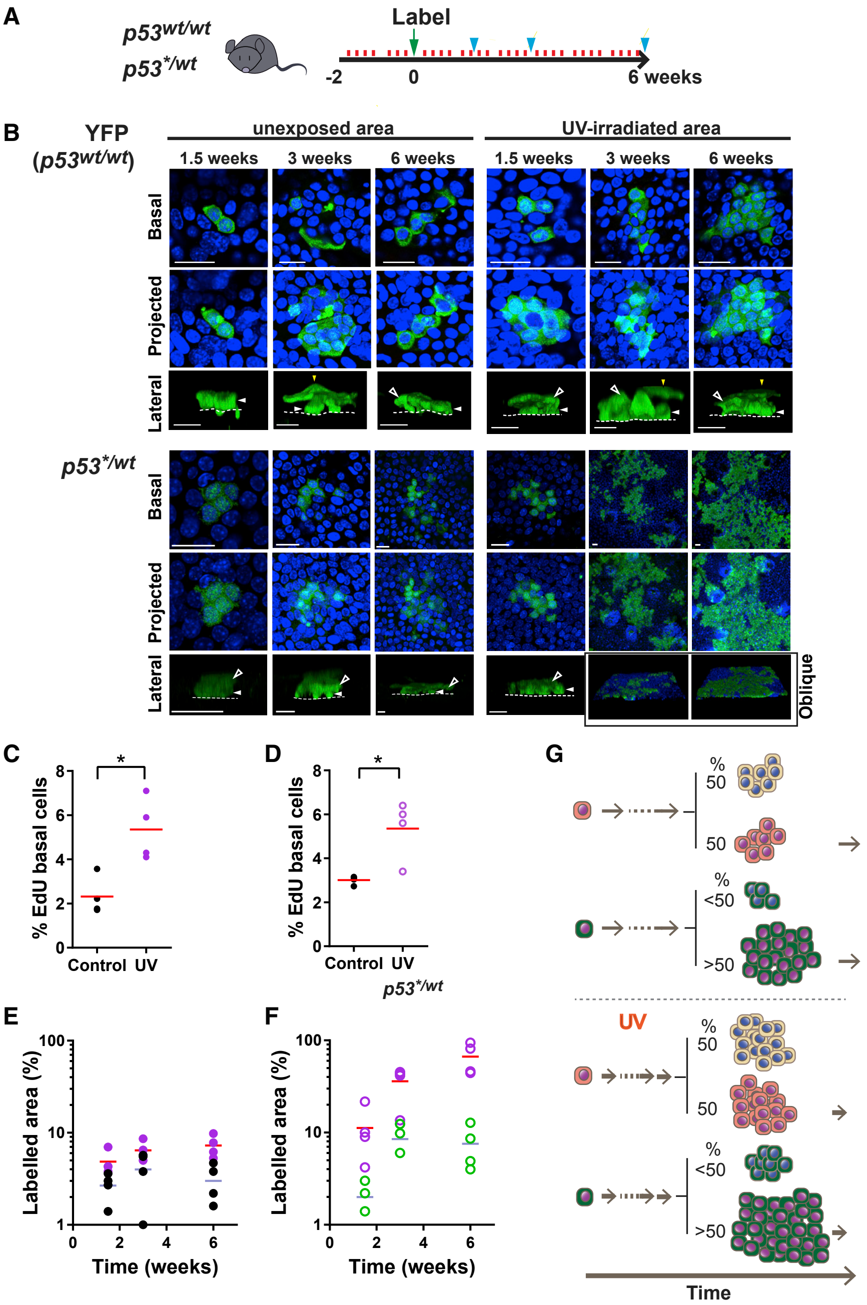

Figure 6. Short-Term Low-Dose UV Exposure Accelerates $p53^{*/wt}$ Colonization

(A) Protocol: *AhcreERT-p53*$^{*/wt}$ or *-RYFP* mice were exposed to a sub-erythema dose of UVB daily, 4 days per week for 2 weeks (red lines), after which labeling was induced (green arrow) and UV exposure continued; sample collection is indicated by blue arrows.

(B) Rendered confocal z stacks showing representative clones in epidermal whole mounts. Green indicates YFP in *YFP* panels and GFP in $p53^{*/wt}$ panels; blue, DAPI. Scale bars, 20 μm. Basal, immediate suprabasal cells and basement membrane are indicated by closed and open arrows and dotted line, respectively.

(C and D) Average percentage of EdU-labeled basal cells at the 6-week time point in induced Ah^{creERT}-$p53^{*/wt}$ (D) or Ah^{creERT}-*RYFP* (C) IFE. Samples were taken from UV-irradiated (purple circles) or adjacent unexposed areas (ctl, black circles). Values are mean from 5 fields per mouse. Red line indicates mean. n = 4 mice per condition. $*p < 0.05$ by paired t test. Percentage EdU in UV-irradiated epidermis in (D) was quantified in $p53^{*/wt}$ clone area.

(E and F) Projected area of labeled UV irradiated and adjacent unirradiated IFE. Values are percentage from 6 fields. n = 4 mice per time point. (E) *RYFP* (purple, UV; black, unirradiated). (F) $p53^{*/wt}$ (purple, UV; green, unirradiated).

(G) Schematic illustration of the effect of UV on clone behavior. UV increases the rate of cell division in both $p53^{wt/wt}$ and $p53^{*/wt}$ cells but accelerates IFE colonization by mutant cells as $p53^{*/wt}$ progenitors retain a bias in fate, generating more progenitor than differentiating daughters.

See also Figure S7 and Tables S5 and S7.

from suprabasal cell crowding on epidermal proliferation and tissue turnover.

A persistent legacy of $p53^{*/wt}$ mutation is the loss of desmosomes and downregulation of expression of CDH1 and other adhesion proteins, consistent with the observation that the human $p53^{R248W}$ mutant represses the expression of CDH1 *in vitro* (Dusek and Attardi, 2011; Wang et al., 2009). However, within the mixed population of mutant and wild-type cells the tissue does not exhibit a barrier or fragility defect.

In non-UV-exposed epidermis, the early competitive dominance of the $p53^{*/wt}$ mutant is explicable in terms of cell-autonomous effects on progenitors. The short-term effects of low-dose UV light exposure may also be explained within this paradigm. UV irradiation increases the rate of cell division of both wild-type and mutant progenitors, but the imbalance of cell fate in mutant cells remains. The result is a substantial acceleration of epidermal colonization by $p53^{*/wt}$ cells.

DMBA/UV exposure leads to a complex and heterogeneous mutational landscape. After long-term UV exposure, the $p53^{*/wt}$ population declines but is not entirely displaced. We hypothesize that individual clone fates would then depend on the chance acquisition of additional mutations and the competitive fitness of neighboring mutant clones. It remains to be seen whether competitive interactions promote, inhibit, or are neutral in carcinogenesis. Properties that allow a particular mutant to survive in early clonal competition could differ from those required for outgrowth of neoplastic cell population (Aktipis et al., 2013). The stepwise acquisition of further mutations may then be key for breaking these evolutionary bottlenecks (Nowell, 1976).

This study argues that the patchwork of clones carrying oncogenic mutations in sun-exposed human epidermis is

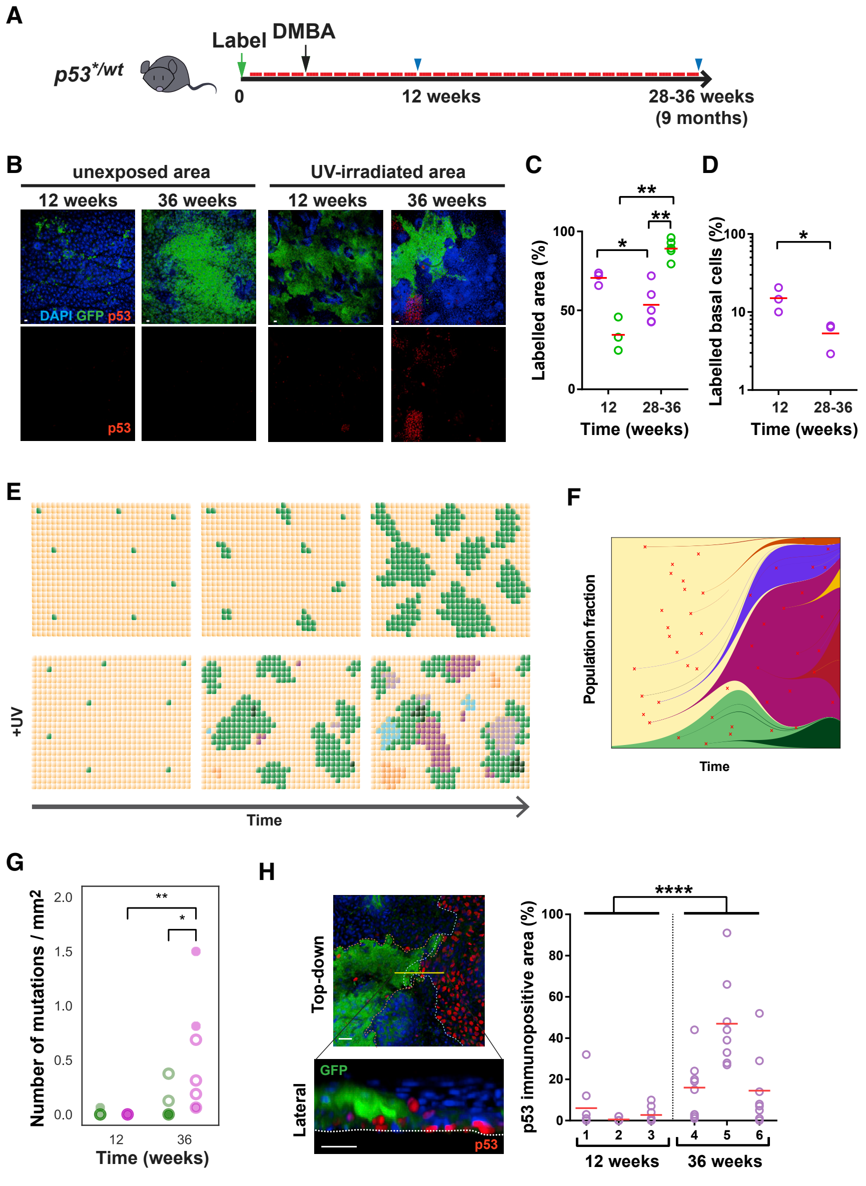

Figure 7. Long-Term UV Exposure Depletes Epidermis of *p53*$^{*/wt}$ Cells

(A) Protocol: *Ahcre*^{ERT}-*p53*$^{*/wt}$ mice were induced (green arrow), treated with a single dose of dimethylbenzanthracine (DMBA, black arrow), followed by repetitive sub-minimal erythema doses of UVB (red bars); blue arrows indicate sampling.

(B) Confocal z stacks showing projected views of UV-irradiated and an adjacent unexposed area of IFE. Blue, DAPI; green, GFP, reporting *p53** transcription; red, p53 indicating a p53 protein-stabilizing mutation. Scale bar, 20 μm.

(C) Labeled projected area of *p53*$^{*/wt}$ IFE in UV-exposed (purple circles; red line indicates mean value) and adjacent unexposed areas (green circles; red lines indicate mean value). n = 3 at the 12-week time point and n = 5 at the 28- to 36-week time point. Comparison between different time points, within same animal (unexposed versus UV irradiate area), **p = 0.0046, *p = 0.036 by unpaired two-tailed t test, **p = 0.0071 by paired t test.

(D) Proportion of labeled basal cells in UV-irradiated IFE at indicated time points. Values are mean percentage from 5–8 fields per animal. n = 3 mice per time point. Red bars, mean value. *p = 0.042 by unpaired two-tailed t test.

(E) Hypothesis: effect of prolonged low-dose UV on *p53*$^{*/wt}$ clonal dynamics. Following induction, in the absence of UV, *p53*$^{*/wt}$ clones (green) expand progressively in a background of wild-type cells (beige). In UV-exposed IFE, a wide variety of different mutant clones arises, indicated by multiple colors, some of which may expand, outcompete, and displace *p53*$^{*/wt}$ cells from the IFE.

(F) Simulation of clone competition under ongoing mutagenesis (see Video S1). A transgenic mutant (green) is induced at 1% frequency in a background of wild-type cells (yellow). Subsequently, new mutations (red cross, shades of green if in transgenic cells, other colors if in wild-type cells) are induced at random and assigned a fitness value as described in STAR Methods.

(G) The number of mutations detected per square millimeter by the Shearwater algorithm in each 16-mm^2 biopsy of mouse back skin: 3 induced and 2 non-induced mice at 12 weeks, 4 induced and 3 non-induced mice at 36 weeks. Solid circles, non-induced samples; open circles, induced samples. Green, non-exposed skin; purple, UV irradiated. **p < 0.01, *p = 0.02 by two-tailed Mann-Whitney test. Note that this method does not detect the induced and uninduced *p53** allele.

(H) Confocal z stacks showing *p53*$^{*/wt}$ clone in direct contact with p53-immunopositive clone in UV-irradiated area (36 weeks post-induction). Blue, DAPI; green, GFP; red, p53. Scale bar, 20 μm. Dashed lines indicate outline of clones in top-down image and basement membrane in lateral view. Graph on the right shows proportion of p53 immunopositive area in UV-irradiated skin. Measurement of 8 fields of view per mouse are plotted individually. n = 3 mice per time point. Red lines indicate mean values. ****p < 0.0001 by two-tailed Mann-Whitney test.

See also Figure S7 and Tables S6 and S7.

shaped by both phenotypic adaptation and cell competition. Understanding how mutant progenitor clones interact is key to understanding not only epidermal physiology, but also the formulation of rational approaches to prevent malignant transformation.

STAR★METHODS

Detailed methods are provided in the online version of this paper and include the following:

- KEY RESOURCES TABLE
- CONTACT FOR REAGENT AND RESOURCE SHARING
- EXPERIMENTAL MODEL AND SUBJECT DETAILS
  - Mice strains and induction of allele
  - Primary culture and *in vitro* induction of allele
- METHOD DETAILS
  - Ultraviolet (UV) irradiation
  - Wholemount sample preparation
  - Immunofluorescence
  - Imaging

- ○ Transmission Electron Microscopy
- ○ Clonal imaging
- ○ Epidermal permeability barrier function
- ○ Immunoblotting
- ○ Mass Spectrometry
- ○ Transcriptome analysis
- ○ Ultra-deep targeted sequencing
- STATISTICS
- QUANTITATIVE ANALYSIS AND MODELING
- DATA AVAILABILITY
- CODE AVAILABILITY

SUPPLEMENTAL INFORMATION

Supplemental Information includes seven figures, seven tables, and one video and can be found with this article online at https://doi.org/10.1016/j.stem.2018.08.017.

ACKNOWLEDGMENTS

This work was supported by a Medical Research Council Grant-in-Aid to the MRC Cancer unit and a core grant from the Wellcome Trust to the Wellcome Sanger Institute, 098051 and 206194. P.H.J. acknowledges support from a Cancer Research UK Programme Grant (C609/A17257). B.A.H. acknowledges support from the Royal Society (UF130039). We thank Esther Choolun and staff at the MRC ARES and Sanger RSF facilities for excellent technical support.

AUTHOR CONTRIBUTIONS

P.H.J., K.M., and G.S. designed experiments. G.P., M.H., V.K., and B.A.H. performed quantitative analysis and modeling. D.G. and K.M. performed electron microscopy. T.N. and S.H.O. performed bioinformatics analysis of RNA-seq data. A.C. and M.H. performed exome sequencing analysis. A.M.K. provided instruction in modeling and statistical inference.

DECLARATION OF INTERESTS

The authors declare no competing interests.

Received: February 2, 2018
Revised: May 10, 2018
Accepted: August 28, 2018
Published: September 27, 2018

SUPPORTING CITATIONS

The following references appear in the Supplemental Information: Gillespie (1976), (1977); Moran (1957); Tibor and Krapivsky, 2010.

REFERENCES

Abel, E.L., Angel, J.M., Kiguchi, K., and DiGiovanni, J. (2009). Multi-stage chemical carcinogenesis in mouse skin: Fundamentals and applications. Nat. Protoc. *4*, 1350–1362.

Aktipis, C.A., Boddy, A.M., Gatenby, R.A., Brown, J.S., and Maley, C.C. (2013). Life history trade-offs in cancer evolution. Nat. Rev. Cancer *13*, 883–892.

Alcolea, M.P., and Jones, P.H. (2014). Lineage analysis of epidermal stem cells. Cold Spring Harb. Perspect. Med. *4*, a015206.

Alcolea, M.P., Greulich, P., Wabik, A., Frede, J., Simons, B.D., and Jones, P.H. (2014). Differentiation imbalance in single oesophageal progenitor cells causes clonal immortalization and field change. Nat. Cell Biol. *16*, 615–622.

Alexandrov, L.B., Jones, P.H., Wedge, D.C., Sale, J.E., Campbell, P.J., Nik-Zainal, S., and Stratton, M.R. (2015). Clock-like mutational processes in human somatic cells. Nat. Genet. *47*, 1402–1407.

Allen, T.D., and Potten, C.S. (1974). Fine-structural identification and organization of the epidermal proliferative unit. J. Cell Sci. *15*, 291–319.

Anders, S., Pyl, P.T., and Huber, W. (2015). HTSeq–a Python framework to work with high-throughput sequencing data. Bioinformatics *31*, 166–169.

Benjamini, Y., and Hochberg, Y. (1995). Controlling the false discovery rate: A practical and powerful approach to multiple testing. J. R. Stat. Soc. B *57*, 289–300.

Berenblum, I., and Shubik, P. (1949). The persistence of latent tumour cells induced in the mouse's skin by a single application of 9:10-dimethyl-1:2-benzanthracene. Br. J. Cancer *3*, 384–386.

Clayton, E., Doupé, D.P., Klein, A.M., Winton, D.J., Simons, B.D., and Jones, P.H. (2007). A single type of progenitor cell maintains normal epidermis. Nature *446*, 185–189.

Darido, C., Georgy, S.R., and Jane, S.M. (2016). The role of barrier genes in epidermal malignancy. Oncogene *35*, 5705–5712.

Dobin, A., Davis, C.A., Schlesinger, F., Drenkow, J., Zaleski, C., Jha, S., Batut, P., Chaisson, M., and Gingeras, T.R. (2013). STAR: Ultrafast universal RNA-seq aligner. Bioinformatics *29*, 15–21.

Doupé, D.P., Klein, A.M., Simons, B.D., and Jones, P.H. (2010). The ordered architecture of murine ear epidermis is maintained by progenitor cells with random fate. Dev. Cell *18*, 317–323.

Doupé, D.P., Alcolea, M.P., Roshan, A., Zhang, G., Klein, A.M., Simons, B.D., and Jones, P.H. (2012). A single progenitor population switches behavior to maintain and repair esophageal epithelium. Science *337*, 1091–1093.

Dusek, R.L., and Attardi, L.D. (2011). Desmosomes: New perpetrators in tumour suppression. Nat. Rev. Cancer *11*, 317–323.

Frede, J., Greulich, P., Nagy, T., Simons, B.D., and Jones, P.H. (2016). A single dividing cell population with imbalanced fate drives oesophageal tumour growth. Nat. Cell Biol. *18*, 967–978.

Freije, A., Molinuevo, R., Ceballos, L., Cagigas, M., Alonso-Lecue, P., Rodriguez, R., Menendez, P., Aberdam, D., De Diego, E., and Gandarillas, A. (2014). Inactivation of p53 in human keratinocytes leads to squamous differentiation and shedding via replication stress and mitotic slippage. Cell Rep. *9*, 1349–1360.

Gerstung, M., Papaemmanuil, E., and Campbell, P.J. (2014). Subclonal variant calling with multiple samples and prior knowledge. Bioinformatics *30*, 1198–1204.

Gillespie, D.T. (1976). A general method for numerically simulating the stochastic evolution of coupled chemical reactions. J. Comput. Phys. *22*, 403–434.

Gillespie, D. (1977). Exact stochastic simulation of coupled chemical reactions. J. Phys. Chem. *81*, 2340–2361.

Jaiswal, S., Natarajan, P., Silver, A.J., Gibson, C.J., Bick, A.G., Shvartz, E., McConkey, M., Gupta, N., Gabriel, S., Ardissino, D., et al. (2017). Clonal hematopoiesis and risk of atherosclerotic cardiovascular disease. N. Engl. J. Med. *377*, 111–121.

Jonason, A.S., Kunala, S., Price, G.J., Restifo, R.J., Spinelli, H.M., Persing, J.A., Leffell, D.J., Tarone, R.E., and Brash, D.E. (1996). Frequent clones of p53-mutated keratinocytes in normal human skin. Proc. Natl. Acad. Sci. USA *93*, 14025–14029.

Kemp, R., Ireland, H., Clayton, E., Houghton, C., Howard, L., and Winton, D.J. (2004). Elimination of background recombination: Somatic induction of Cre by combined transcriptional regulation and hormone binding affinity. Nucleic Acids Res. *32*, e92.

Klein, A.M., Doupé, D.P., Jones, P.H., and Simons, B.D. (2007). Kinetics of cell division in epidermal maintenance. Phys. Rev. E Stat. Nonlin. Soft Matter Phys. *76*, 021910.

Klein, A.M., Brash, D.E., Jones, P.H., and Simons, B.D. (2010). Stochastic fate of p53-mutant epidermal progenitor cells is tilted toward proliferation by UV B during preneoplasia. Proc. Natl. Acad. Sci. USA *107*, 270–275.

Le, H.Q., Ghatak, S., Yeung, C.-Y.C., Tellkamp, F., Günschmann, C., Dieterich, C., Yeroslaviz, A., Habermann, B., Pombo, A., Niessen, C.M., and Wickström, S.A. (2016). Mechanical regulation of transcription controls Polycomb-mediated gene silencing during lineage commitment. Nat. Cell Biol. *18*, 864–875.

Li, H. (2013). Aligning sequence reads, clone sequences and assembly contigs with BWA-MEM. ArXiv, arXiv:1303.3997 https://arxiv.org/abs/1303.3997.

Lim, X., Tan, S.H., Koh, W.L., Chau, R.M., Yan, K.S., Kuo, C.J., van Amerongen, R., Klein, A.M., and Nusse, R. (2013). Interfollicular epidermal stem cells self-renew via autocrine Wnt signaling. Science *342*, 1226–1230.

Love, M.I., Huber, W., and Anders, S. (2014). Moderated estimation of fold change and dispersion for RNA-seq data with DESeq2. Genome Biol. *15*, 550.

Martincorena, I., Roshan, A., Gerstung, M., Ellis, P., Van Loo, P., McLaren, S., Wedge, D.C., Fullam, A., Alexandrov, L.B., Tubio, J.M., et al. (2015). Tumor evolution. High burden and pervasive positive selection of somatic mutations in normal human skin. Science *348*, 880–886.

McLaren, W., Gil, L., Hunt, S.E., Riat, H.S., Ritchie, G.R., Thormann, A., Flicek, P., and Cunningham, F. (2016). The Ensembl Variant Effect Predictor. Genome Biol. *17*, 122.

Miroshnikova, Y.A., Le, H.Q., Schneider, D., Thalheim, T., Rübsam, M., Bremicker, N., Polleux, J., Kamprad, N., Tarantola, M., Wang, I., et al. (2018). Adhesion forces and cortical tension couple cell proliferation and differentiation to drive epidermal stratification. Nat. Cell Biol. *20*, 69–80.

Moran, P.A.P. (1957). Random processes in genetics. Proc. Camb. Philos. Soc. *54*, 60–72.

Muller, P.A.J., and Vousden, K.H. (2014). Mutant p53 in cancer: New functions and therapeutic opportunities. Cancer Cell *25*, 304–317.

Nowell, P.C. (1976). The clonal evolution of tumor cell populations. Science *194*, 23–28.

Olive, K.P., Tuveson, D.A., Ruhe, Z.C., Yin, B., Willis, N.A., Bronson, R.T., Crowley, D., and Jacks, T. (2014). Mutant p53 gain of function in two mouse models of Li-Fraumeni syndrome. Cell *119*, 847–60.

Page, M.E., Lombard, P., Ng, F., Göttgens, B., and Jensen, K.B. (2013). The epidermis comprises autonomous compartments maintained by distinct stem cell populations. Cell Stem Cell *13*, 471–482.

Park, S., Gonzalez, D.G., Guirao, B., Boucher, J.D., Cockburn, K., Marsh, E.D., Mesa, K.R., Brown, S., Rompolas, P., Haberman, A.M., et al. (2017). Tissue-scale coordination of cellular behaviour promotes epidermal wound repair in live mice. Nat. Cell Biol. *19*, 155–163.

Rompolas, P., Mesa, K.R., Kawaguchi, K., Park, S., Gonzalez, D., Brown, S., Boucher, J., Klein, A.M., and Greco, V. (2016). Spatiotemporal coordination of stem cell commitment during epidermal homeostasis. Science *352*, 1471–1474.

Roshan, A., and Jones, P.H. (2012). Act your age: Tuning cell behavior to tissue requirements in interfollicular epidermis. Semin. Cell Dev. Biol. *23*, 884–889.

Roshan, A., Murai, K., Fowler, J., Simons, B.D., Nikolaidou-Neokosmidou, V., and Jones, P.H. (2016). Human keratinocytes have two interconvertible modes of proliferation. Nat. Cell Biol. *18*, 145–156.

Sabapathy, K. (2015). The contrived mutant p53 oncogene—beyond loss of functions. Front. Oncol. *5*, 276.

Sada, A., Jacob, F., Leung, E., Wang, S., White, B.S., Shalloway, D., and Tumbar, T. (2016). Defining the cellular lineage hierarchy in the interfollicular epidermis of adult skin. Nat. Cell Biol. *18*, 619–631.

Snippert, H.J., van der Flier, L.G., Sato, T., van Es, J.H., van den Born, M., Kroon-Veenboer, C., Barker, N., Klein, A.M., van Rheenen, J., Simons, B.D., and Clevers, H. (2010). Intestinal crypt homeostasis results from neutral competition between symmetrically dividing Lgr5 stem cells. Cell *143*, 134–144.

Song, H., Hollstein, M., and Xu, Y. (2007). p53 gain-of-function cancer mutants induce genetic instability by inactivating ATM. Nat. Cell Biol. *9*, 573–580.

Srinivas, S., Watanabe, T., Lin, C.S., William, C.M., Tanabe, Y., Jessell, T.M., and Costantini, F. (2001). Cre reporter strains produced by targeted insertion of EYFP and ECFP into the ROSA26 locus. BMC Dev. Biol. *1*, 4.

Tibor, A., and Krapivsky, P.L. (2010). Exact solution of a two-type branching process: Clone size distribution in cell division kinetics. J. Stat. Mech. *2010*, P07028.

Trichas, G., Begbie, J., and Srinivas, S. (2008). Use of the viral 2A peptide for bicistronic expression in transgenic mice. BMC Biol. *6*, 40.

Truong, A.B., Kretz, M., Ridky, T.W., Kimmel, R., and Khavari, P.A. (2006). p63 regulates proliferation and differentiation of developmentally mature keratinocytes. Genes Dev. *20*, 3185–3197.

Wang, S.P., Wang, W.L., Chang, Y.L., Wu, C.T., Chao, Y.C., Kao, S.H., Yuan, A., Lin, C.W., Yang, S.C., Chan, W.K., et al. (2009). p53 controls cancer cell invasion by inducing the MDM2-mediated degradation of Slug. Nat. Cell Biol. *11*, 694–704.

Yu, G., Wang, L.G., Han, Y., and He, Q.Y. (2012). clusterProfiler: An R package for comparing biological themes among gene clusters. OMICS *16*, 284–287.

Zhang, W., Remenyik, E., Zelterman, D., Brash, D.E., and Wikonkal, N.M. (2001). Escaping the stem cell compartment: Sustained UVB exposure allows p53-mutant keratinocytes to colonize adjacent epidermal proliferating units without incurring additional mutations. Proc. Natl. Acad. Sci. USA *98*, 13948–13953.

Zhu, J., Sammons, M.A., Donahue, G., Dou, Z., Vedadi, M., Getlik, M., Barsyte-Lovejoy, D., Al-awar, R., Katona, B.W., Shilatifard, A., et al. (2015). Gain-of-function p53 mutants co-opt chromatin pathways to drive cancer growth. Nature *525*, 206–211.

STAR★METHODS

KEY RESOURCES TABLE

REAGENT or RESOURCE	SOURCE	IDENTIFIER
Antibodies		
Caspase 3 (CAS3)	Abcam	Cat# ab44976; PRID: AB_868674
Caspase 3 (CAS3)	Abcam	Cat# ab2302; PRID: AB_302962
Green fluorescent	Life technologies	Cat# A10262; PRID: AB_2534023
Cyclo Butane prymidine	CosmoBio	Cat# NMDND001; PRID: AB_1962813
p53 (CM5)	Vector Laboratories	Cat# VP-P956; PRID: AB_2335917
p53 (Pab1801)	Abcam	Cat# ab26; PRID: AB_303198
Phospho Serine 392 p53	Millipore	Cat# 04-244; PRID: AB_1587353
Phospho Serine 15 p53	Cell signaling	Cat# 9284; PRID: AB_331464
Acetyl Lysine 379 p53	Cell signaling	Cat# 2570; RRID:AB_823591
Loricrin	Covance	Cat# PRB-145P; PRID: AB_292095
Filaggrin	Covance	Cat# PRB-417P; PRID: AB_291632
Cytokeratin 14	Covance	Cat# PRB-155P; PRID: AB_292096
Cytokeration 10	Abcam	Cat# ab 76318; PRID: AB_1523465
Tubulin β2	Abcam	Cat# ab151318
Mouse double minute 2	Abcam	Cat# ab16895; PRID: AB_2143534
CD45	Biolegends	Cat# 103102; RRID:AB_312967
E-cadherin (CDH1)	Cell signaling	Cat# 3195; PRID: AB_10694492
Desmoglein 3 (DSG3)	Santa Cruz Biotechnology	Cat# sc-23912; RRID:AB_627422
Beta Catenin (CTNNB1)	Cell signaling	Cat# 9562; PRID: AB_331149
Phalloidin	Life technologies	Cat# A22287; PRID: AB_2620155
Lrig1	R&D systems	Cat# AF3688; RRID:AB_2138836
Alexa Fluor 647 anti-human/mouse CD49f	BioLegend	Cat# 313610; PRID:AB_493637
Bacterial and Virus Strains		
Adeno-cre	Vector Laboratories	Cat# 1045
Chemicals, Peptides, and Recombinant Proteins		
β-napthoflavone	MP Biomedicals	Cat# 156738
Tamoxifen	Sigma	Cat# N3633
Doxycycline	Sigma	Cat# D9891
Fish Skin gelatin	Sigma Aldrich	Cat# G7765
Bovine Serum Albumin	Merk MIllipore	Cat# 126575
Donkey serum	Sigma Aldrich	Cat# D9633
Goat serum	Sigma Aldrich	Cat# G9023
Lucifer yellow	Sigma	Cat# L0259
Keratinocyte Serum Free media	Invitrogen	Cat# 10744018
Epidermal growth factor	GIBCO	Cat# 10450-013
Bovine pituitary extract	GIBCO	Cat# 13028-014
Polybrene	Sigma	Cat# H9268
0.25% trypsin	Sigma	Cat# T4424
HEPES	GIBCO	Cat# 15630080
HBSS	GIBCO	Cat#14175-053
Eagle's Minimum Essential Medium	Lonza	Cat# LZBE06-174G
SimplyBlue Coomassie G-250	Thermo Scientific	Cat# LC6060
Protease and Phosphatase inhibitor	Thermo Scientific	Cat# 78415
10% Tris-HCl acrylamide gel	Bio-Rad	Cat# 161-155
Fibronectin	BD	Cat# 356008

(*Continued on next page*)

Continued

REAGENT or RESOURCE	SOURCE	IDENTIFIER
Collagen Type I	BD	Cat# 354236
40, 6-diamidino-2-phenylindole (DAPI)	Sigma	Cat# D9542
Critical Commercial Assays		
Click-iTEdU imaging	Life technologies	Cat# C10086
Pierce TM Crosslink Magnetic IP/Co-IP kit	Thermo Scientific	Cat# 88805
RNeasy Mini kit	QIAGEN	Cat# 74106
Quantitect Reverse Transcription kit	QIAGEN	Cat# 205310
TaqMan Universal PCR Master Mix	Life technologies	Cat# 4304437
Lipofectamine 2000	Thermo Fisher	Cat# 11668-019
QuickSTART Bradford Dye reagents	BioRAD	Cat# 500-0202
Immobilon Western Chemiluminescent HRP substrate	Millipore	Cat# WBLUC0500
SuperSignal West Femto Chemiluminescent	Thermo Scientific	Cat# 34095
SimplyBlue Coomassie G-250 stain	Thermo Scientific	Cat# LC6060
PfuUltra II Fusion HS DNA polymerase	Agilent technologies	Cat# 600672
PCR purification kit	QIAGEN	Cat# 28106
Rapid DNA ligation kit	Roche	Cat# 11635379001
DNA MiniPrep kit	QIAGEN	Cat# 27106
Maxi Prep Endotoxin free kit	QIAGEN	Cat# 12362
Pierce TM Crosslink Magnetic immunoprecipitation (IP)/Co-IP kit	Thermo Scientific	Cat# 88805
QIAamp DNA Micro Kit	QIAGEN	Cat# 56304
Deposited Data		
RNaseq data: $p53^{wt/wt}$	This paper	ENA: ERS1755594, ERS1755602, ERS1755610, ERS1755618
RNaseq data: $p53^{*/wt}$	This paper	ENA: ERS1755595, ERS1755603, ERS1755611, ERS1755619
RNaseq data: $p53^{*/*}$	This paper	ENA: ERS1755596, ERS1755604, ERS1755612, ERS1755620
RNaseq data: $p53^{R245W/R245W}$ (untagged)	This paper	ENA: ERS1755597, ERS1755605, ERS1755613, ERS1755621
Ultra-deep targeted DNA sequencing data	This paper	ENA: ERP023080
Experimental Models: Cell Lines		
Mouse embryonic fibroblast	Laboratory of Ashok Venkitaraman	PMID:15607980
Human derived amphotrophic phoenix cell	ATCC	ATCC CRL-3213
Experimental Models: Organisms/Strains		
Mouse: C57BL/6J	The Jackson Laboratory	JAX: 000664
Mouse: $Trp53^{flR245W/flR245W}$	This paper	N/A
Mouse: $Ahcre^{ERT}$	Kemp et al., 2004	
Mouse: $Ahcre^{ERT}Trp53^{flR245W/wt}$	This paper	N/A
Mouse: $Ahcre^{ERT}R26^{flconfetti/wt}Trp53^{flR245W/wt}$	This paper	N/A
Mouse: $Ahcre^{ERT}Rosa26^{flYFP/wt}$	Clayton et al., 2007	N/A
Mouse: $R26^{M2rTA}$*TETO-GFP*	Doupé et al., 2012	N/A
Oligonucleotides		
Taqman assay *Mdm2*	Life technologies	Mm01233136_m1
Taqman assay *Tubb2b*	Life technologies	Mm01620966_s1
Recombinant DNA		
pBabe puro IRES-EGFP	addgene	Cat# 14430
pBabe puro IRES-EGFP p53	This paper	N/A

(*Continued on next page*)

Continued

REAGENT or RESOURCE	SOURCE	IDENTIFIER
pBabe puro IRES-EGFP p53^{R245W}	This paper	N/A
pBabe puro IRES-EGFP p53^{R245W}-T2A	This paper	N/A
pBabe puro IRES-EGFP p53^{S389A}	This paper	N/A
Software and Algorithms		
LAS X	Leica	N/A
Volocity 6 and 6.3	Perkin Elmer	N/A
AxioVision	Zeiss	N/A
Tecnai User Interface	FEI	N/A
GraphPad	Prism 6	N/A
STAR 2.5.3a	Dobin et al., 2013	N/A
HTSeq framework version 0.6.1p1	Anders et al., 2015	N/A
R package: DESeq2	Love et al., 2014	https://bioconductor.org/packages/release/bioc/html/DESeq2.html
R package: pheatmap	R Kolde, R package version 61'	https://cran.r-project.org/web/packages/pheatmap/index.html
R package: RColorBrewer	ColorBrewer palettes E Neuwirth, RC Brewer - R package version, 2014 - auckland.ac.nz	https://cran.r-project.org/web/packages/RColorBrewer/index.html
R package: clusterProfiler	Yu et al., 2012	https://bioconductor.org/packages/release/bioc/html/clusterProfiler.html
R package: org.Mm.eg.db	Carlson M (2018). org.Mm.eg.db: Genome wide annotation for Mouse. R package version 3.6.0	http://bioconductor.org/packages/release/data/annotation/html/org.Mm.eg.db.html
BWA-MEM (v0.7.15)	Li, 2013	
Shearwater	Gerstung et al., 2014	https://bioconductor.org/packages/release/bioc/html/deepSNV.html
Ensembl Variant Effect Predictor (Version 84)	McLaren et al., 2016	N/A
MATLAB R2016b	MathWorks	N/A
Jupyter & Spyder 3.1 (Python 3)	Python Software Foundation	N/A
Other		
Leica TCS SP5 II and SP8	Leica	N/A
120kV FEI Spirit Biotwin	FEI	N/A
StepOne Plus Real-Time PCR system	Life technologies	N/A
UV irradiater UV-2	Tyler research Corporation	N/A
UV-irradiator CL-508M	Uvitec	N/A

CONTACT FOR REAGENT AND RESOURCE SHARING

Request for reagent and resource sharing should be addressed to the Lead Contact, Philip H Jones (pj3@sanger.ac.uk).

EXPERIMENTAL MODEL AND SUBJECT DETAILS

Mice strains and induction of allele

All experiments were conducted according to UK government Home Office project licenses PPL22/2282 and PPL70/7543. Animals were maintained at specific and opportunistic pathogen free health status and were immune competent. No animals were involved in previous experiments and were drug naive prior to the start of experiments. Adult mice 12 weeks or more weeks in age were used for *in vivo* experiments. Animals were maintained on a *C57/Bl6* genetic background, housed in individually ventilated cages and fed on standard chow. Both male and female animals were used for experiments.

Trp53$^{flR245W\text{-}GFP/wt}$ knock-in mice were generated by TaconicArtemis GMBH, Germany. In the targeting vector, exons 5 to 11 of the wild-type *Trp53* gene were flanked by *loxP* sites and an additional transcriptional STOP cassette inserted between the *Trp53* 3′untranslated region (UTR) and the distal *loxP* site, in order to prevent transcriptional read through to downstream exons (Figure 2A). A second *Trp53* genomic region from exon 5 to exon 11, including the splice acceptor site of intron 4 was introduced 3′ of the distal

LoxP site. This duplicated region included a Trp53^{R245W} mutation introduced into exon 7 and a cassette including a glycine-serine-glycine flexible linker, a self-cleaving T2A peptide and the eGFP coding sequences inserted into exon 11, between the last codon of *Trp53* and the translation termination codon. Two positive selection markers were also introduced. A Neomycin resistance gene flanked by *Frt* sites was inserted 3′ of the 5′ *LoxP* site in exon 4, downstream of the predicted transcriptional initiation site. A Puromycin resistance gene flanked by F3 sites was placed downstream of the 3′UTR from the duplicated region. The targeting vector was generated using BAC clones from the *C57BL/6J RPCIB-731* BAC library and was fully sequenced. After transfection into the *C57BL/6N^{Tac}* embryonic stem cell line, clones that had undergone homologous recombination were selected using puromycin and neomycin and screened by Southern blotting.

The conditional *Trp53* strain was generated by *Flp*-mediated deletion of the selection markers. Prior to *cre*-mediated recombination these animals (*p53$^{*/wt}$*) express TRP53 protein from two wild-type alleles. Once the wild-type *Trp53* genomic region is deleted by *cre* both the *Trp53* mutant carrying the R245W mutation and the *eGFP* reporter are transcribed.

For lineage tracing of control, *Trp53* wild-type progenitors, the *Rosa26$^{flYFP/wt}$* (*R-YFP*) mice which express yellow fluorescent protein (YFP) from the constitutively active *Rosa 26* locus were used (Srinivas et al., 2001).

To assess the differentiation capacity of *p53$^{*/wt}$* mutant clones, homozygous *Ahcre^{ERT}R26fl^{Confetti}* animals were crossed onto *p53$^{*/*}$*to create *Ahcre^{ERT}R26fl$^{Confetti/wt}$p53$^{*/wt}$* animals (Snippert et al., 2010). Following induction this strain yields cells expressing one of 4 colors of reporter from the *Rosa26* locus and the GFP reporter of *p53* transcription. Cells doubly positive for red fluorescent protein and GFP were scored in experiments.

Each reporter line was crossed onto the *Ah^{creERT}* line in which transcription from a transgenic *CYP1A1* (arylhydrocarbon receptor, *Ah*) promoter is normally tightly repressed (Kemp et al., 2004). Following treatment with the non-genotoxic xenobiotic β-napthoflavone the *Ah* promoter is induced and a *cre* recombinase- mutant estrogen receptor fusion protein (creERT) is expressed. In the presence of tamoxifen, the cre^{ERT} protein enters the nucleus to mediate recombination.

For lineage tracing experiments, the relevant floxed reporter line was crossed onto the *Ah^{creERT}* strain. Doubly transgenic male and female animals were induced by a single interaperitoneal (i.p) injection of 80 mg/kg β-napthoflavone and 1 mg tamoxifen at 11-16 weeks of age. GFP and YFP expressing clones were visualized by immunostaining with an anti-GFP antibody.

To estimate the rate of epidermal cell division a transgenic proliferation assay was used (Doupé et al., 2012). Mice doubly transgenic for the reverse tetracycline-controlled transactivator (rtTA-M2) targeted to the *Rosa26* locus and a HIST1H2BJ-EGFP fusion protein (HGFP) expressed from a tetracycline promoter element were treated with doxycycline (DOX, 2 mg ml^{-1} in drinking water sweetened with sucrose) for 4 weeks. DOX was then withdrawn and animals were culled at different time points to track HGFP dilution.

To determine the proportion of proliferating cells, 100 μg of 5-ethynyl-2′-deoxyuridine (EdU) (Life technologies) was injected intraperitoneally 1 h before culling animals.

Primary culture and *in vitro* induction of allele

Primary mouse keratinocytes were isolated directly from tail epidermis of 4-12 week-old male and female *C57/BL6* wild-type, *p53$^{*/wt}$*, and *p53$^{*/*}$* mice and cultured in Keratinocyte Serum Free media (KSFM, Life technologies) supplemented with 1μg/ml Epidermal growth factor, 45 μg/ml Bovine pituitary extract, 20 mM HEPES-NaOH pH7.2-7.5, 1% Penicillin-Streptomycin and $CaCl_2$ to give a final Ca^{2+} concentration of 0.02 mM for growth media (GM) and 0.6 mM for differentiation media (DM).

Cre-mediated recombination was carried out *in vitro* using an adenovirus carrying *Cre* recombinase (Adeno-*cre*, Vector Laboratories cat.no. 1045). When primary cultures reached 50%–60% confluency, cells were incubated with Adeno-*cre* at 2x10^{6} pfu/ml in GM for 18 hours at 37°C. Virus was then removed by washing in Hank's Balanced Salt Solution (HBSS, Life technologies) three times. Cells maintained in GM for another 24 hours prior to use in experiments.

METHOD DETAILS

Ultraviolet (UV) irradiation

A UV irradiator UV-2 from Tyler Research Corporation was used for this study. During irradiation, animals were placed in a custom-made restrainer that restricted exposure to part of the dorsal skin. The dose of UVB-irradiation was titrated in wild-type C57BL/6 mice. A sub minimal erythema dose of UVB (750J/m^2) was determined from the appearance of the skin, Cyclobutane Pyrimidine staining and level of cleaved Caspase 3. *Ahcre^{ERT}-R-YFP* and *Ahcre^{ERT}-p53$^{*/wt}$* mice were lightly shaved on the back with electric shaver 3 days prior to the start of irradiation course, then exposed to sub MED UVB daily, 4 times a week, and shaved once a week. The irradiance was monitored by dosimeter every day. For short term treatment, mice were induced with β-napthoflavone and tamoxifen after 2 weeks of irradiation and irradiation continued as before. Animals were culled at intervals as described in the text.

UV-irradiation of cultured cells was performed with a UV irradiator (Uvitec, CL-508M with 5 × 8 W 312 nm tubes). Prior to UV-irradiation, cells were rinsed in HBSS briefly, exposed to a dose of 25 mJ/cm^2 UVB in HBSS and then placed in growth media.

Wholemount sample preparation

Whole back skin was lightly shaved and treated with hair removal cream (Nair Tough Hair, Coarse/Dark). The skin was then cut into rectangular pieces of approximately 4 by 5 mm and incubated in PBS containing 5mM EDTA at 37°C for 2 hours. Samples

were transferred into PBS and the epidermis was carefully scraped off using curved scalpel while holding one corner of the skin with forceps. The epidermal wholemounts were fixed in 4% paraformaldehyde in PBS for 30 minutes and then stored in PBS at 4°C.

Immunofluorescence

For staining, wholemounts were blocked in staining buffer (0.5% Bovine Serum Albumin, 0.25% Fish Skin Gelatin, and 0.5% Triton X-100 in PBS with 10% goat or donkey serum according to the secondary antibody used) for 1 hour at room temperature. Samples were incubated with primary antibody in staining buffer overnight, washed in PBS containing 0.2% Tween-20 four times, incubated with fluorchrome-conjugated secondary antibody for 2 hours at room temperature and washed as before After the final wash, samples were incubated with 40,6-diamidino-2-phenylindole (DAPI, 1 $\mu g\ ml^{-1}$) in PBS at least for 20 minutes and mounted on slides using Vectashield Mounting Medium with DAPI (Vector Labs).

EdU incorporation was detected with a Click-iT imaging kit (Life technologies) according to manufacturer's instructions.

Cryosections (20 μm thickness) were fixed in 4% paraformaldehyde in PBS for 10 minutes and stained as described above.

All immunofluorescence images are representative of at least 3 animals.

Imaging

Confocal images were acquired on Leica TCS SP5 II or SP8 microscopes using 10x, 20x or 40x objectives. Typical settings for acquisition of z stacks were optimal pinhole, line average 4 scan speed 400 Hz and a resolution of 1024 × 1024 pixels or 2048 × 2048 pixels. Image analysis was performed using Volocity 6 or 6.3 image processing software (Perkin Elmer).

Transmission Electron Microscopy

Small pieces of skin (1 mm^3) were fixed at 20°C for 2 hours in 2% paraformaldehyde with 2.5% glutalaldehyde in 0.1 M sodium cacodylate buffer at pH 7.42 with 0.1% $MgCl_2$ and 0.05% $CaCl_2$. They were then rinsed three times for 10 minutes each in sodium cacodylate buffer with chlorides and placed into 1% osmium tetroxide in sodium cacodylate buffer only, at room temperature for a further 2 hours, rinsed 3 times again, mordanted with 1% tannic acid for 30 minutes and rinsed with 1% sodium sulfate for 10 minutes. The samples were then dehydrated through an ethanol series and stained *en bloc* with 2% uranyl acetate for 1 hour at the 30% ethanol stage and embedded in Epon resin. Ultrathin transverse sections were cut on a Leica UC6 microtome and mounted onto grids before contrasting with uranyl acetate and lead citrate. Finally, images were recorded on an 120kV FEI Spirit Biotwin using an F4.15 Teitz CCD camera and measurements of the junction gaps made directly using Tecnai User Interface software.

Clonal imaging

After immunostaining wholemounts, clones were imaged by confocal microscopy and the number of basal and suprabasal cells in each clone counted in live acquisition mode.

Epidermal permeability barrier function

Epidermal barrier function was assessed by Lucifer yellow (λ_{ex} 428 nm, λ_{em} 540 nm) dye diffusion assay. Mouse back skin was lightly shaved and small pieces were placed dermis side down on PBS-soaked Whatman filter paper. 10 μL of 1 mM Lucifer Yellow (Sigma L0259) in PBS was applied onto the surface of the skin and parafilm was laid on it to ensure the sample was covered with the solution. Following incubation at room temperature for 1 hour, samples were frozen in Tissue-Tek O.C.T compound (Sakura) and cyrosections (20 μm) were analyzed by confocal microscopy.

Immunoblotting

Cells were lysed in buffer containing 20 mM HEPES NaOH pH7.2-7.5, Glycerol 10%, 0.4 M NaCl, NP-40 0.5% (Sigma), 0.2 mM EDTA, 1 mM Dithiothreitol (DTT), and 0.01% Halt Protease and Phosphatase inhibitor (ThermoFisher Scientific, cat.no.78415) and centrifuged at 13000 rpm at 4°C for 10 minutes. Protein concentrations were measured using standard Bradford protein assays (BioRAD QuickSTART Bradford Dye Reagents, cat.no.500-0202). Lysates were mixed with equal amount of 2x loading buffer (100mM Tris-HCl pH6.8, 4% SDS, 20% Glycerol, Bromophenol blue and 0.2% β-mercaptoethanol) and boiled at 96°C for 5 minutes. 4-10 μg of each sample was loaded onto a 10% or 12% of SDS-polyacrylamide gel. Proteins were separated by electrophoresis and transferred onto Immobilon-P membrane (pore size 0.45 μm, Millipore). Membranes were incubated in blocking buffer (5% dried skimmed milk, PBS, 0.1% Tween-20) at room temperature for 1 hour and then with primary antibodies diluted in blocking buffer for 1 hour at room temperature or overnight at 4°C on rocking platform. After washing in 0.1% Tween-20 PBS three times, HRP conjugated secondary antibodies (Dakocytomation) diluted in 0.5% skimmed milk in PBST were applied to the membrane for 30 min at room temperature on a rocking platform followed by three washes in 0.1% Tween-20 PBS 20 min each. Proteins were detected using Immobilon Western Chemiluminescent HRP substrate (Millipore WBLUC0500) or SuperSignal West Femto Chemiluminescent Substrate (Thermo Scientific, cat.no. 34095) for high sensitivity.

Mass Spectrometry

Sample preparation

cDNAs encoding wild-type murine *p53*, *p53*^{R245W} and *p53*^{R245W} with a C-terminal cleaved T2A mutant sequence were amplified by PCR using cDNA from transgenic animals. A *p53*^{S387A} mutant was constructed by PCR mutagenesis. To produce retroviruses, pBabe

puro IRES-EGFP plasmids carrying one of the above cDNAs were transfected into Amphotrophic phoenix producer cells (ATCC) using Lipofectamine 2000, according to manufacturer's instructions (ThermoFisher). Culture media containing retrovirus was treated with polybrene (final concentration 8 μg/ml) and used to infect primary *p53*$^{-/-}$ mouse embryonic fibroblasts (MEFs) (Olive et al., 2004). Whole cell lysate from the MEFs was prepared as described above, desalted using Amicon Ultra-0.5 Centrifugal Filter Unit with Ultracel-10 membrane and diluted with immunoprecipitation (IP) lysis/Wash buffer.

Immunoprecipitation

Approximately 4 mg of cell lysate was used for immunoprecipitation. CM5 p53 (Vector Labs Cat No #VP-P956) antibody was cross-linked with A/G magnetic beads using Pierce TM Crosslink Magnetic IP/Co-IP kit following the manufacturer's instructions (ThermoScientific Cat No #88805). The lysate was pre-cleared and applied to 150 μl of A/G magnetic beads cross-linked with the p53 antibody. The beads were then washed and the protein was eluted by boiling at 95°C for 5 min in non-reducing buffer (2 x concentration of 100 mM Tris-HCl pH 6.8 (Sigma SLBC0806V), 4% SDS, 20% Glycerol, Bromophenol blue (BDH prolabs 101184K). The total IP product was loaded on a 10% Tris-HCl acrylamide gel (Bio-Rad Cat No #161-155) and protein bands visualized with SimplyBlue Coomassie® G-250 stain following manufacturer's instructions (ThermoScientific Cat No #LC6060). The band at appropriate size was cut out and digested into peptides for liquid chromatography tandem-mass spectrometry.

Liquid Chromatography-Mass spectrometry/Mass spectrometry (LC-MS/MS)

Experiments were performed using a nanoAcquity UPLC (Waters Corp., Milford, MA) system and an LTQ Orbitrap Velos hybrid ion trap mass spectrometer (Thermo Scientific, Waltham, MA). Separation of peptides was performed by reverse-phase chromatography using a Waters reverse-phase nano column (BEH C18, 75 μm i.d. x 250 mm, 1.7 μm particle size) at flow rate of 300 nL/min. Peptides were initially loaded onto a pre-column (Waters UPLC Trap Symmetry C18, 180 μm i.d x 20mm, 5 μm particle size) from the nanoAcquity sample manager with 0.1% formic acid for 3 minutes at a flow rate of 10 μL/min. After this period, the column valve was switched to allow the elution of peptides from the pre-column onto the analytical column. Solvent A was water + 0.1% formic acid and solvent B was acetonitrile + 0.1% formic acid. The linear gradient employed was 3%–40% B in 60 minutes.

The LC eluent was sprayed into the mass spectrometer by means of a standard Thermo Scientific nanospray source. All *m/z* values of eluting ions were measured in the Orbitrap Velos mass analyzer, set at a resolution of 30000. Data dependent scans (Top 10) were employed to automatically isolate and generate fragment ions by collision-induced dissociation in the linear ion trap, resulting in the generation of MS/MS spectra. Ions with charge states of 2+ and above were selected for fragmentation. Post-run, the data was processed using Protein Discoverer (version 2.1., ThermoFisher). Briefly, all MS/MS data were converted to mgf files and the files were then submitted to the Mascot search algorithm (Matrix Science, London UK) and searched against a Uniprot *Mus musculus* database. A fixed modification of carbamidomethyl (C) and variable modifications of oxidation (M) and deamidation (NQ) were selected. A peptide tolerance of 10 ppm (MS) and 0.6 Da (MS/MS) were also selected along with 2 missed cleavages.

Transcriptome analysis

Total RNA extraction from cultured primary mouse keratinocytes was carried out using the QIAGEN RNeasy Mini kit according to manufactures instructions (QIAGEN). cDNA was synthesized using the Quantitect Reverse Transcription kit following manufactures instructions (QIAGEN) and diluted 20-foled prior to qPCR analysis.

qRT-PCR was performed on StepOne Plus Real-Time PCR system (Life technologies). Each reaction (20μl) contained 5μl cDNA, 10 μl of TaqMan® Universal PCR Master Mix (Life technologies) and 1 μl of an appropriate taqman probe. Following PCR parameters were used: hold 95°C for 10 min, followed by 45 cycles of step 1 at 94°C for 15 s, step 2 at 60°C for 55 s and acquiring to cycling A (FAM). All reactions were performed in triplicates. Data was analyzed using The StepOnePlus Real-Time PCR System and Excel.

Target gene	TaqMan probe ID (Life technologies)
Lce3d	Mm04337274_sH
Lor	Mm01962650_s1
Mdm2	Mm01233136_m1
Cdkn1a	Mm04205640_g1
Rprm	Mm00469773_s1
Fas	Mm01204974_m1
Dusp2	Mm00839675_g1
Tubb2b	Mm00849948_g1

For RNA-seq, libraries were prepared in an automated fashion using an Agilent Bravo robot with a KAPA Standard mRNA-Seq Kit (KAPA BIOSYSTEMS). In house adaptors were ligated to 100-300 bp fragments of dsDNA. All the samples were then subject to 10 PCR cycles using sanger_168 tag set of primers and paired-end sequencing was performed on Illumina's HiSeq 2500 with 75 bp

read length. Reads were mapped using STAR 2.5.3a, the alignment files were sorted and duplicate-marked using Biobambam2 2.0.54, and the read summarization was done using the script htseq-count from version 0.6.1p1 of the HTSeq framework (Anders et al., 2015; Dobin et al., 2013). Differential expression analysis was done using the DESeq2 R package(Love et al., 2014), and the downstream pathway analysis and visualization using R (https://www.R-project.org/) and the packages Pheatmap (https://cran.r-project.org/web/packages/pheatmap/index.html), RColorBrewer (https://cran.r-project.org/web/packages/RColorBrewer/index.html), clusterProfiler(Yu et al., 2012) and org.Mm.eg.db (http://bioconductor.org/packages/release/data/annotation/html/org.Mm.eg.db.html).

Ultra-deep targeted sequencing

Epidermal whole mounts, approximately 4 mm x 4 mm per piece, were prepared from UV-exposed and adjacent unexposed areas of the skin as described above. This protocol was run for both induced and non-induced $p53^{*/wt}$ animals. DNA was extracted using QIAGEN DNA micro kit (QIAGEN). A panel of 74 genes was chosen (see list below) based on the criteria of genes highly mutated in cutaneous squamous cell carcinomas and/or basal cell carcinomas, as well as genes frequently mutated in normal skin samples. The genes sequenced were:

Aff3, Ajuba, Arid1a, Arid2, Arid5b, Atm, Atp2a2, Bcl11b, Braf, Cacna1d, Card11, Casp8, Ccnd1, Cdkn2a, Cobll1, Crebbp, Ct*cf.*, Ctnnb1, Dclk1, Dclre1a, Dnmt3a, Ddr2, Egfr, Eif2d, Ep300, Erbb2, Erbb3, Erbb4, Ezh2, Fat1, Fat2, Fat3, Fat4, Fbxw7, Fbxo21, Fgfr3, Flt3, Grin2a, Hras, Kdm6a, Kdr, Kit, Kmt2c, Kmt2d, Kras, Lrp1b, Mtor, Nf1, Nf2, Notch1, Notch2, Notch3, Notch4, Nras, Pik3ca, Ptch1, Pten, Rb1, Ros1, Smad4, Smarca4, Smo, Sox2, Stat5b, Tert, Tet2, Tgfbr1, Tgfbr2, Trp53, Tsc1, Vhl, Zfp750, Nrf2, Keap1.

A custom bait capture panel (Agilent) was designed using Agilent SureDesign, targeting the exonic sequences of these 74 genes.

Paired-end 75bp read sequencing was performed on an Illumina HiSeq 2000_v4 machine. After removing reads for off-target capture and PCR duplicates, the average on-target coverage across samples was 1476x. The paired-end reads were aligned to the reference mouse genome (GRCm38) using BWA-MEM (v0.7.15)(Li, 2013). Variants were called using the latest version of Shearwater (unpublished). Shearwater is a variant caller designed to detect low frequency somatic variants that can be challenging to find using more conventional variant callers designed for germline variants (Gerstung et al., 2014). To avoid the probability of false positive variant calls, Shearwater builds a model of the background error rate for each base in the genome. This error model is most accurate when using matched normal samples from the same or closely related individuals to those for which variants are being called (to avoid calling common germline variants) and processed in the same way in the lab (to avoid shared artifacts introduced during sample processing and sequencing).

After running Shearwater, we detect 5888 putative variants summing across all skin biopsies. To reduce the number of false-positives we then applied a series of filtering steps. First, we removed any variants detected in both irradiated and unexposed skin biopsies from the same mouse. These are unlikely to be true somatic variants because we do not expect somatic clones to extend across two distant biopsies. To correct for multiple-hypothesis testing we then applied the Benjamini-Hochberg correction for the biopsies in each mouse independently, retaining only mutations with an adjusted q-value of less than 0.1(Benjamini and Hochberg, 1995). Finally, we removed variants without at least one supporting read from the forward and reverse strand. This resulted in a total of 67 filtered variants. The variants called here do not include the *p53** mutation, as this is removed by the Shearwater algorithm as a germline variant. The variants were annotated using Ensembl Variant Effect Predictor (Version 84)(McLaren et al., 2016)

STATISTICS

Source data and exact *P value*s for statistical tests are listed in the Table S7 for each Figure.

Statistical analysis was performed using the Graphpad Prism software. The D'Agostino-Pearson omnibus test was used to test for normality and the F-test to test for a significant difference in variance between groups. Student's unpaired t test was performed for normally distributed data where there was no significant difference in variance between groups. A two tailed paired t test was used where applicable. For non-normally distributed data, a two tailed Mann-Whitney test was performed.

No statistical method was used to predetermine sample size. The experiments were not randomized. The investigators were not blinded to allocation during experiments or outcome assessment.

QUANTITATIVE ANALYSIS AND MODELING

For details of quantitative analysis of wild-type (Figure S3A and S3B) and *p53* mutant progenitor cell lineage tracing data (Figures S6A and S6B), the dynamics of mutant cells in the suprabasal cell layers (Figures S6C and S6D) and a quantitative model of clonal competition during long-term ultraviolet light exposure (Figures 7F and S7E–S7G and Video S1) see STAR Methods, Quantitative Analysis.

DATA AVAILABILITY

Raw transcriptomic data can be viewed on https://www.ebi.ac.uk/ena using the following accession numbers: p53$^{wt/wt}$, ERS1755594, ERS1755602, ERS1755610, ERS1755618; p53$^{*/wt}$, ERS1755595, ERS1755603, ERS1755611, ERS1755619; p53$^{*/*}$, ERS1755596, ERS1755604, ERS1755612, ERS1755620; p53$^{R245W/R245W}$ (untag), ERS1755597, ERS1755605, ERS1755613, ERS1755621.

The accession number for the ultra-deep targeted DNA sequencing data reported in this paper is ENA: ERP023080.

CODE AVAILABILITY

Source code is accessible via Github:
https://github.com/PHJonesGroup/Murai_etal_SI_code

Cell Stem Cell, Volume *23*

Supplemental Information

Epidermal Tissue Adapts to Restrain Progenitors Carrying Clonal *p53* Mutations

Kasumi Murai, Greta Skrupskelyte, Gabriel Piedrafita, Michael Hall, Vasiliki Kostiou, Swee Hoe Ong, Tibor Nagy, Alex Cagan, David Goulding, Allon M. Klein, Benjamin A. Hall, and Philip H. Jones

Supplementary Materials for
Epidermal tissue adapts to restrain progenitors carrying clonal p53 mutations

Kasumi Murai, Greta Skrupskelyte, Gabriel Piedrafita, Michael Hall, Vasiliki Kostiou, Swee Hoe Ong, Tibor Nagy, Alex Cagan, David Goulding, Allon M Klein, Benjamin A Hall, Philip H Jones

This PDF file includes

Supplementary Figures and Legends

Figure S1. Related to Figures 3, 4 and 6

Figure S2. Related to Figure 3

Figure S3. Related to Figure 3

Figure S4. Related to Figures 2 and 3

Figure S5. Related to Figures 2 and 3

Figure S6. Related to Figures 3 and 4

Figure S7. Related to Figures 6 and 7

Supplementary Movie. Related to Figure 7

Supplementary Tables and captions, S1 to S7

Methods S1, Quantitative analysis, linked to STAR methods

Supplemental Figures and Legends

Figure S1

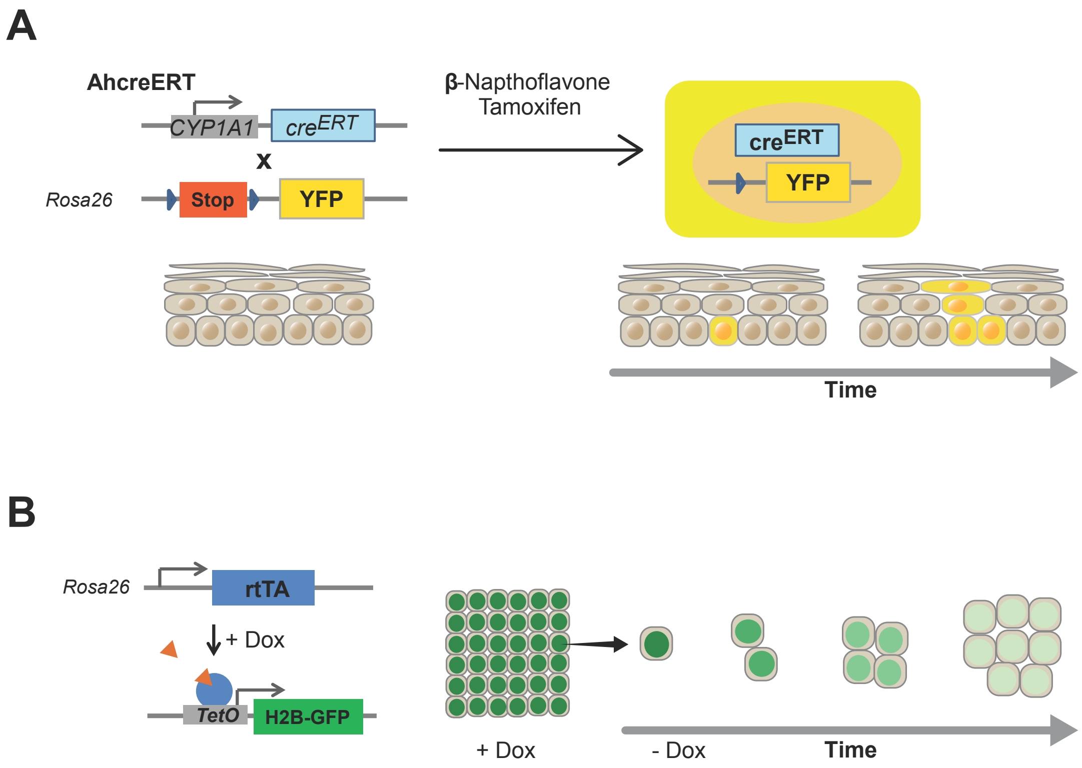

Figure S1. Related to Figures 3, 4 and 6; Transgenic assays used to track wild type cell behavior in this study

(A) Genetic lineage tracing in $Ahcre^{ERT}Rosa26^{flEYFP/wt}$ mice. *Cre*-mediated recombination, induced by β-napthoflavone (BNF) and tamoxifen (Tam), results in excision of stop cassette and the expression of the yellow fluorescent reporter (YFP). The reporter will be expressed in all the progeny of the recombined cell and form clusters of labelled cells termed clones.

(B) Quantification of the cell division rate. $R26^{M2rtTA}TETO\text{-}GFP$ animals carry a reverse tetracycline-controlled transactivator (rtTA-M2) and a HIST1H2BJ/EGFP fusion protein (HGFP) expressed from a tetracycline responsive promoter element. Doxycycline (dox) treatment in these animals results in nuclear labelling of all basal cells in the epidermis with HGFP. Upon withdrawal of dox, HGFP transcription ceases and the protein is diluted by cell division.

Figure S2

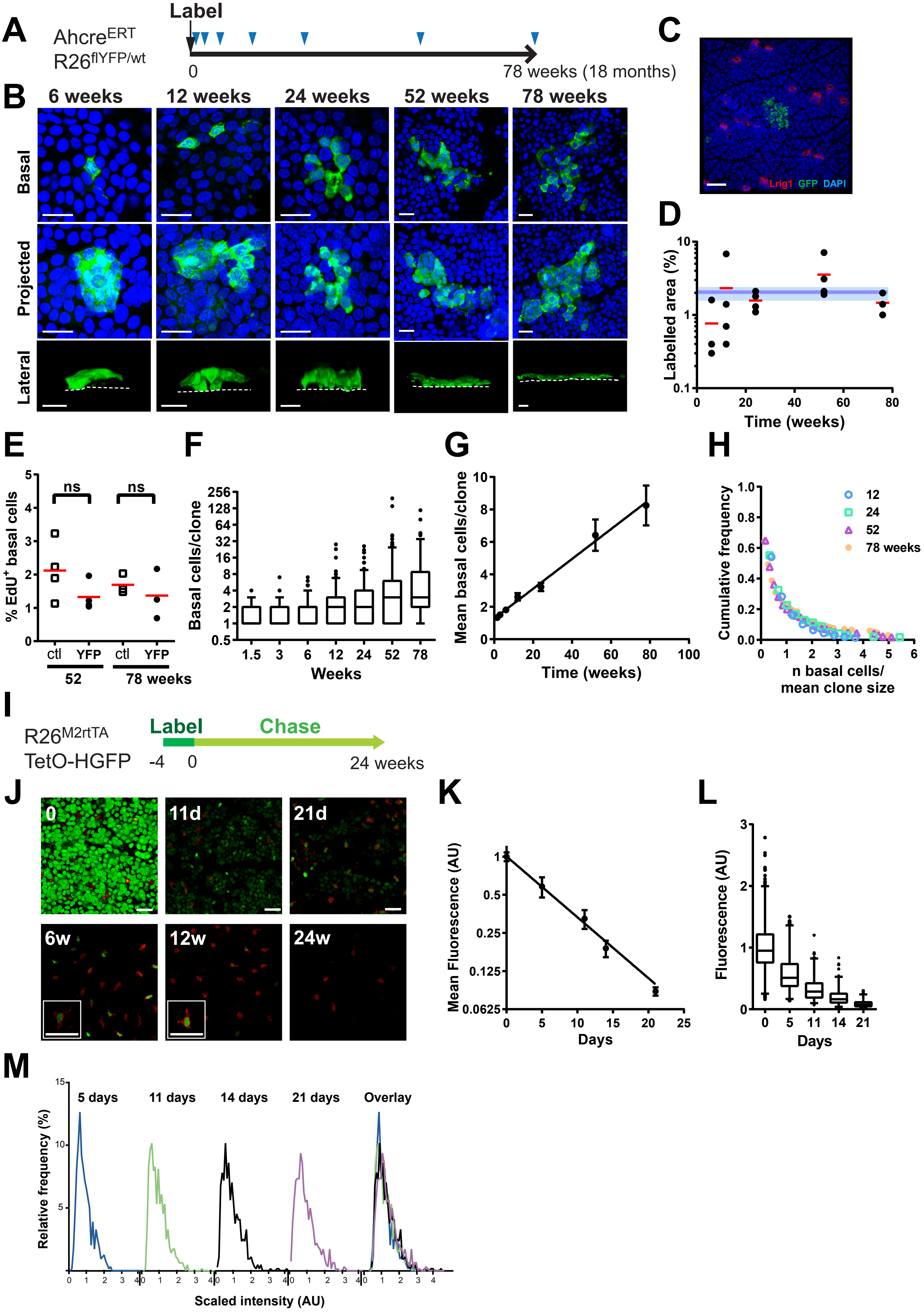
A
AhcreERT
R26flYFP/wt
Label
0
78 weeks (18 months)
B
6 weeks
12 weeks
24 weeks
52 weeks
78 weeks
Basal
Projected
Lateral
C
Lrig1 GFP DAPI
D
Labelled area (%)
Time (weeks)
E
% EdU+ basal cells
ns
ns
ctl
YFP
ctl
YFP
52
78 weeks
F
Basal cells/clone
Weeks
G
Mean basal cells/clone
Time (weeks)
H
Cumulative frequency
12
24
52
78 weeks
n basal cells/
mean clone size
I
R26M2rtTA
TetO-HGFP
Label
Chase
-4
0
24 weeks
J
0
11d
21d
6w
12w
24w
K
Mean Fluorescence (AU)
Days
L
Fluorescence (AU)
Days
M
5 days
11 days
14 days
21 days
Overlay
Relative frequency (%)
Scaled intensity (AU)

Figure S2. Related to Figure 3; Cell dynamics in wild-type mouse dorsal epidermis

(A) Lineage tracing protocol: Clonal frequency labelling was induced in the dorsal epidermis of $Ahcre^{ERT}$ $R26^{flEYFP/wt}$ mice and samples collected at intervals from 11 days to 18 months (triangles).

(B) Rendered confocal z stacks showing typical clones in epidermal wholemounts at times indicated. Basal, top-down view of basal layer; projected, top-down view through all nucleated cell layers; lateral, side view of clone. White line, basement membrane; Green, YFP; blue, DAPI; Scale bars, 20 µm.

(C) Confocal image showing a representative clone 68 weeks post induction in IFE of mouse back skin. Hair follicles are marked by high *Lrig1* expression (red). Green, YFP; blue DAPI. Scale bar, 100 µm. 16 clones were imaged and all confined to the IFE.

(D) Average % projected labelled area at indicated time points. Values are from 5 fields per mouse. Red lines indicate mean value at each time point. Blue line and shading show average and s.e.m. across all time points from 6 weeks to 72 weeks. n = 18 animals.

(E) Percentage of EdU labelled basal cells in YFP clones and in non labelled cells (ctl) in same mouse at 1 year (52 weeks) and 78 weeks. n = 4 mice at 52 weeks and n = 3 mice at 78 weeks. ns, no significant difference by paired t test.

(F) Clone size distribution (basal cells per clone, for ≥ 2-cell clones). Box plots show median (line across box), 25th and 75th percentiles (box) and 5th and 95th percentiles (whiskers), dots are outliers (dots), at indicated time points. Epidermal wholemounts from at least 3 mice per time point were analyzed. n = 93 clones at 1.5 weeks, 106 clones at 3 weeks, 181 clones at 6 weeks, 183 clones at 12 weeks, 209 clones at 24 weeks, 266 clones at 52 weeks and 140 clones at 78 weeks.

(G) Mean basal cells per clone at each time point is shown; increase is linear, $r^2 = 0.99$. Error bars indicate s.e.m of clones.

(H) Cumulative clone size distribution showing convergence onto scaling behavior. Cumulative size distribution, Cn(t), plotted as a function of number of basal cells per clone, n, normalized by the average clone size at each time point.

(I) Protocol: Adult $Rosa26^{M2rTA}$*/TetO-H2BGFP* mice treated with doxycycline (DOX) to express HGFP (Green). Following Dox withdrawal, HGFP transcription ceases and the protein is diluted upon cell division. Slow-cycling cells retain higher levels of HGFP protein. Samples were taken at 0, 5, 11, 21 day, 6, 12 and 24 week time points. Cells in

hair follicle opening regions were excluded from the analysis.

(J) Rendered confocal z stacks of mouse back skin epidermis showing HGFP (green) and immunostaining for the pan leukocyte marker CD45 (red) at indicated times. Scale bar, 30 µm. Insets: typical appearance of label retaining cells (LRC) at 6 & 12 weeks. All LRCs were CD45 positive (total 310 LRCs, n = 4 mice at 6 weeks).

(K) Dilution of HGFP in epidermis quantified by confocal imaging up to 21days time point . Values are mean fluorescence at time points indicated. Error bars are s.e.m of individual intensities. 160 nuclei / animal are quantified. n = 4 mice per time point.

(L) Quantification of HGFP dilution by confocal imaging. Box plots show median (central line), 25th and 75th percentiles (box) and 1st and 99th percentiles (whiskers) and outliers (dots), n = 640 nuclei from 4 mice at each time point. AU, arbitrary units.

(M) HGFP intensity distributions scale with time. Graphs show distributions of HGFP intensity divided by the average intensity of all values at that time point. The scaled distributions overlie each other (overlay), suggesting the cells belong to a single population in terms of HGFP dilution.

Figure S3

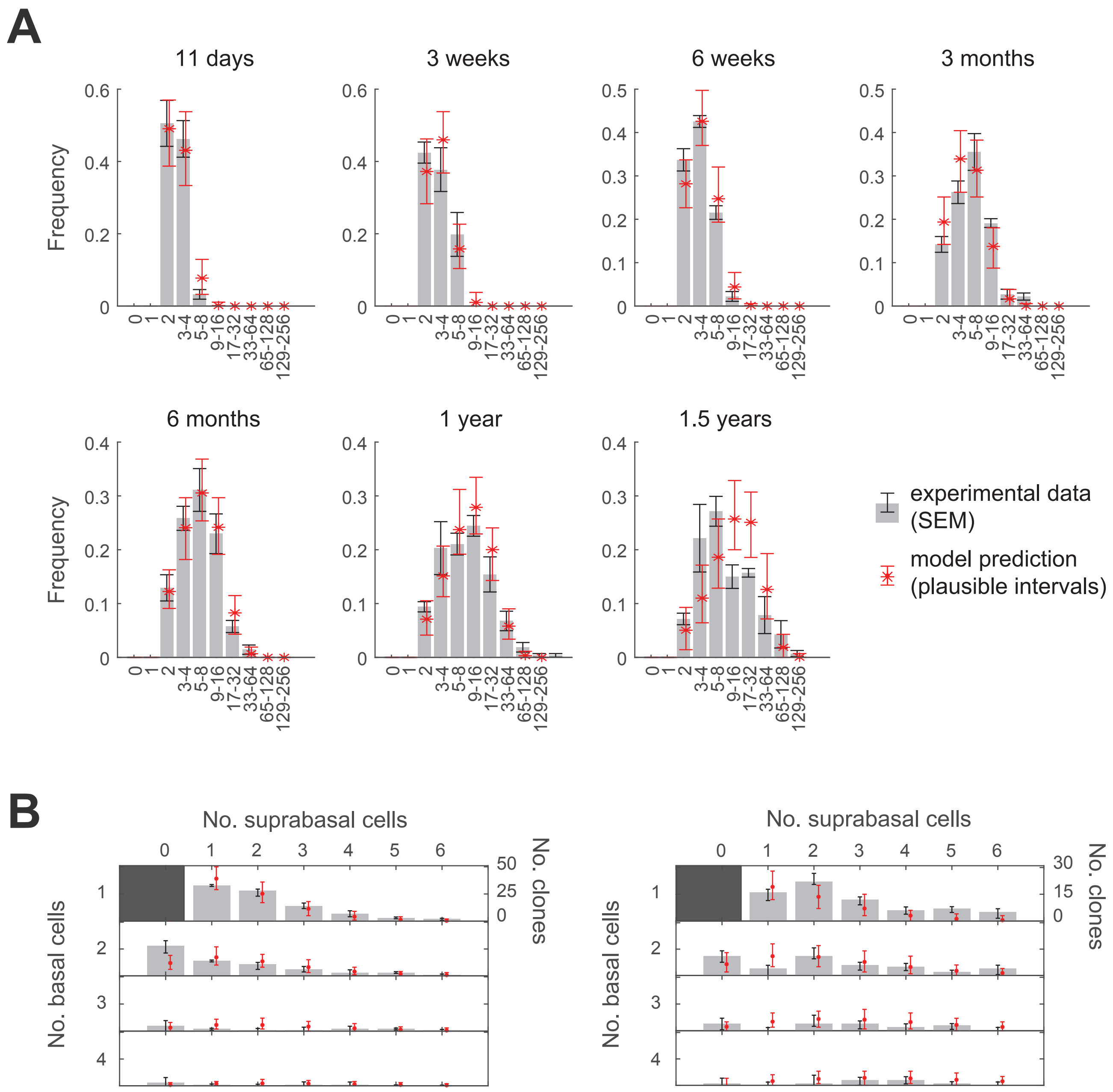
A
11 days
3 weeks
6 weeks
3 months
6 months
1 year
1.5 years
Frequency
0
1
2
3-4
5-8
9-16
17-32
33-64
65-128
129-256
experimental data (SEM)
model prediction (plausible intervals)
B
No. suprabasal cells
No. basal cells
No. clones

Figure S3. Related to Figure 3; *p53*$^{wt/wt}$ clone-size dynamics fit to single-progenitor model

(A) Total clone-size distributions obtained experimentally at different time points (grey bars show mean values; error bars refer to SEM; n = 3-5 mice per time point) are compared with the results of simulation of a single-progenitor model with balanced fates, with parameter values corresponding to those of the maximum likelihood estimate (red marks; see **Methods S1**) (the experimental suprabasal-to-basal cell ratio was further used to infer total sizes from basal estimates). Error bars in the model (red) represent *plausible intervals* defined as 95% likelihood intervals for each frequency value knowing the total number of clones observed at each time.
(B) Detailed clone sizes at early (6 weeks; left panel) and late (6 months; right panel) time points; frequencies displayed for *n* basal and *m* suprabasal clone sizes. Experimental data and model fit represented as in (A). In all cases floating clones (containing no basal cells) and single-cell clones were excluded from the analysis.

Figure S4

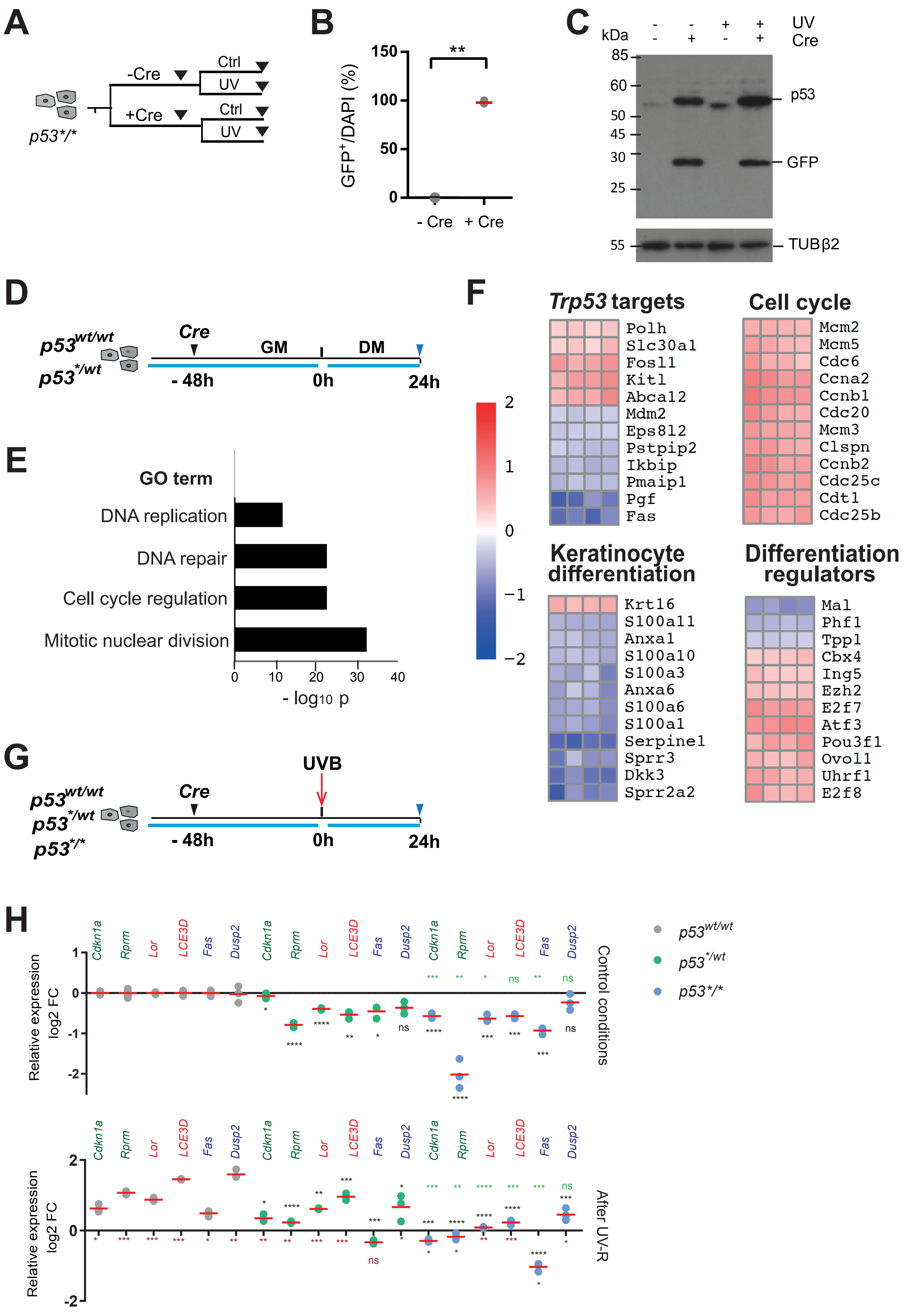
A
-Cre
+Cre
Ctrl
UV
Ctrl
UV
p53*/*
B
150
100
50
0
GFP+/DAPI (%)
**
- Cre
+ Cre
C
kDa
UV
Cre
85
60
50
45
30
25
55
p53
GFP
TUBβ2
D
p53wt/wt
p53*/wt
Cre
GM
DM
- 48h
0h
24h
E
GO term
DNA replication
DNA repair
Cell cycle regulation
Mitotic nuclear division
0
10
20
30
40
- log10 p
F
Trp53 targets
Polh
Slc30a1
Fosl1
Kitl
Abca12
Mdm2
Eps8l2
Pstpip2
Ikbip
Pmaip1
Pgf
Fas
Cell cycle
Mcm2
Mcm5
Cdc6
Ccna2
Ccnb1
Cdc20
Mcm3
Clspn
Ccnb2
Cdc25c
Cdt1
Cdc25b
Keratinocyte differentiation
Krt16
S100a11
Anxa1
S100a10
S100a3
Anxa6
S100a6
S100a1
Serpine1
Sprr3
Dkk3
Sprr2a2
Differentiation regulators
Mal
Phf1
Tpp1
Cbx4
Ing5
Ezh2
E2f7
Atf3
Pou3f1
Ovol1
Uhrf1
E2f8
2
1
0
−1
−2
G
UVB
p53wt/wt
p53*/wt
p53*/*
Cre
- 48h
0h
24h
H
Cdkn1a
Rprm
Lor
LCE3D
Fas
Dusp2
Relative expression log2 FC
Control conditions
After UV-R
p53wt/wt
p53*/wt
p53*/*

Figure S4. Related to Figure 2 and 3; Validation of conditional *p53* mutant transgenic mouse model

(A) Protocol for *in vitro* recombination: homozygous $p53^{*/*}$ mutant keratinocytes were either treated or not treated with Cre and either exposed or unexposed to UV-B to stabilize p53.

(B) Recombination efficiency in $p53^{*/*}$ keratinocytes assessed by GFP reporter. A graph shows GFP positive cells over DAPI. **p = 0.0022 by Mann Whitney test. n = 3 biological replicates.

(C) Expression of p53 R245W mutant protein following *cre*-mediated recombination *in vitro*. Immunoblot shows the expression of p53 mutant protein and GFP reporter at expected size respectively. Tubulin β2 was used as loading control. Note mutant protein is more stable than wild type p53 protein so can be detected without UV-irradiation.

(D) Protocol: Primary keratinocytes from $p53^{wt/wt}$ or $p53^{*/wt}$ mice were cultured in growth media (GM) and infected with adenovirus encoding *cre* recombinase (black arrow). Cells were then switched to differentiation media (DM) and lysed after 24 hours (blue arrow).

(E) GO enrichment analysis associated with biological processes in differentially-expressed genes (selected categories with $-\text{Log}_{10}$ p, adjusted for multiple testing, are shown).

(F) Heatmaps showing selected differentially expressed genes in $p53^{*/wt}$ and wild-type cultures. Color scale indicates $\log_2$ fold ratio, $p53^{*/wt}$ /$p53^{\text{wt/wt}}$, all genes shown have adjusted p value < 0.05. Full list of differentially expressed genes is in **Table S1**.

(G) Primary keratinocytes were isolated from $p53^{wt/wt}$, $p53^{*/wt}$ and $p53^{*/*}$mice. 48 hours after *in vitro cre*-mediated recombination, cells were exposed to 10 mJ/cm^2 UVB and transcriptional analysis was performed 24 hours later.

(H) Quantitative RT-PCR analysis for UV-inducible genes, *Cdk1na*, *Rprm* (cell cycle), *Lor*, *Lce3d* (terminal differentiation) and *Fas*, *Dusp2* (Apoptosis) with or without UVB irradiation (UV-R). Data are normalized housekeeping gene *Tubb2b* and presented as log2 fold change relative to $p53^{wt/wt}$ under control conditions. n = 3 independent experiments. Red horizontal line indicates the mean value. *p < 0.05; **p < 0.01; ***p < 0.001; ns, no significance; significance between genotypes was determined by two-tailed unpaired t-test, and between conditions (±UVB) by paired t-test. Black asterisks, comparison between $p53^{wt/wt}$ and mutant; Green asterisks, comparison between $p53^{*/wt}$

and *p53*$^{*/*}$mutants; Red asterisks, comparison between UV exposed and control conditions.

Figure S5

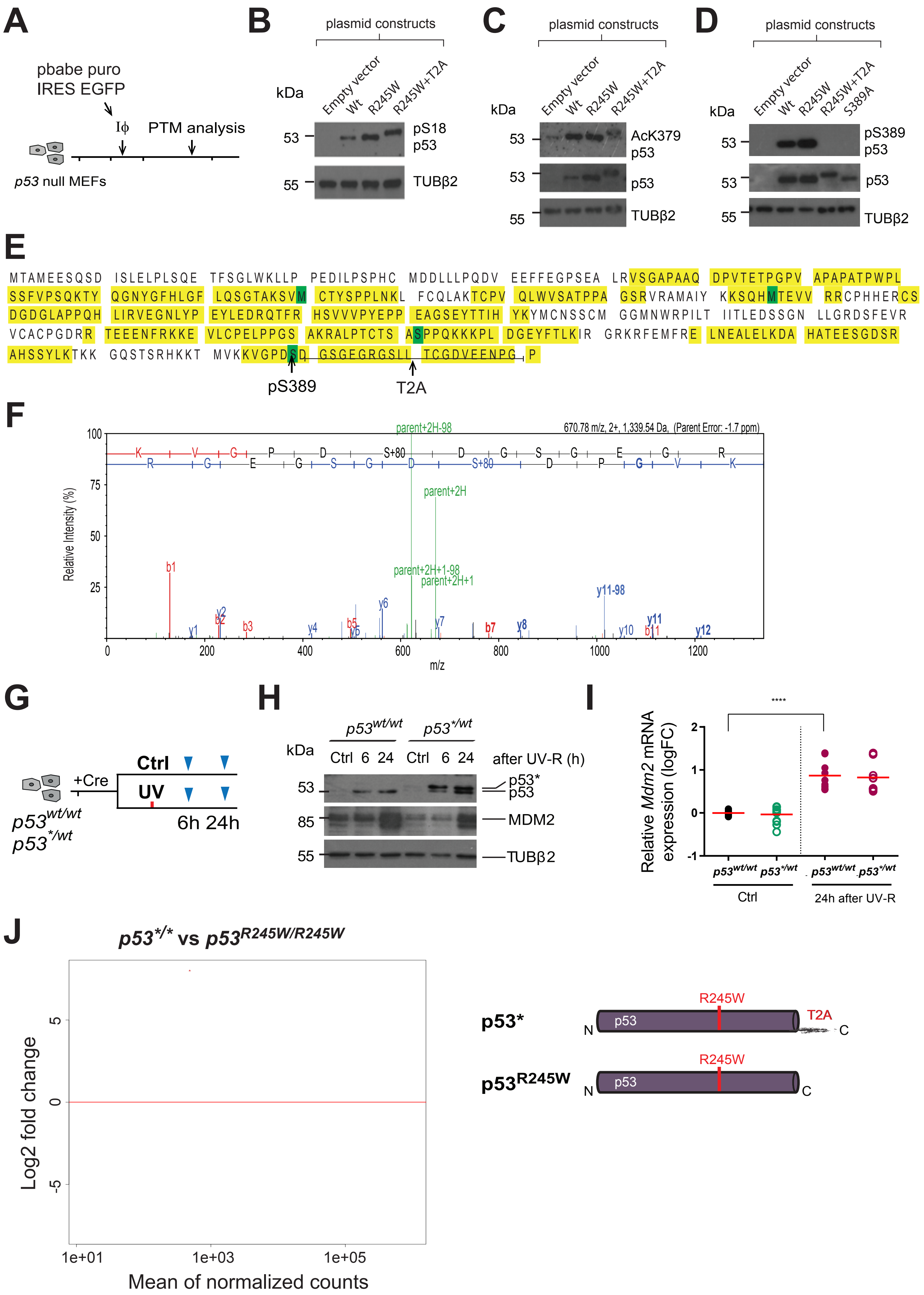

A
pbabe puro IRES EGFP
Iϕ
PTM analysis
p53 null MEFs
B
plasmid constructs
kDa
Empty vector
Wt
R245W
R245W+T2A
53
55
pS18 p53
TUBβ2
C
plasmid constructs
AcK379 p53
p53
TUBβ2
D
plasmid constructs
S389A
pS389 p53
p53
TUBβ2
E
MTAMEESQSD ISLELPLSQE TFSGLWKLLP PEDILPSPHC MDDLLLPQDV EEFFEGPSEA LRVSGAPAAQ DPVTETPGPV APAPATPWPL
SSFVPSQKTY QGNYGFHLGF LQSGTAKSVM CTYSPPLNKL FCQLAKTCPV QLWVSATPPA GSRVRAMAIY KKSQHMTEVV RRCPHHERCS
DGDGLAPPQH LIRVEGNLYP EYLEDRQTFR HSVVVPYEPP EAGSEYTTIH YKYMCNSSCM GGMNWRPILT IITLEDSSGN LLGRDSFEVR
VCACPGDRR TEEENFRKKE VLCPELPPGS AKRALPTCTS ASPPQKKKPL DGEYFTLKIR GRKRFEMFRE LNEALELKDA HATEESGDSR
AHSSYLKTKK GQSTSRHKKT MVKKVGPDSD GSGEGRGSLL TCGDVEENPG P
pS389
T2A
F
parent+2H-98
670.78 m/z, 2+, 1,339.54 Da, (Parent Error: -1.7 ppm)
parent+2H
parent+2H+1-98
parent+2H+1
Relative Intensity (%)
m/z
G
+Cre
Ctrl
UV
6h 24h
p53wt/wt
p53*/wt
H
kDa
Ctrl 6 24 Ctrl 6 24
after UV-R (h)
p53*
p53
MDM2
TUBβ2
I
Relative Mdm2 mRNA expression (logFC)
****
Ctrl
24h after UV-R
J
p53*/* vs p53R245W/R245W
Log2 fold change
Mean of normalized counts
p53*
p53R245W
R245W
T2A
N
C

Figure S5. Related to Figure 2 and 3; Characterization of p53* protein

(A-F) p53 post-translational modifications (PTM):

(A) Primary *p53* null mouse embryonic fibroblasts were infected with retroviruses carrying either wild type *p53* or one of the mutants $p53^{R245W}$, $p53^{R245W+T2A}$ or $p53^{S389A}$. PTM were analysed using total cell lysate .

(B-D) Immunoblots showing p53 phosphorylation at Ser18 (B) and Ser389 (C), and acetylation at Lys379 (D). Tubulin β2 was used as loading control. Anti–Ser389 antibody failed to detect phosphor-$p53^{R245W+T2A}$.

(E and F) Confirmation of phosphorylation of $p53^{R245W+T2A}$ at Ser389 by mass spectrometry analysis. (E) The analysis covered 62% of *Mus Musculus p53* variant 1 sequence (GenBank: AAB03324.1), which is highlighted in yellow. Green, phosphorylated amino acids. (F) The plot shows the relative current intensity produced by phospho-ions of varying mass-to-charge ratio (*m/z*).

(G) Protocol for *in vitro* protein stability study: $p53^{wt/wt}$ and $p53^{*/wt}$ mutant keratinocytes were *cre* recombined and exposed to UV-R. Cell lysate collected at 6 and 24 hours post irradiation.

(H) Immunoblot of p53 and its negative regulator, MDM2, before and after UV-irradiation in $p53^{wt/wt}$ and $p53^{*/wt}$ keratinocytes. A representative blot from three independent experiments is shown, Tubulin β2 is loading control.

(I) Quantitative RT-PCR showing the transcription of *Mdm2*. Data was normalised to *Tubb2b* expression and shown as $\log_2$ transformed value. n = 4 independent experiments, significance determined by Mann Whitney test, ****$P < 0.0001$.

(J) Confirmation of functional similarity between $p53^{*}$ and untagged $p53^{R245W}$. Primary keratinocyte from $p53^{*/*}$ and untagged $p53^{R24W/R245W}$ mice were subjected to *in vitro* cre-mediated recombination and kept in growth media for 48 hours. Cells were then switched into culture media permissive of differentiation and analysed by RNAseq 24 hours later. MA plot shows the differentially expressed genes between $p53^{*/*}$ and untagged $p53^{R24W/R245W}$ primary keratinocyte cultures. The black dots represent the genes with no significant difference. We detected only 22 significantly (DEseq2 padj < 0.05) differentially expressed genes shown in red and listed in **Table S1**, indicating retained T2A peptide has negligible effect on the $p53^{R245W}$ activity. Right: scheme showing tagged (p53*) and untagged ($p53^{R245W)}$ form of mutant proteins.

Figure S6

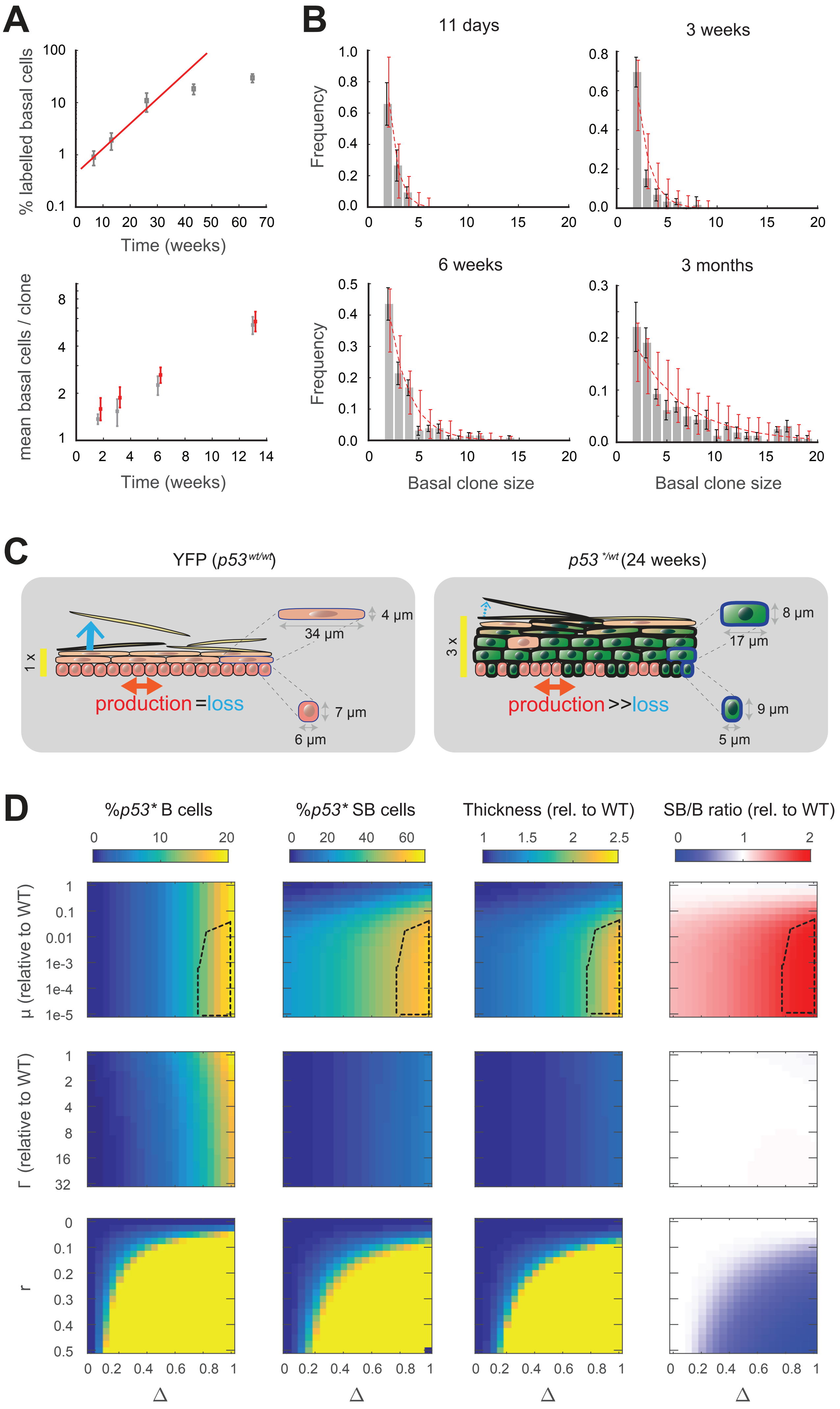

A
% labelled basal cells
Time (weeks)
mean basal cells / clone
Time (weeks)
B
11 days
3 weeks
6 weeks
3 months
Frequency
Basal clone size
C
YFP (p53 wt/wt)
p53 */wt (24 weeks)
1 x
3 x
4 µm
34 µm
7 µm
6 µm
8 µm
17 µm
9 µm
5 µm
production = loss
production >> loss
D
%p53* B cells
%p53* SB cells
Thickness (rel. to WT)
SB/B ratio (rel. to WT)
µ (relative to WT)
Γ (relative to WT)
r
Δ

Figure S6. Related to Figure 3 and 4; *p53*/wt* clonal dynamics fit with a single-progenitor model with fate imbalance

(A and B) Comparison of experimental and simulated results for the *p53*/wt* mutant population dynamics. Average percentage of labelled basal cells (A, top panel) and average basal clone sizes (A, bottom panel) are shown as well as the basal-layer clone size distributions over time (B). Experimental data are shown in grey (error bars indicate s.e.m.; n = 3-4 mice per time point). For the simulation, a single-progenitor model with unbalance fates was considered (see **Methods S1**). Error bars in the model (red) represent *plausible intervals* defined as 95% likelihood intervals for each frequency value knowing the total number of clones observed at each time. The population growth trend obtained through simulation is overlaid on **A**, top panel.

(C) Schematic: Changes in the epidermal structure between $p53^{wt/wt}$ (WT) and $p53^{*/wt}$ mice (6 months post-induction). Pink, $p53^{wt/wt}$ cells. Green, $p53^{*/wt}$ expressing cells. Typical values of cell sizes measured in each case in the basal and first suprabasal layers are displayed for comparison. These were used in cell-population dynamics simulations to infer epidermal thickness under different assumptions (see D). Both the pattern of $p53^{*/wt}$ cell colonization and the tissue thickening are consistent with a substantial reduction in shedding rate (blue arrows).

(D) Deterministic analysis of the $p53^{*/wt}$ mutant cell population dynamics and tissue properties under different parameter conditions (see **Methods S1**). Displayed are simulation results for the proportion of *p53** cells in the basal and suprabasal layers, the suprabasal-to-basal cell ratio, and tissue thickness at 24 weeks in $p53^{*/wt}$ induced epidermis. Measured cell sizes given in C were used for the latter. The degree of fate imbalance (Δ) was allowed to change along with shedding rate (μ), stratification rate (Γ), or division choice-related parameter (*r*). Only a substantial decrease in μ along with a Δ >> 0 could explain the observed accumulation of $p53^{*/wt}$ cells in the suprabasal layers and the increased thickness (dashed regions correspond with those parameter values yielding tissue properties that are quantitatively consistent with the observed data).

Figure S7

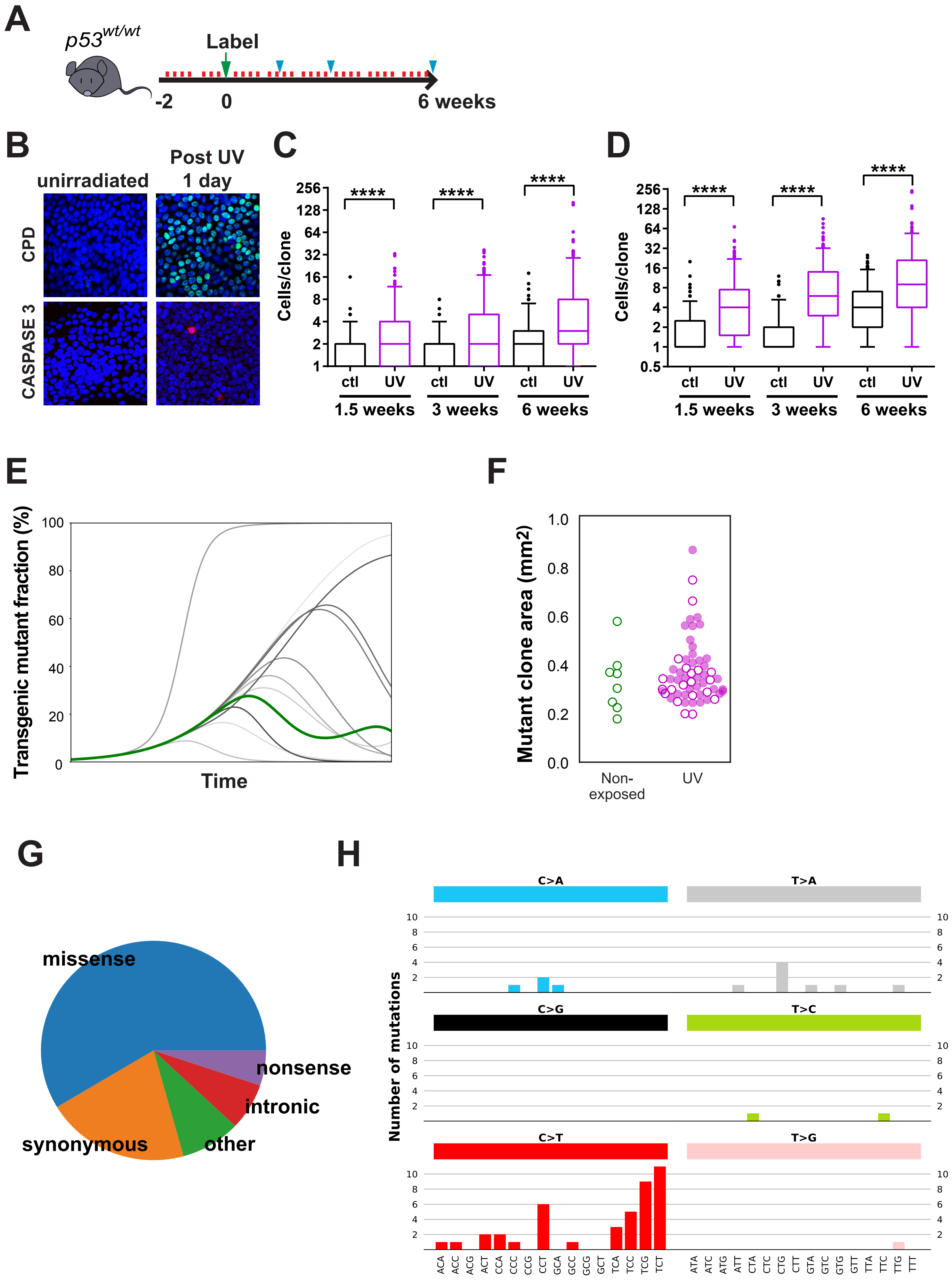

A
p53wt/wt
Label
-2
0
6 weeks
B
unirradiated
Post UV 1 day
CPD
CASPASE 3
C
Cells/clone
256
128
64
32
16
8
4
2
1
****
ctl
UV
1.5 weeks
3 weeks
6 weeks
D
Cells/clone
0.5
E
Transgenic mutant fraction (%)
100
80
60
40
20
0
Time
F
Mutant clone area (mm2)
1.0
0.8
0.6
0.4
0.2
0.0
Non-exposed
UV
G
missense
nonsense
intronic
other
synonymous
H
C>A
T>A
C>G
T>C
C>T
T>G
Number of mutations
10
8
6
4
2
ACA ACC ACG ACT CCA CCC CCG CCT GCA GCC GCG GCT TCA TCC TCG TCT
ATA ATC ATG ATT CTA CTC CTG CTT GTA GTC GTG GTT TTA TTC TTG TTT

Figure S7. Related to Figures 6 and 7; Titration of UVB dose and effect of UV exposure

(A) Protocol: mice were exposed to a sub-erythema dose of UVB daily, 4 days per week for 2 weeks (red lines) with shaving on the 5th day, labelling was induced and subsequently samples were collected (blue arrows).

(B) Confocal images showing basal layer of back skin epidermis. Animals were culled 24 hrs after 750 J/m^2 of UV-irradiation and wholemounts samples were prepared from UV-irradiated or unirradiated (ctl) back skin. Green, CPD; Red, Caspase-3; Blue, DAPI.

(C and D) Box plots showing basal (C) and total (D) clone size distribution in UV-irradiated epidermis (purple) and adjacent unexposed IFE (black) of induced *AhcreERT-RYFP* mice. Box plots show median (line across box), 25th and 75th percentiles (box) and 5th and 95th percentiles (whiskers), dots are outliers (dots), at indicated time points. UV, n = 220 clones at 1.5 weeks, n = 283 clones at 3 weeks, n = 229 clones at 6 weeks; control, n = 132 clones at 1.5 weeks, n = 131 clones at 3 weeks, n = 198 clones at 6 weeks. n = 4 mice per each time point. ****$p < 0.0001$ by by Mann Whitney test.

(E-G) Effect of long term UV exposure (see **Figure 7A** for the protocol) (E) Simulations of clone competition under ongoing mutagenesis (see **Supplementary Note 1.5** for details and **Supplementary Movie)**. A transgenic mutant is induced at 1% frequency in a background of wild-type cells. Subsequently, new mutations occur at random and are assigned a fitness value as described in Supplementary Note 1.5. The time course of the proportion of transgenic mutant cells in 10 independent simulations is shown. Green line indicates the outcome of the simulation captured in **Figure 7F**. (F) Estimated sizes of mutant clones detected in back skin biopsies of *p53*/wt* mice by the Shearwater algorithm (**Methods**). Clone sizes estimated by doubling the observed variant allele fraction (assuming the mutations were heterozygous). The induced *p53^{R245W}* mutation was not detectable by this method and is excluded. Solid circles, mutations from non-induced samples; open circles, mutations from induced samples. Green, non-exposed skin; Purple, UV irradiated. (G) Functional annotation of all mutations from the 36 week UV irradiated skin biopsies, according to Ensembl Variant Effect Predictor (Version 84). The "other" category includes "splice_region_variant", "splice_acceptor_variant" and "coding_sequence_variant".

(H) Trinucleotide plot of mutations in 36 week UV biopsies. The nucleotide change and immediate nucleotide context of all SNVs from the 36 week UV irradiated skin biopsies. It can be seen that the large majority of mutations are C>T, a feature of the UV mutation signature.

Supplementary Movie. Related to Figure 7; Simulation of clone competition under ongoing mutagenesis.

A visualization of the *non-spatial* simulation shown in **Figure 7F.** A transgenic mutant (green) is induced at 1% frequency in a background of wild-type cells (yellow). Subsequently, new mutations (shades of green if in transgenic cells, other colors if in wild-type cells) are induced at random and assigned a fitness value as described in **Methods S1**.

Supplementary Tables and captions

Table S1. Related to Figure 2, S4 and S5; Excel spreadsheet showing differentially expressed genes from RNAseq analysis

Table S2. Related to Figure 3 and 4; Proportion of Caspase 3 positive cells in back skin epidermis

	Animal	Total nuclei (fields)	Caspase 3⁺ cells	Proportion (%)
YFP (exp.1)	1	3007 (6)	15	0.499%
	2	2985 (6)	0	0.000%
p53/wt*	1	2586 (6)	3	0.116%
	2	3161 (6)	2	0.063%
YFP (exp.2)	1	112050 (27)	2	0.002%
	2	95450 (23)	3	0.003%

Table S3. Related to Figure 4; Proportion of EdU positive basal cells in *p53*/wt* areas at late time points

	Time point		
	6 months	10 months	15 months
animal 1	1.9	1.2	2.4
animal 2	1.9		0.7
animal 3	0.5		

% EdU⁺ basal cells per total GFP⁺ basal cells

Table S4. Related to Figure 4; Tumor-free survival

Genotype	Time after induction (months)			
	3	6	12	15
Induced *YFP*	0/17	0/12	0/7	0/3
Induced *p53*/wt*	0/23	0/20	0/15	0/4
Uninduced *p53*/wt*	n.d.	n.d.	0/3	n.d.

Values are numbers of epidermal tumors/total number of mice at each time point

Table S5. Related to Figure 6 and S7; Proportion of Caspase 3 positive cells in UV irradiated *p53*$^{wt/wt}$ skin.

	Basal	Suprabasal
control	0.0%	0.0%
1 day	0.7%	0.9%
1 week	0.4%	0.4%
2 weeks	1.1%	0.0%
4 weeks	1.6%	0.0%

Table S6. Related to Figure 7 and S7; Excel spreadsheet showing mutations detected by deep exome sequencing analyzed by Shearwater, see **methods** for details.

Table S7. Related to Figure 3-7, S2, S4, S5 and S7
Excel spreadsheet showing source data and statistical tests for figures.

Methods S1: Quantitative analysis of lineage tracing and clonal competition, related to STAR materials

Here we first discuss experiments used to track wild type epidermal progenitor cells, described in the main text in more detail (**Section 1.1**), along with quantitative analysis of the results (**Section 1.2**). **Section 1.3** covers the *p53* mutant progenitor cell dynamics, **Section 1.4** the dynamics of mutant cells in the suprabasal cell layers and **Section 1.5** considers whether clonal competition is a possible explanation for the loss of *p53* mutant clones during long-term ultraviolet light exposure.

1.1 Experiments to track wild type epidermal progenitor cell behavior

In this section, we first consider the genetic lineage tracing of dorsal IFE progenitor cells in $Ahcre^{ERT}$ $Rosa26^{flYFP/wt}$ mice introduced in the main text (**Figures S1A and S2A-S2H)**. In this experiment, a heritable genetic label (YFP) is induced at low frequency and is expressed in individual proliferating cells and their progeny. A key issue is whether the YFP-labelled cells are representative of all proliferating cells in the interfollicular epidermis (IFE). We noted that the proportion of labelled basal cells in IFE remained approximately constant over the duration of the experiment, indicating the YFP^+ population was self-sustaining and exhibited the cellular homeostasis characteristic of the entire IFE (**Figure S2D)**. Furthermore, there was no significant difference in the proportion of S-phase cells within YFP^+ clones and the surrounding non-labelled epidermis at 12 and 18 months after induction (**Figure S2E**). Taken together these results argue that labelled cells constitute a representative sample of proliferating epidermis. The characteristics of the evolving YFP^+ clone size distributions are discussed below in **section 1.2**.

A second experiment examined the rate of proliferation of cells in the epidermis by using the dilution of a transiently expressed transgenic histone–green fluorescent protein fusion (HGFP) to infer the mean rate of epithelial cell division (**Figures S1B and S2I-S2M)**(Doupe et al., 2012). Transgenic animals were treated with doxycycline (Dox) to induce HGFP transcription from a synthetic Dox-regulated promoter, resulting in high-level expression of HGFP throughout the epidermis (**Figures S1B, S2I and S2J**). Following withdrawal of Dox, HGFP levels were measured by confocal imaging of

epidermal whole mounts. Histone mRNA is short-lived, so HGFP transcript is unlikely to persist following Dox removal (Harris et al., 1991; Mascre et al., 2012). Assuming that the rate of HGFP protein degradation is small compared with the cell cycle time, the rate of HGFP decrease will reflect the rate at which it is diluted by cell division(Doupe et al., 2012). Slow cycling or quiescent cells will retain HGFP protein for long periods. We detected such HGFP retaining cells in IFE but all stained positive for the pan leukocyte marker CD45. No keratinocyte label-retaining cells were observed (**Figure S2J**). The mean level of HGFP in IFE keratinocytes fell with a half-life of 6 days and the form of the (unimodal) distribution scaled with time (**Figures S2K-S2M).** These results indicate that all cycling keratinocytes in dorsal skin IFE divide at a similar rate.

1.2. Wild type epidermal progenitor cell dynamics

Analysis of the evolving clone size distributions was used to test whether cell behavior in murine dorsal epidermis was consistent with the single-progenitor population model proposed in earlier work on squamous epithelial tissues (**Figure 1B**)(Clayton et al., 2007; Doupe et al., 2012). According to this model, the basal layer is populated by a single type of progenitor cell (A-cells) and differentiating cells (B-cells) which are post-mitotic. A-cells divide at a rate λ to give two daughter cells whose fates are determined stochastically: with a certain probability, the divisions are symmetric, yielding either two proliferating cells (A+A) or two differentiated cells (B+B), with the other divisions being asymmetric (A+B). Differentiating B cells leave the basal layer with a stratification rate Γ and become suprabasal cells (C-cells) that are ultimately lost by shedding at a rate μ.

$$A \xrightarrow{\lambda} \begin{cases} A + A & \text{Prob. } r \\ A + B & \text{Prob. } 1 - 2r \\ B + B & \text{Prob. } r \end{cases}$$

$$B \xrightarrow{\Gamma} C \qquad (1)$$

$$C \xrightarrow{\mu} \emptyset$$

In the simplest scenario, the probabilities that symmetric divisions lead to two proliferating cells or two differentiated cells are balanced, i.e. both are defined by a fixed parameter *r*, ensuring tissue homeostasis (**Figure 1B**). Under these conditions, one can assume that the proportion of proliferating cells in the basal layer, denoted as ρ, remains constant, and overall, the net rate at which post-mitotic cells are generated in the basal layer is compensated for by cell stratification and shedding (**Figure 1A**). It follows that

some relationships between the parameters can be established at homeostasis. In particular, $\Gamma = \lambda\rho/(1-\rho)$, and $\mu = \lambda\rho/m$, where the parameter m corresponds to the global ratio of suprabasal-to-basal cell populations.

Evidence from the YFP genetic lineage tracing experiments (**Figure S2**, **Section 1.1**) argues that the dynamics of the back-skin epidermis in *p53* wild-type ($p53^{wt/wt}$) mice conforms to the single-progenitor model with balanced fates(Klein et al., 2007). Firstly, despite the high variability of YFP labelling efficiency between individual mice, the percentage of area covered by labelled clones remains approximately constant over time, at around 2% (**Figure S2D**). Secondly, the average size of the persisting clones (i.e. those containing at least 1 basal cell) continuously expands with time, and the clone size distributions adopt a scaling behavior at late time points (**Figures S2F and S2H**).Thirdly, the average basal-layer clone size growth shows a linear increase over time (**Figure S2G**). The slope, sometimes denoted as τ^{-1} , can be identified as $r\lambda/\rho$ (Clayton et al., 2007). When analyzing back-skin clone sizes we find that $r\lambda/\rho$ = 0.08 ± 0.01 week^{-1} (95% confidence interval). We also find that the ratio of suprabasal cells to basal cells among labelled clones converges into a stationary value. Measurements of suprabasal-to-basal cells within relatively large persisting clones (containing at least 4 basal and 4 suprabasal cells) at late time points (≥ 6 months) allowed us to estimate m as 1.07 ± 0.17 (95% confidence interval). Finally, the pattern of HGFP dilution over a period of 5-21 days keeps very homogeneous, indicating a single type of progenitor cell (**Figures S2K-S2M**). The fluorescence intensity I decays exponentially with time, following $I_t = I_{t=0} * 2^{-\lambda t}$, the fitting giving an average division rate λ = 1.16 ± 0.06 week^{-1} (95% confidence interval)(Frede et al., 2016).

To demonstrate the validity of the model and fit clone size distributions with a single parameter solution we performed inference analysis on the unknown parameters based on the experimental basal-layer clone size distributions at different times. Using prior average estimates of λ, m and the parameter constraints given by the condition of homeostasis (see relationships above), the clonal dynamics can be estimated from the identification of the independent parameters r and ρ. For this, we implemented a maximum likelihood estimation (MLE) approach, iterating on different values of the

parameters r and ρ (grid search). A log-likelihood value $l(\theta; x)$ was obtained for every parameter set θ in the following way:

$$l(\theta; x) = \sum_t \sum_n (x_n(t) * \log p_n(t, \theta)) \quad (2)$$

where $x_n(t)$ is the observed frequency of clones with a basal size n at time t, and $p_n(t, \theta)$ is the probability of observing clones of that size at time t given the parameter values θ. Due to issues in computing the available analytical solution(Tibor and Krapivsky, 2010)in our analysis $p_n(t, \theta)$ was estimated for each parameter set from multiple simulations (100,000) of the Master equation, following Gillespie's algorithm (Gillespie, 1977; Gillespie, 1976). Given the particular scaling behavior of the clone size distributions and their large asymmetry, clone sizes were binned in ranges increasing in powers of two, as in previous work, i.e. n above stands for clones with a number of basal cells in the range 2^{n-1} to $(2^n - 1)$ (Clayton et al., 2007).

Only persisting clones with at least 2 basal cells were considered for the MLE analysis, to exclude any possible distortion due to post-mitotic cells labelled at induction. In addition, a small proportion (~1%) of late-time clones were reported with sizes that greatly exceeded the vast majority of their coexisting clones (i.e. sizes >> 2.3*SD above the mean clone size). These outlier clones were pooled in a common category (clones with >32 cells) to circumvent the computational issues of estimating the probability of extremely rare events with sufficient precision. However, excluding the outstandingly large clones from the analysis did not significantly alter the parameter estimates provided below.

The previous analysis gave the following maximum likelihood estimate on the parameters (95% confidence interval):

r = 0.06 (0.05; 0.09) ρ = 0.77 (0.69; 0.97)

This corresponds with the following values for the other (dependent) parameters: Γ = 3.9 (2.6; 38.0) week^{-1}, μ = 0.8 (0.6; 1.2) week^{-1}. Altogether, the MLE values $\hat{\theta}_{MLE}$ match well with the linear slope of the average clone size scaling, and the predicted clone size distributions fit well with the experimental total clone sizes over time, corroborating that the single progenitor model suits the cell dynamics in back skin epidermis (**Figures S3A and S3B**).

1.3. *p53*$^{*/wt}$ clonal expansion

In balanced, homeostatic conditions, the total labelled area remains constant, as seen in the data of the wild type, $p53^{wt/wt}$ (**Figure S2D**). However, the experimental data reveal a progressive increase in the area occupied by both $p53^{*/wt}$ basal and suprabasal cells (**Figures 3D and 4F**), which indicates that the mutant population is not in homeostasis.

In principle, one could try to explain this new scenario under the hypothesis of a single-progenitor model in balanced mode (1) in which some of the parameters have changed, leading to a transient period of growth before approaching a new stationary state.

Within the scenario of a balanced fate model, a reduction in stratification rate or an increase in division rate would lead to an increase in the basal population. This would proceed with an increase in the number of differentiated B cells n_B, as the A-cell population n_A remains constant, on average, in a balanced system. We would therefore expect to see a reduction in ρ as the proportion of B cells increases. This would mean a reduction in ρ to less than 0.03 given the increase in mutant population from <1% to 30% of the basal layer by 15 months. However, the fraction of EdU+ labelled basal cells is roughly constant and similar to wild type (**Table S3; Figure 3G**), and furthermore the proportions observed (~1.5%) appear incompatible with such a small value of ρ.

We would also expect to see a sublinear growth of the basal mutant population. Under the balanced model, new B cells are formed at a rate $\lambda^* n_A$ and stratify at a rate $\Gamma^* n_B$:

$$\frac{dn_B}{dt} = \lambda n_A - \Gamma n_B \qquad (3)$$

As $p53^{*/wt}$ suprabasal cells are observed (**Figure 4B and 4D**), we know that Γ > 0. Since the production rate is constant (fixed n_A), the growth rate dn_B/dt will decrease following an increase in the B cell population n_B, leading to a sublinear growth. Eventually the population would approach a new homeostatic state where production equals loss. In contrast to the expected balanced behavior, up to 6 months the mutant subpopulation can be seen to grow superlinearly from an initial small population, to ~10 % of the basal compartment (**Figure 3D**).

The previous features lead us to exclude the balanced mode as a model for the mutant dynamics. It follows, as we show below, that the data is consistent with a model including a cell fate imbalance:

$$A \xrightarrow{\lambda} \begin{cases} A+A & \text{Prob. } r(1+\Delta) \\ A+B & \text{Prob. } 1-2r \\ B+B & \text{Prob. } r(1-\Delta) \end{cases} \quad (4)$$

This model has been used previously to describe the clonal expansion of mutant epithelial progenitors(Klein et al., 2010) and can be described (in global, deterministic terms) by the following set of ODEs, whose solutions adopt an exponential shape:

$$\frac{dn_A}{dt} = 2\Delta r \lambda n_A$$
$$\frac{dn_B}{dt} = \lambda n_A - 2\Delta r \lambda n_A - \Gamma n_B \quad (5)$$
$$\frac{dn_C}{dt} = \Gamma n_B - \mu n_C$$

In this imbalanced case, there is an additional parameter and less available data to use for fitting than for the wild type fitting (note that *p53*$^{*/wt}$ clones converge and become fused after 3 months post-induction). We will therefore aim only to provide some simple explanations for the global dynamics and to show that there exist at least some parameter values for which the unbalanced model is consistent with the early stages of the *p53*$^{*/wt}$ mutant growth.

For the sake of simplicity, we have taken the parameter values from the wild type MLE $\hat{\theta}_{MLE}$ and we have made a minimum adjustment with the addition of a positive Δ. The small number of *p53*$^{*/wt}$ floating clones at the 12 week time point suggests that Δ cannot be equal to one. Based on these assumptions, we ran multiple stochastic simulations using Gillespie's Monte-Carlo algorithm with the following set of parameters:

$\lambda = 1.16$ week^{-1} $\quad r = 0.06 \quad \Gamma = 3.9$ week^{-1} $\quad \Delta = 0.95$

The results are shown in **Figures S6A and S6B**, where the simulation outcome is overlaid on the experimental data with reasonable fits, both in terms of average mutant cell populations as well as clone size distributions. In addition, one can retrieve the steady-state value of ρ from the solutions of the ODEs (5). This value ($\rho^{ss} = 0.80$) is similar to the wild type estimate (ρ = 0.77), in agreement with the similarity in the observed EdU data (**Table S4; Figure 3G**). While we do not claim that these are the best-fitting parameters, the consistency of the simulated results with the observed data suggests that the imbalanced fate model (4) is a plausible model to describe the first 6 months of *p53*$^{*/wt}$ growth.

In the case under UV irradiation, there is a large increase in the rate of $p53^{*/wt}$ colonization (**Figures 6B and 6F**). This could be explained as a result of rise in the cellular turnover (**Figures 6C and 6D**). Notice that an increase in λ would accelerate the expansion of $p53^{*/wt}$ clones according to the equations (5).

1.4 Dynamics of mutant suprabasal cells

The percentage of projected area labelled also showed a supralinear increase over the first 24 weeks post-induction (**Figure 4F**). However, this represented up to 70-80% coverage after 24 weeks in contrast with the value of 10-15% GFP+ cells reached in the basal layer. Although the projected area may partially depend on the degree of vertical stacking and lateral movement of the clonal cells, this apparent mismatch suggests an over-accumulation of the $p53^{*/wt}$ cells in the suprabasal layers. This is further supported by the increase in the epidermal thickness (**Figures 4I and 4J**) and the doubling of the keratinocyte density in the first suprabasal layer by 24 weeks (**Figures 4G and 4H**), compared with the modest increase in basal cell density (around 10%, **Figure 3F**).

To evaluate some simple possible explanations that could lead to this scenario we proceeded to numerically solve the set of ODEs that describe the unbalanced-system dynamics (5) under different hypotheses in which not only Δ but the value of other parameters of the model is allowed to vary from its configuration for the WT ($p53^{wt/wt}$). Only λ was left unaltered consistent with EdU data (**Figure 3G**). Setting an initial condition of 1% $p53^{*/wt}$ proliferating cells (in the range of the labelling induction efficiency), we tracked for each case the simulated time courses in the proportion of mutant basal population, the proportion of mutant suprabasal population, as well as the derived changes in ρ, *m*, and thickness, and compared these to the experimental observations at 6 months (**Figure S6D**). Note that simulated values for tissue thickness were estimated by converting the non-spatial cell populations into volumes using average basal and suprabasal cell sizes measured from rendered confocal z stacks – **Figures 4J and S6C**).

We observe that a large value for Δ, i.e. highly unbalanced stochastic division fates, was sufficient to explain the given changes in the basal compartment, but could only, by itself, explain a modest increase in the proportion of $p53^{*/wt}$ cells in the suprabasal layer (up to

20-30% by 6 months at most, with little change in tissue thickness). Similarly, the suprabasal fraction would not be significantly altered if there was also an increase in the rate of stratification (value of Γ) and thickness would be largerly unaffected. In contrast, small changes in r could dramatically alter the proportions of *p53*$^{*/wt}$ cells both in the basal and the suprabasal layers during the 6-months period, but the suprabasal-to-basal cell ratio indicates that mutant expansion would proceed in that case with a bigger relative cell accumulation in the basal compartment; hence, hard to reconcile with the measurements reporting just small changes in basal cell density (**Figure 3F**). We find that a large reduction in the shedding rate μ is the simplest plausible scenario. For instance, when setting values for μ 1-2 orders of magnitude smaller than in WT in conjunction with a large value of Δ (keeping other parameters as in WT), predictions are compatible with the experimental data not only in terms of the magnitude of growth in the proportion of *p53*$^{*/wt}$ cells in the basal layer, but also in terms of the mutant over-crowdedness in the suprabasal compartment. Furthermore, the moderate increase in thickness is also explained (**Figure S6D**).

1.5 Clonal competition during ongoing mutagenesis

Here we consider the behavior of *p53*$^{*/wt}$ cells induced at low frequency in mouse epidermis treated with the mutagen dimethylbenzanthracene and exposed to low levels of UV irradiation over a prolonged period. In the experiment, the area of IFE occupied *p53*$^{*/wt}$ cells increased before decreasing (**Figures 7C and 7D**). Detailed resolution of the multiple cellular and molecular mechanisms that may underpin the growth and decline of *p53*$^{*/wt}$ cells in this experiment is beyond the scope of this paper. However, we hypothesized that this behavior could be a consequence of competition between *p53*$^{*/wt}$ cells and cells carrying newly acquired mutations.

UV irradiation may represent a new selective pressure and cause an ongoing process of mutagenesis. New mutations occur at random throughout the UV irradiated epidermis (**Figure 7G**). Some of these can convey a competitive advantage over wild type cells and form expanding clones. At early times after induction, the *p53*$^{*/wt}$ cells are surrounded by wild type cells over which they have a proliferative advantage and the *p53*$^{*/wt}$ area grows, but the majority of the new mutations will occur within the wild type cells, since they occupy most of the tissue. Once expanding clones come into contact with each other, the

clones may compete, and the fitter clone may displace the weaker (**Figure 7E**). Most clones with a competitive advantage over wild type cells would eventually come into contact with a stronger clone, in which case we may expect to see a pattern of initial growth followed by decline, as shown in the experiments. Note that different factors may influence the competitive advantage of a clone at this stage, which may include its proliferation rate but also resilience to mechanical stress (cell crowdedness), etc.

To test whether the induction of new mutations could provide an explanation for the growth and decline of a specific cell population we ran simulations of clone growth under ongoing mutagenesis under very simple assumptions. The simulations track the relative populations of clones under two basic rules:

1. Clones grow or shrink depending on their fitness relative to the other clones in the simulation. The population of the weaker clones will decrease while stronger clones will expand.
2. New clones are generated at a certain rate by randomly occurring mutations and assigned a random fitness value.

We start the simulations with a large number of wild type cells and a small population of cells carrying a transgenic mutation, which has a slightly higher fitness than the wild type and is analogous to the $p53^{*/wt}$ in the experiment.

In a first implementation, populations were simulated as continuous variables and were updated by multiplying their size by their fitness to get new population sizes; then these populations were re-normalized to keep the total population constant. In each generation, there is a constant probability of a single new mutation occurring. Following this method, we tracked the descendants of the initial transgenic cells (**Figure S7E**) and also followed the progress of all clones in the simulation (**Figure 7F**). An animation of the simulation results from **Figure 7F** is shown in the **Supplemental Movie**. This animation takes the clone sizes from a *non-spatial* simulation and displays them on a 2D grid for visual purposes (the spatial behavior should not be considered meaningful). Altogether, the results shown in **Figure 7F** suggest that simulations of the basic rules above are capable of reproducing the growth and decline behavior observed in the experiment.

To confirm that these qualitative results are indeed a consequence of the basic rules rather than the exact implementation method, we applied another, independent, algorithm for simulation. This second algorithm is based on a Moran process (Moran, 1957). In this method, there is a finite population of N cells and at each step one cell is selected to replicate itself (the size of the clone it belongs to grows by one cell) and one cell is selected to die (the corresponding clone size decreases by one cell). The probability of selecting a cell to replicate is in proportion to its fitness; the selection of the cell to die is independent of fitness. Mutations are introduced at random at any point in between a birth-death event. We found that similar patterns of growth and decline of the transgenic descendants could be reproduced under this implementation.

Regardless of the particular implementation used, multiple simulation runs show that the decline of the transgenic population is not necessarily inevitable (**Figure S7E**). The particular parameters selected can influence the pattern of results. For example, a low mutation rate or a high initial growth rate for the transgenic cells means that the transgenic clone is more likely to take over the whole population before stronger mutations emerge to compete. We do not attempt to relate the parameters in these simulations directly to experimental results, merely to suggest that the principle of random mutation and competition *may* provide an explanation for the growth and decline of $p53^{*/wt}$ seen in the experiment.

For further details of the algorithms see the code, which is available at:
https://github.com/PHJonesGroup/Murai_etal_SI_code
